# Biological plausibility and stochasticity in scalable VO_2_ active memristor neurons

**DOI:** 10.1038/s41467-018-07052-w

**Published:** 2018-11-07

**Authors:** Wei Yi, Kenneth K. Tsang, Stephen K. Lam, Xiwei Bai, Jack A. Crowell, Elias A. Flores

**Affiliations:** HRL Laboratories, 3011 Malibu Canyon Rd, Malibu, CA 90265 USA

## Abstract

Neuromorphic networks of artificial neurons and synapses can solve computationally hard problems with energy efficiencies unattainable for von Neumann architectures. For image processing, silicon neuromorphic processors outperform graphic processing units in energy efficiency by a large margin, but deliver much lower chip-scale throughput. The performance-efficiency dilemma for silicon processors may not be overcome by Moore’s law scaling of silicon transistors. Scalable and biomimetic active memristor neurons and passive memristor synapses form a self-sufficient basis for a transistorless neural network. However, previous demonstrations of memristor neurons only showed simple integrate-and-fire behaviors and did not reveal the rich dynamics and computational complexity of biological neurons. Here we report that neurons built with nanoscale vanadium dioxide active memristors possess all three classes of excitability and most of the known biological neuronal dynamics, and are intrinsically stochastic. With the favorable size and power scaling, there is a path toward an all-memristor neuromorphic cortical computer.

## Abstract (web summary)

The neuromorphic computing based on complementary metal-oxide-semiconductor transistors holds promise for artificial intelligence, but it suffers from the trade-off between scalability and biological fidelity. Yi et al. emulate 23 types of biological neuronal behaviors using scalable VO_2_ active memristors.

## Introduction

The gap between artificial intelligence (AI) and mammal-level intelligence lies in both the architecture and the building blocks. It is unlikely that AI implemented on conventional computing platforms will eventually fill both gaps. Even if the brain’s connectivity were reproduced, artificial neurons and synapses built with non-biomimetic complementary metal-oxide-semiconductor (CMOS) circuits are not capable of emulating the rich dynamics of biological counterparts without sacrificing the energy consumption and size. CMOS-based neuromorphic computing (NMC) hardware suffer from the cost-fidelity dilemma i.e., scalability and biological fidelity are not simultaneously achievable. Although spike domain algorithms are energy savvy, their performance is handicapped by the poor scalability of neuron and synapse building blocks. A survey of chip-scale deep-learning image inference (See Supplementary Fig. 1) reveals that graphic processing units (GPUs) are the state-of-the-art (SOA) in throughput. However, the higher throughput comes at the cost of lower energy efficiency (EE). By contrast, NMC processors are the SOA in EE, but their throughput is much lower than GPUs’. Regardless of architecture, a universal boundary looks to exist for the throughput·EE product of all CMOS processors, which is likely limited by the CMOS device physics.

Memristors provide an alternative approach to advance NMC. The nonvolatile, stochastic, and adaptive passive memristor offers an electronic analog to biological synapses. The superb scalability of memristor crossbars projects towards the synapse density of the brain (10^10^ cm^−2^)^1,2^. Recently, biologically plausible self-learning and spike-timing dependent plasticity (STDP) were demonstrated^3,4^. A complementary device, the active memristor, can be used to construct an electronic equivalent of biological neurons. Active memristors show volatile resistive switching and are locally active within a hysteretic negative differential resistance (NDR) regime in current-voltage characteristics. The NDR provides signal gain needed for signal processing. Recently, active memristor based spiking neurons were demonstrated^5^ with biomimetic properties such as all-or-nothing spiking, refractory period, and tonic spiking and bursting. However, these demonstrations were interpreted by leaky integrate-and-fire (LIF) models^6^. LIF neurons possess much fewer neuro-computational properties^7^ than biologically-accurate models, e.g., the Hodgkin Huxley (HH) model^8^. Network-wise, most of the prior art pursued hybrid approaches that combine passive memristors with software neurons or CMOS neurons^9–12^. Such hybrid approaches promise bio-competitive synaptic scalability, but still suffer the poor size and power scalability of Si neurons (See Supplementary Fig. 2). The lack of built-in stochasticity for CMOS neurons is a handicap for achieving complex computational tasks, e.g., Bayesian inference, that require stochastic neuronal populations^13^.

In this article, using scalable vanadium dioxide (VO_2_) active memristors, we show that memristor neurons possess most of the known biological neuronal dynamics. Twenty three types of biological neuronal behaviors are experimentally demonstrated, including tonic spiking and bursting, phasic spiking (Class 3 excitability) and bursting, mixed-mode spiking, spike frequency adaptation, Class 1 and Class 2 excitabilities, spike latency, subthreshold oscillations, integrator, resonator, rebound spike and burst, threshold variability, bistability, depolarizing after-potential, accommodation, inhibition-induced spiking and bursting, all-or-nothing firing, refractory period, and excitation block. The built-in stochasticity is demonstrated by stochastic phase-locked firing, aka skipping. Finally, our simulations show that the dynamic and static power scaling of memristor neurons project toward biologically competitive neuron density and EE.

## Results

### Locally active memristors

**Fig. 1:**
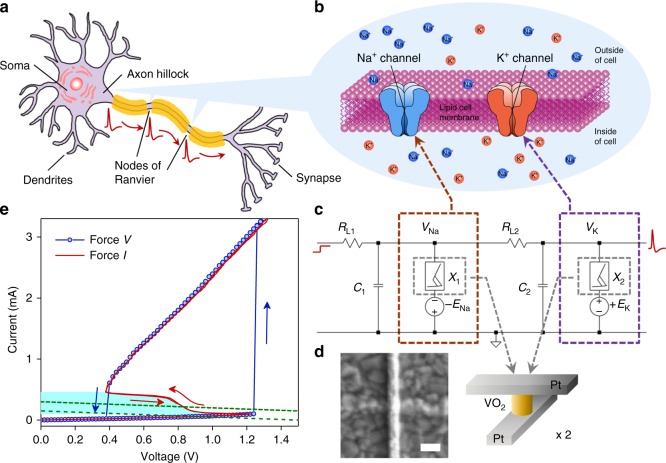
Circuit diagram of a biomimetic active memristor neuron and active memristor device characteristics. **a** Schematic structure of a biological neuron, showing that an action potential is fired near the axon hillock (under sufficient input stimulus) and propagates along the cell axon towards the output synapses. **b** Mechanism of voltage-gated Na^+^ and K^+^ ion flows across the cell membrane that accounts for the action potential generation and repetition across the nodes of Ranvier (myelin-sheath gaps). A similar mechanism exists in neurons that lack a myelin sheath. **c** Basic circuit topology of a two-channel active memristor neuron to emulate the neuronal dynamics in (**b**). A voltage-gated Na^+^ (K^+^) channel is emulated by a negatively (positively) d.c. biased active memristor device, which is closely coupled with a local membrane capacitor *C*_1_ (*C*_2_) and a series load resistor *R*_L1_ (*R*_L2_). **d** Schematic structure and a scanning electron micrograph of a typical VO_2_ active memristor nano-crossbar device (*X*_1_ or *X*_2_ in (**c**)). Scale bar: 100 nm. **e** Typical two-terminal quasi d.c. voltage-controlled (force *V*) and current-controlled (force *I*) *I*–*V* characteristics of a VO_2_ active memristor device. A wide hysteresis loop exists in the voltage-controlled mode due to the Mott transitions (blue arrows). The same Mott transitions are manifested by an “S” shaped negative differential resistance (NDR) regime (highlighted by cyan color) with a much narrower hysteresis (red arrows) in the current-controlled mode. In its resting state, the resistor load line for memristor *X*_1_ (or *X*_2_) intersects with its *I*–*V* loci outside the NDR regime (green dotted line). An input current or voltage stimulus can shift the load line into the NDR regime (green dashed line) and elicit an action potential generation (spiking)

Chua’s memristive theorem^14^ proves that a pinched hysteresis in the *I*–*V* (*I*: current, *V*: voltage) loci is the only required fingerprint of a memristor. Although a canonical memristor is a passive one-port (two-terminal) circuit element, the same theorem can be applied to a class of one-port devices that exhibit a hysteretic negative differential resistance (NDR, i.e., $\frac{\mathrm{d}V}{\mathrm{d}I} \,< \, 0$) in certain region of the *I*–*V* loci. If the circuit operating point lies within the NDR regime, e.g., when a resistor load line intersects with the *I*–*V* of the nonlinear device in the NDR regime, the device becomes locally active (see Fig. 1e). A locally active (active hereinafter) memristor can produce an a.c. signal gain greater than 1 and serve as an amplifier, or excite oscillations in appropriate circuits having reactive elements (see Supplementary Fig. 3 and Note 1). Therefore, active memristors can be used as scalable gain elements in information processing. Local activity, together with edge of chaos, are two basic properties for neurons. Chua showed that the locally active domain in the activity diagram of a HH cell is the origin of spikes^15^, and it is derived from the locally active regime in the *I*–*V* loci of voltage-gated ion channels.

We limit the discussions to active memristors that show current-controlled NDR (“S”-shaped *I*–*V* loci in current sweeps), since they are at a high-resistance state when powered off, thus offering low standby power dissipation. Not every hysteretic NDR device is an active memristor, though. A counter example is silicon thyristor, which exhibits a hysteretic NDR but the *I*–*V* loci does not pass through the origin (not “pinched”), and therefore it is not a genuine memristor^16^. In contrast to a passive memristor, in an active memristor the hysteresis collapses before the external voltage is removed, therefore the memory effect is transient. Several mechanisms can produce “S”-NDR. It may show up in the electroforming of a passive oxide memristor^17^ due to self-heating induced conductivity instability. However, such an NDR is irreversible and vanishes after the device is electroformed. Ovonic threshold switches (OTS) made of amorphous chalcogenides have reversible “S”-NDR^18,19^, which can be explained by trap-limited conduction that increases exponentially under high electric field^20^. OTS are being exploited as an access selector device in memristor crossbars to mitigate the sneak-path issue^19^, but their endurance is limited by material degradation due to field stress. A more promising class of “S”-NDR devices is Mott memristors based on thermodynamically-driven Mott insulator-to-metal transition (IMT) in certain transition metal oxides. Mott memristors are more robust since there is no high field or chemical redox reaction involved in the quantum phase transition. Nanoscale niobium dioxide (NbO_2_) Mott memristor electroformed from amorphous niobium pentoxide (Nb_2_O_5_) was reported^21^. However, we found that such an electroforming process, in our case forming VO_2_ from amorphous vanadium pentoxide (V_2_O_5_), produced void in the oxide film and electrode damage, likely due to O_2_ gas released in the reduction of V_2_O_5_. Electroformed VO_2_ devices showed poor yield and large variations in switching characteristics, and hence are impractical for circuit applications^22^. In this work, we have developed electroforming-free VO_2_ active memristors on CMOS-compatible SiN_x_-coated silicon substrates with typical yield > 98% (See Methods and Supplementary Figs. 4–8). These electroforming-free VO_2_ nano-crossbar devices show low device-to-device variability with < 13% coefficient of variation in switching threshold voltage for devices with critical dimension from 50–600 nm, and high switching endurance of > 26.6 million cycles without discernible change in device *I*–*V* characteristics. The electroforming-free VO_2_ device technology expedited the development of active memristor neuron circuitries that can emulate most of the known neuronal dynamics and cleared the path toward large-scale integrated circuit (IC) implementations. Moreover, VO_2_ is a superior Mott memristor than NbO_2_ in both switching speed and switching energy. Simulated Mott transition in VO_2_ is 100 times faster than in NbO_2_, and only consumes about one-sixth (16%) of the energy (See Supplementary Fig. 9, Table 1, Note 1 and Note 2).

### Circuit topology and spiking behaviors of VO_2_ neurons

Schematic structure and action potential generation mechanism in a biological neuron is shown in Fig. 1a, b. The basic circuit topology of a single-compartment VO_2_ active memristor neuron is shown in Fig. 1c. The prototype circuit consists of two resistively coupled relaxation oscillators, each having a d.c.-biased active memristor (*X*_1_ or *X*_2_), a parallel membrane capacitor (*C*_1_ or *C*_2_), and a load resistor (*R*_L1_ or *R*_L2_). The oppositely-energized (polarized) memristors *X*_1_ and *X*_2_ emulate the voltage-gated Na^+^ and K^+^ membrane protein ion channels, respectively. Similar circuit concepts emerged in early 1960s, e.g., the “Neuristor” axon first proposed by H. Crane^23,24^, but the scalability of these early proposals were poor due to the needs of either inductors^25^ or bipolar thyristors^26^. Scalable Neuristor circuitries can be realized by Mott memristors due to their superior 4*F*^2^/*N* (*F*: half pitch, *N*: number of stacked layers) scalability^5^. In our design, the two membrane capacitors are grounded instead of d.c. biased^5^, so that the voltages across them are the actual local membrane potential across the nerve cell membrane. This is consistent with the original HH neuron model, except that the single membrane capacitor is divided into two, each closely coupled with a voltage-gated membrane ion channel. Unbiased capacitors also offer more flexibility in IC design. The two-stage circuit has the same dimensionality as the HH model. Its dynamics is described by four coupled first-order differential equations that solve four state variables (*u*_1_, *u*_2_, *q*_1_, *q*_2_), wherein *u*_1_ and *u*_2_ are the normalized metallic channel radii of the memristors and *q*_1_ and *q*_2_ are the charges stored on the capacitors^5^. Since *q*_1_ and *q*_2_ are connected to the local membrane potentials *V*_Na_ and *V*_K_ by the linear relationships of *q*_1_ = *C*_1_*V*_Na_ and *q*_2_ = *C*_2_*V*_K_, the four state variables can be rewritten as (*u*_1_, *V*_Na_, *u*_2_, *V*_K_) (See Supplementary Note 3). A benefit of this transformation is that *V*_Na_ and *V*_K_ are straightforward to measure experimentally. Some characters of spiking dynamics, e.g., limit cycle oscillation and bifurcation, can be revealed in the two-dimensional *V*_Na_–*V*_K_ phase plane^6^. Several groups have used a single-stage Pearson-Anson relaxation oscillator as an LIF neuron^27–30^. Having only two state variables, such neurons may provide some simple spiking functionalities, but lack the heterogeneity and ergodicity of neuronal dynamics needed for more sophisticated neural networks.

**Fig. 2:**
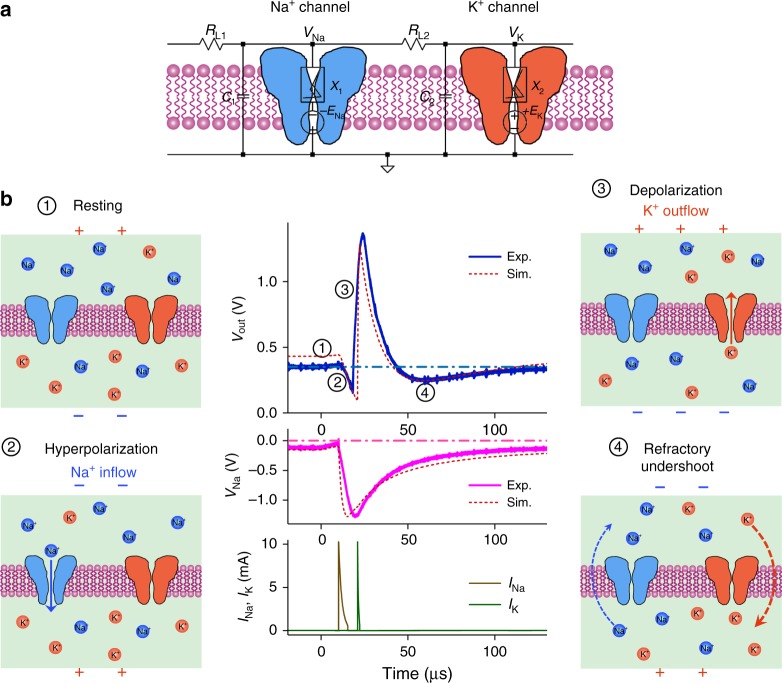
Action potential generation in a VO_2_ active memristor neuron. **a** Circuit diagram of a VO_2_ memristor neuron, consisting of two resistively coupled Pearson-Anson relaxation oscillators (*R*_L1_, *C*_1_, *X*_1_ and *R*_L2_, *C*_2_, *X*_2_, respectively). The negatively-biased memristor *X*_1_ acts as the voltage-gated Na^+^ channel, and the positively-biased memristor *X*_2_ acts as the voltage-gated K^+^ channel. Capacitors *C*_1_ and *C*_2_ are the corresponding membrane capacitances. **b** Basic steps in action potential (spike) generation of a VO_2_ neuron. (1) Resting state, in which both the Na^+^ and K^+^ channels are closed. A resting potential of 0.2–0.3 V is produced by a membrane leakage current flowing through the VO_2_ devices in their insulating state. (2) Hyperpolarization caused by the activation of the Na^+^ channel, which drives the membrane potential toward negative direction. (3) Depolarization caused by the activation of the K^+^ channel, which drives the membrane potential toward positive direction. (4) Refractory (undershoot), during which the neuron is recovering and does not respond to another stimulus. The central plots are experimental and simulated action potentials (top), the Na^+^ channel membrane potential *V*_Na_ (middle), and simulated Na^+^ and K^+^ channel currents (bottom)

The basic operational steps in experimental and simulated action potential (spike) generation are shown in Fig. 2b, with the analogous biological processes illustrated for pedagogical purpose. To be consistent with neuroscience convention, hyperpolarization means the membrane potential is driven toward negative direction, and depolarization is the opposite case. In the resting (quiescent) state, both the Na^+^ and K^+^ channels are closed (*X*_1_ and *X*_2_ are insulating). A resting potential (0.2–0.3 V) is produced by a small membrane leakage current flowing through the two oppositely-energized VO_2_ devices in insulating state. Hyperpolarization will be triggered by the activation of the Na^+^ channel if a suprathreshold input voltage or current stimulus (not shown) drives *X*_1_ into a metallic state. The Na^+^ channel membrane potential, *V*_Na_, is pulled down close to the negative d.c. bias –*E*_Na_, and its membrane capacitor *C*_1_ gets discharged. The neuron output, i.e., the K^+^ channel membrane potential *V*_K_, also gets pulled down through the coupling of *R*_L2_, but it remains above zero. The opening of Na^+^ channel is almost instantaneous due to the ultrafast Mott IMT process (seen in the simulated Na^+^ channel current), but the hyperpolarization of *V*_Na_ is much slower as it is determined by the *C*_1_ discharge time constant. Depolarization is then triggered by the activation of the K^+^ channel. It occurs when the *V*_Na_ hyperpolarization pulls *V*_K_ down low enough to make the voltage across *X*_2_ larger than its switching threshold. After *X*_2_ switches to metallic state, *V*_K_ gets pulled up close to the positive d.c. bias + *E*_K_, and its membrane capacitor *C*_2_ gets discharged. The time scale of depolarization is determined by the *C*_2_ discharge time constant. The spiking is finalized by a refractory (undershoot) period, during which the neuron is recovering and does not respond to the next input stimulus. The time scale for the action potential to fall, undershoot, then recover to resting is the longest, as *C*_1_ and *C*_2_ are slowly charged back to their resting states. In biological neurons, recovery is achieved by rebalancing the Na^+^ and K^+^ concentrations across the cell membrane by Na^+^-K^+^ pumps (conceptually shown by dashed lines) instead of voltage-gated Na^+^ and K^+^ protein channels (both are closed).

The neuron circuit shown in Fig. 1c continues to fire a train of evenly spaced spikes, or it fires periodic bursts of spikes when stimulated by a steady d.c. current input. These characteristics are known as part of the spiking behaviors for tonically active neurons (TANs). Phasically active neurons (PANs), on the contrary, may fire only a single spike at the onset of the steady d.c. current input due to transient dynamics, and remain quiescent afterwards as the system reaches the steady state. Phasic firing is known as Class 3 excitability^31^. Both TANs and PANs play important roles in the central nervous system. In a number of brain areas such as cortex, striatum, and midbrain, PANs act as differentiators or slope-detectors and are involved in a wide range of processes including motor control, coincidence detection in the auditory brainstem, cognition, and reward-related learning^32,33^. However, there is yet no demonstration of phasic spiking behaviors in active memristor neurons. We found that phasic spiking behaviors can be realized simply by replacing the load resistor *R*_L1_ with a capacitor *C*_in_, or by inserting a capacitor *C*_in_ before *R*_L1_ in the tonic neuron circuit. In other words, the main difference between TANs and PANs is that TANs have resistively coupled dendritic inputs and PANs have capacitively-coupled dendritic inputs. Otherwise both types of neurons share the same circuit topology. If a capacitor *C*_in_ is placed in parallel with the load resistor *R*_L1_, the circuit turns into a mixed-mode neuron, and fires a phasic burst followed by a train of tonic spiking when stimulated by a steady d.c. current input. In biological neurons, Class 3 phasic behavior is attributed to a subthreshold K^+^ current, acting as a dynamic negative feedback to preclude spiking if the input rises too slowly, and the neuron shows no bifurcation to repetitive spiking no matter how strong the input is^34^.

**Fig. 3:**
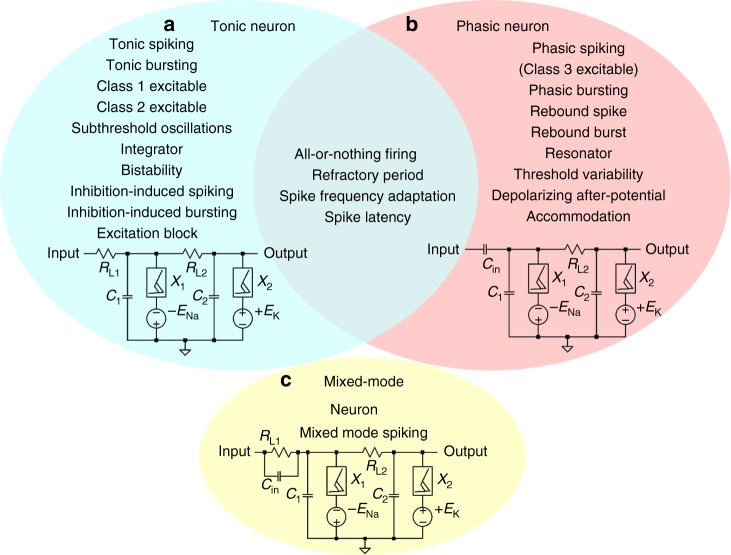
Three active memristor prototype neuron circuits and their experimentally demonstrated spiking behaviors. **a** Tonic excitatory neurons, with a resistive coupling to dendritic inputs, show tonic spiking (Supplementary Fig. 13), tonic bursting (Supplementary Fig. 14), Class 1 excitable (Fig. 4g), Class 2 excitable (Fig. 4h), subthreshold oscillations (Supplementary Fig. 18), integrator (Supplementary Fig. 19), bistability (Supplementary Fig. 20), inhibition-induced spiking (Supplementary Fig. 21), inhibition-induced bursting (Supplementary Fig. 22), and excitation block (Supplementary Fig. 23). **b** Phasic excitatory neurons, with a capacitive coupling to dendritic inputs, show phasic spiking, i.e., Class 3 excitable (Supplementary Fig. 25), phasic bursting (Supplementary Fig. 26), rebound spike (Supplementary Figs. 27–29), rebound burst (Supplementary Fig. 30), resonator (Supplementary Fig. 24), threshold variability (Supplementary Fig. 31), depolarizing after-potential (Supplementary Fig. 32), and accommodation (Supplementary Fig. 33). Other biological neuron spiking behaviors, including all-or-nothing firing (Supplementary Fig. 10), refractory period (Supplementary Figs. 11 and 12), spike frequency adaptation (Supplementary Figs. 15 and 16), and spike latency (Supplementary Fig. 17), are shared properties of both tonic and phasic neurons. **c** mixed-mode neurons, with both resistive and capacitive couplings (*R*_L1_, *C*_in_ in parallel) to dendritic inputs, show mixed-mode spiking (Supplementary Fig. 34) behavior

**Fig. 4:**
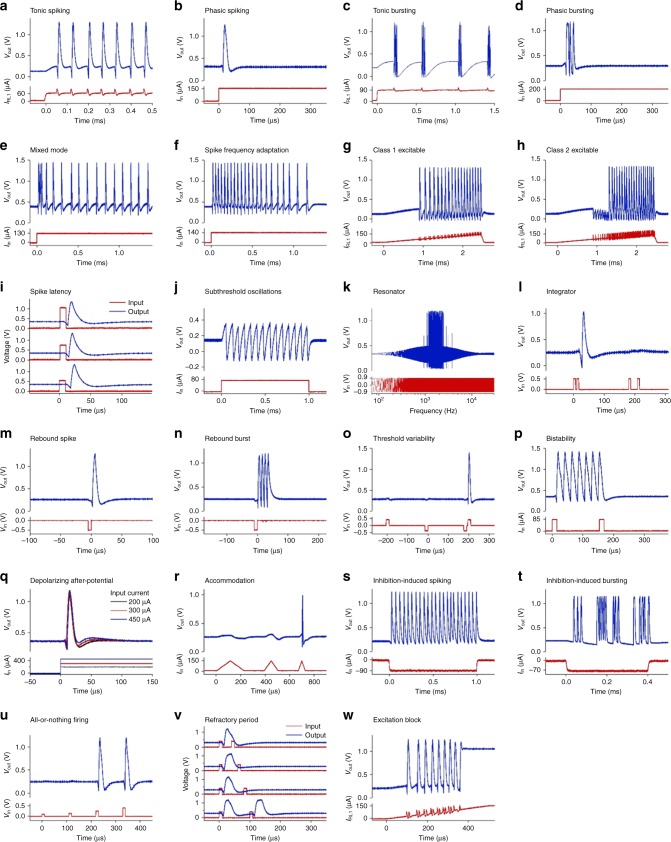
The 23 biological neuron spiking behaviors experimentally demonstrated in single VO_2_ active memristor neurons. **a** Tonic spiking. **b** Phasic spiking. **c** Tonic bursting. **d** Phasic bursting. **e** Mixed mode. **f** Spike frequency adaptation. **g** Class 1 excitable. **h** Class 2 excitable. **i** Spike latency. **j** Subthreshold oscillations. **k** Resonator. **l** Integrator. **m** Rebound spike. **n** Rebound burst. **o** Threshold variability. **p** Bistability. **q** Depolarizing after-potential. **r** Accommodation. **s** Inhibition-induced spiking. **t** Inhibition-induced bursting. **u** All-or-nothing firing. **v** Refractory period. **w** Excitation block. All the behaviors are measured from a single tonic, phasic, or mixed-mode neuron circuit that consist of only 2 VO_2_ active memristors and 4 or 5 passive R, C elements. For more details, see Supplementary Figs. 10–34

Figure 3 shows the three VO_2_ active memristor prototype neuron circuits and the lists of their experimentally demonstrated biological neuron spiking behaviors. Experimental circuit parameters are listed in Supplementary Table 2. Four basic biological neuron spiking behaviors, including all-or-nothing firing (See Supplementary Fig. 10), refractory period (See Supplementary Figs. 11 and 12), spike frequency adaptation (See Supplementary Figs. 15 and 16), and spike latency (See Supplementary Fig. 17), are shared properties of both tonic and phasic neurons. Besides these shared spiking behaviors, In TANs, ten unique spiking behaviors are observed, including tonic spiking (See Supplementary Fig. 13), tonic bursting (See Supplementary Fig. 14), Class 1 excitability (See Fig. 4g), Class 2 excitability (See Fig. 4h), subthreshold oscillations (See Supplementary Fig. 18), integrator (See Supplementary Fig. 19), bistability (See Supplementary Fig. 20), inhibition-induced spiking (See Supplementary Fig. 21), inhibition-induced bursting (See Supplementary Fig. 22), and excitation block (See Supplementary Fig. 23). In PANs, eight unique spiking behaviors are observed, including phasic spiking, i.e., Class 3 excitability (See Supplementary Fig. 25), phasic bursting (See Supplementary Fig. 26), rebound spike (See Supplementary Figs. 27–29), rebound burst (See Supplementary Fig. 30), resonator (See Supplementary Fig. 24), threshold variability (See Supplementary Fig. 31), depolarizing after-potential (See Supplementary Fig. 32), and accommodation (See Supplementary Fig. 33). Together with the mixed-mode spiking behavior observed in mixed-mode neurons (See Supplementary Fig. 34), we have observed 23 types of known biological neuron spiking behaviors.

**Fig. 5:**
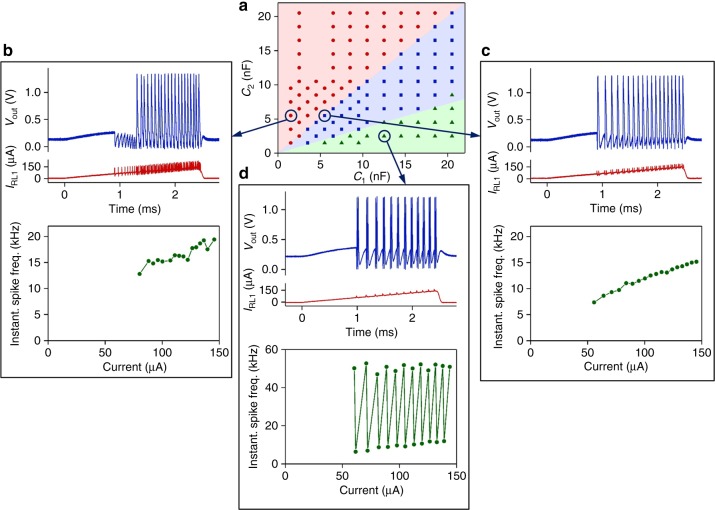
Capacitance-dependent operating regimes in a tonic VO_2_ active memristor neuron. **a** Diagram of operating regimes determined by the values of *C*_1_ and *C*_2_ membrane capacitors. When *C*_2_ > *C*_1_, the neuron exhibits Class 2 excitable spiking and subthreshold oscillations (see (**b**)). When *C*_2_ < *C*_1_, the neuron exhibits Class 1 excitable spiking (see (**c**)). When *C*_2_ < 0.35*C*_1_, the neuron exhibits Class 1 excitable bursting (see (**d**)). Various combinations of *C*_1_ and *C*_2_ are sampled (colored dots) by measuring time dependence of neuron output with a linearly ramped input current. (**b**–**d**) Typical neuron input and output vs. time (top panels), and the current-dependence of instantaneous spike frequency (bottom panels) sampled from Class 2 excitable spiking, Class 1 excitable spiking, and Class 1 excitable bursting regimes, respectively

Figure 4 summarizes the 23 experimentally demonstrated spiking behaviors in VO_2_ neurons. All the behaviors are measured from a single tonic, phasic, or mixed-mode neuron circuit that consist of only 2 VO_2_ active memristors and 4 or 5 passive R, C elements. The VO_2_ memristors are homogeneous in the sense that they have the same size (100 × 100 nm^2^) and are fabricated on the same wafer, with small spreading in switching characteristics. The heterogeneity in spiking dynamics is achieved by controllable circuit parameters, i.e., values of R, C elements or input-stage impedance. One can also control the area ratio of the two VO_2_ devices to achieve asymmetry in the emulated Na^+^ and K^+^ ion channels. In comparison, a CMOS artificial neuron constructed with nearly 1300 logic gates (each gate uses a minimum of two transistors) replicated 11 biological neuron behaviors using single neurons, and another 9 behaviors using 2 or 3 neurons^35^. The stark contrast in active device counts between memristor neurons and CMOS neurons to achieve similar level of biological fidelity is a manifestation of the importance of native biomimeticity. Controllability of neuron spiking characteristics is important for network design and neural coding. Spike-timing properties, e.g., tonic spike frequency (See Fig. 5c), tonic burst frequency and number of spikes per burst (See Supplementary Figs. 14k and l), and spike latency (See Supplementary Fig. 17c), can be directly controlled by the capacitor values or the strength of input stimuli.

In Hodgkin’s classification, there are three basic classes of neuron excitability that can be discerned by spiking patterns: Class 1, Class 2, and Class 3^31^. The nonlinear dynamical mechanism responsible for each class of excitability is reasonably well understood. Class 3 excitability, or phasic spiking, has been discussed above. A closer look at tonic neurons found that they can be converted between being Class 1 and Class 2 excitable, simply by adjusting the Na^+^ and K^+^ membrane time constants. In Figure 5, spike patterns of a tonic VO_2_ neuron subject to a linearly ramped input current are recorded at different values of *C*_1_ and *C*_2_ membrane capacitors. Other circuit parameters are left unchanged and the same value is used for the two load resistors *R*_L1_ and *R*_L2_. Therefore the membrane time constants are determined by *C*_1_ and *C*_2_. For the cases of *C*_2_ < *C*_1_ (fast K^+^, slow Na^+^), the neuron exhibits Class 1 excitability, and shows tonic spiking (if 0.35*C*_1_ < *C*_2_ < *C*_1_) or tonic bursting (if *C*_2_ < 0.35*C*_1_). In Class 1 regime, the observed spike onset threshold and initial frequency are relatively low. Theoretically, the initial frequency can be arbitrarily low. The spike frequency increases with the strength of input current with a pronounced slope. For the cases of *C*_2_ > *C*_1_ (slow K^+^, fast Na^+^), the neuron exhibits Class 2 excitability with much larger spike onset thresholds. The spike frequency is relatively constant and insensitive to changes in the input strength. In Class 2 regime, spiking is oftentimes preluded by subthreshold oscillations with sawtooth-shaped waveforms, indicating that it is the relaxation oscillation of the K^+^ channel. In canonical models, Class 1 and Class 2 excitabilities belong to different bifurcations from stable steady state to periodic (spiking) behavior as the stimulus parameter is varied^36^. There are 4 to 6 possible types of bifurcations for each class^37^. To find out the specific bifurcations responsible for the observed classes of excitabilities, a nullcline analysis mapping the four-dimensional nonlinear system into the *V*_Na_–*V*_K_ phase plane without losing part of the dynamics is needed, in a way similar to FitzHugh-Nagumo dimensionality reduction of the HH model^25,38^. Since we experimentally observed excitation block in a Class 2 tonic VO_2_ neuron (See Supplementary Fig. 23), it is possible that a supercritical Andronov-Hopf bifurcation drives the observed Class 2 excitability^37^.

### Stochasticity of VO_2_ neurons

**Fig. 6:**
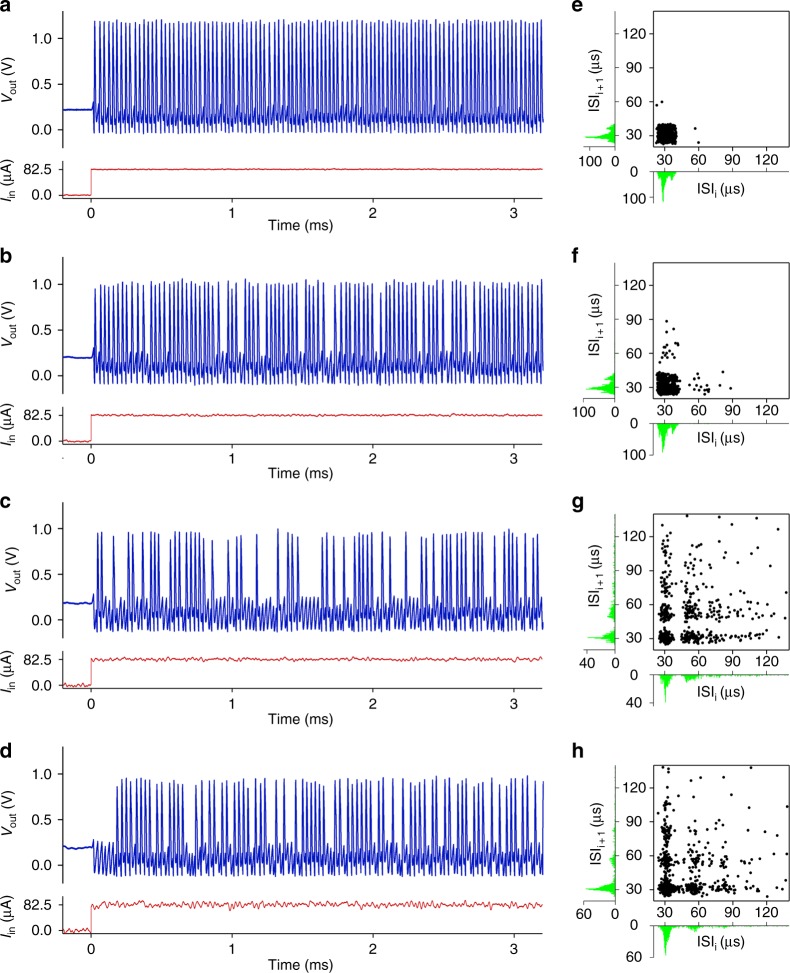
Stochastically phase-locked firing (skipping) in a tonic VO_2_ active memristor neuron. **a**–**d** Tonic spike trains excited by an input d.c. current of 82.5 µA amplitude and 35 ms duration. For clarity, only the initial sections of ~3 ms duration are displayed. White noise signals with 5 µA_pp_, 15 µA_pp_, 25 µA_pp_, and 50 µA_pp_ peak-to-peak values, respectively, are added to the current input to study its impact on the firing pattern and the correlation between consecutive interspike intervals (ISIs). **e**–**h** Joint interspike interval (JISI) scatter plots (aka return maps) of the spike trains shown in (**a**–**d**). Also shown are the histograms of the ISI distributions. The numbers of spikes used to generate the JISI plots are 1149, 1113, 620, and 754, respectively

Finally, we applied the classic stochastic spike train analysis, i.e., the joint interspike interval (JISI) analysis, to study stochasticity and correlation in spike patterns. The results show that VO_2_ memristor neurons exhibit input-noise sensitive stochastically phase-locked firing, aka skipping, in a manner similar to biological neurons^39,40^. In a first-order JISI analysis, the relationship between consecutive spike firings is inspected by analyzing a 3-spike pattern, which includes two interspike intervals (ISIs). An ISI is defined as the time difference between consecutive spikes $\tau _n = t_n - t_{n - 1}$, where *t*_*n*_ is the time of occurrence for the *n*^th^ spike. The scatter plot of (*τ*_*n*_, *τ*_*n*+1_) pairs, which is referred to as a return map (Poincaré map), is used to reveal the correlation between consecutive first-order ISIs. White noise signals with different amplitudes are superimposed on a steady current clamp (82.5 µA) input for a tonic VO_2_ neuron, and the excited spike trains within a 35 ms time duration are recorded. Figure 6 shows the measured spike patterns (only the initial sections of ~ 3 ms are shown for clarity) and JISI return maps at peak-to-peak white noise amplitudes in the range of 5 µA_pp_ to 50 µA_pp_. When the input noise is low (Fig. 6a), the neuron exhibits regular tonic spiking with ISIs tightly clustered around a predominant fundamental value (ISI median = 29.3 µs). As the input noise increases (Fig. 6b), sporadic drop-outs in firing start to emerge, while most of the firing is still clustered around the fundamental ISI. At even higher noise levels (Fig. 6c, d), the neuron exhibits irregular spiking with many drop-outs, resulting in widely scattered JISI pairs in return maps. The JISI pairs cluster around regular grids roughly at multiples of the fundamental ISI, which is also evident in the ISI histograms. Similar arrhythmic firings, or skipping, have been observed in biological neurons such as thermosensitive mammalian cold receptors^41^. These receptors show irregular spikes that are phase-locked to an underlying periodic oscillation, with a random integer number of oscillation cycles skipped between spikes. The firing irregularity increases with temperatures, consistent with our case of elevated input noise. The fundamental ISI, however, is robust against noise and does not shift or vanish. When the firing is missed, the neuron still exhibits subthreshold oscillations with sawtooth waveforms. Scatter recurrence plots of adjacent spike amplitudes (See Supplementary Figs. 35a–f) show that spike amplitude also develops irregularity and skewness as the input noise rises. The mean spike amplitude first decreases quickly with the input noise, then partially recovers at input noise higher than ~20 µA_pp_. A similar trend is seen in the skewness of spike amplitude distribution (See Supplementary Fig. 35g).

## Discussion

For memristor neurons to be biologically competitive, their EE needs to be > 10^13^ spikes/J (energy use < 0.1 pJ/spike), and their area needs to be < 100 µm^2^ (See Supplementary Fig. 2). We use SPICE simulations to analyze the dynamic and static power scaling of tonic VO_2_ neurons (See Supplementary Figs. 2, 36, 37, and Note 4). Our simulations show that at 1 fF μm^−2^ specific membrane capacitance (a value easily attainable by using common dielectrics), VO_2_ neurons show superior EE–area scaling than the best-case simulated HH cells at neuron sizes smaller than 70 μm^2^, and can surpass the estimated human brain EE of 1.8 × 10^14^ spikes/J (or 5.6 fJ/spike energy use) at neuron sizes smaller than 3 μm^2^. The results show that it is feasible for VO_2_ neurons to achieve biologically competitive EE and area.

In our case, a variety of neuronal dynamics is achieved by customizing the passive R, C elements (and circuit topology) without the need of varying VO_2_ device parameters. This scheme simplifies the IC design and fabrication, and memristor neurons with fixed R, C elements can exhibit spike frequency adaptation. To achieve more adaptivities in neuronal dynamics, such as an evolution from Class 1 excitability to Class 2 excitability, one may introduce passive memristors or memcapacitors^42^ in lieu of fixed R, C elements, if integration of memristor and memcapacitor technologies on the same substrate can be resolved.

Very recently, unsupervised all-memristor learning and pattern classification have been demonstrated using single-stage memristor neurons^30^. We have simulated a simple one-neuron one-synapse circuit using a VO_2_ model neuron and a TaO_x_ passive memristor model^3^ (See Supplementary Fig. 38). Without needing to adjust the VO_2_ or TaO_x_ model parameters, simulated TaO_x_ synaptic weight (conductance) can be continuously increased or decreased by the spikes sent from the VO_2_ neuron. This shows the feasibility of analog potentiation and depression as the precursors for biologically plausible STDP learning. All-memristor neural networks with unsupervised causal STDP learning may provide compelling solutions to computationally hard problems such as Bayesian reasoning^43^ in highly parallel and energy-efficient fashion.

A potential issue for VO_2_ neurons is that the Mott transition at near 67 °C posts a stringent requirement for core-level and chip-level thermal management, especially if an on-chip CMOS co-processor is involved. This risk can be mitigated by introducing a dopant that may lift the IMT critical temperature (*T*_C_) higher. *T*_C_ ~96 °C has been reached with 5.9 at % Ge doping in sputtered VO_2_ films^44^.

Finally, since both active memristor neurons and passive memristor synapses are fabricated from deposited thin-film structures, it is feasible in principle to vertically stack repeated pairs of memristive neurosynaptic cores to directly map to the brain cortical layers. A possible fabrication procedure for stackable integrated memristor neuron is shown in Supplementary Fig. 39. The proposed integrated neuron only requires up to three layers of interconnect metals. Passive memristor synapse array can be directly stacked on top. Such a pseudo-three-dimensional (two-and-a-half-dimensional) connectivity cannot be easily achieved using conventional CMOS technology.

## Methods

### VO_2_ device fabrication

The VO_2_ active memristor devices for the experimental demonstration were fabricated in house using electron-beam lithography, thin-film deposition and liftoff. Bottom electrodes of 50–600 nm width and 30 nm thickness (5 nm Ti/25 nm Pt) were patterned on silicon nitride covered silicon substrates with a liftoff process. This was followed by a blanket deposition of 100-nm-thick polycrystalline VO_2_ films deposited by reactive sputtering. Finally, top electrodes of 50–600 nm width and 75 nm thickness (5 nm Ti/70 nm Pt) were patterned with a liftoff process perpendicular to the bottom electrode to form the 50 × 50 nm^2^ to 600 × 600 nm^2^ metal/VO_2_/metal crossbar junction. The nearly-pure monoclinic VO_2_ phase in the sputtered oxide films is confirmed by comprehensive structural and compositional characterizations, including grazing incidence X-ray diffraction, X-ray photoemission spectroscopy, Rutherford backscattering spectroscopy, and secondary ion mass spectroscopy (See Supplementary Fig. 4). High-resolution transmission electron microscopy reveals a sharp interface between the columnar VO_2_ grains and the amorphous SiN_x_ substrate without sign of interface roughening or interfacial layer. The monoclinic VO_2_ phase is further confirmed by selected area electron diffraction (See Supplementary Fig. 5). In contrast to ref. ^5^, the as-deposited VO_2_ films do not require electroforming, and all the tested devices showed upfront Mott-transition-induced resistive switching and negative differential resistance in their very first current-voltage sweep, as shown in Supplementary Fig. 7.

### Electrical characterization

Electrical characterization of VO_2_ active memristor devices and VO_2_ neuron circuits was carried out using a probe station equipped with four source measure units, an oscilloscope, an arbitrary waveform generator, and a voltage-to-current converter (stimulus isolator). The oscilloscope voltage probes have 10 MΩ and < 4 pF input impedance. The discrete neuron circuits were constructed by connecting the two VO_2_ memristors on the same wafer to external resistors and capacitors through coaxial cables. Electrical characterizations of the completed VO_2_ devices, summarized in Supplementary Fig. 6, showed device metrics favorable for large-scale neuron ICs. Electroforming-free and volatile resistive switching are observed for almost all of the as-grown devices, as illustrated by highly uniform switching *I*–*V* traces from devices located across all the reticles (See Supplementary Fig. 7). The coefficient of variation in switching threshold voltage is 7–13% for device sizes from 50 nm to 600 nm (See Supplementary Fig. 8). The Mott-transition mechanism is supported by the temperature (*T*) dependence of the zero-bias conductance measured with the wafer mounted on a temperature-controlled heater stage (See Supplementary Fig. 6c). It shows a thermally activated transport in the insulating state at *T* < 60 °C, with a single activation energy of ~0.2 eV that is close to reported values^45^. At *T* > 60 °C, the conductance surges up as the material turns from an insulator to a metal. Typical device yield, reproducible across samples and deposition sessions, is in the range of 98–100% for the 576 devices (36 reticles and 16 devices/reticle) fabricated on a 3-inch SiN_x_/Si wafer, for crossbar devices having junction area from 50 × 50 nm^2^ to 600 × 600 nm^2^. The switching voltage threshold, ranging from 0.4 V to 1.3 V for all the devices tested so far, is size-dependent and tunable by the VO_2_ film process conditions. It also scales with the film thickness in theory, which is not yet studied experimentally. Threshold voltages of ~0.5 V or lower is competitive if compared to the supply voltage in advanced CMOS transistors. The robustness in resistive switching is demonstrated by a switching endurance of > 26.6 million pulsed-mode on/off switching cycles, without discernible change in the device *I*–*V* after the endurance test. The actual endurance number is unknown, but could be several decades higher than the instrumentation-limited measured number.

### SPICE simulations

The SPICE model used to simulate the VO_2_ switching dynamics and neuron spiking behaviors is based on the same mathematical equations outlined in ref. ^21^. See Supplementary Table 3 for the values used for the VO_2_ material parameters. All the simulated neuron behaviors used the same VO_2_ device model with a cylindrical-shaped VO_2_ conduction channel of 56 nm in radius and 100 nm in length to match the actual VO_2_ crystal volume in 100 × 100 nm^2^ sized and 100 nm-thick nano-crossbar devices used in the experiments, and only varied the values of R, C elements. Series electrode resistance of 150–500 Ω, and parallel VO_2_ channel leakage resistance of 13 kΩ–17 kΩ were included in simulations to take into account their effects on the voltage drop across the memristors and the standby current in the insulating phase.

## Electronic supplementary material

Supplementary Information

## Data Availability

The data that support the plots within this paper and other findings of this study are available from the corresponding author upon reasonable request.
