# Supplementary material for: Biological plausibility and stochasticity in scalable VO2 active memristor neurons
Source: Nat Commun. 2018 Nov 7;9:4661. doi: 10.1038/s41467-018-07052-w (PMC6220189; doi:10.1038/s41467-018-07052-w)
Supplement: Supplementary file 1 — Supplementary Information [file 41467_2018_7052_MOESM1_ESM.pdf]

Supplementary Information

Biological plausibility and stochasticity in scalable VO_2 active memristor neurons

Yi et al.

Supplementary Figures

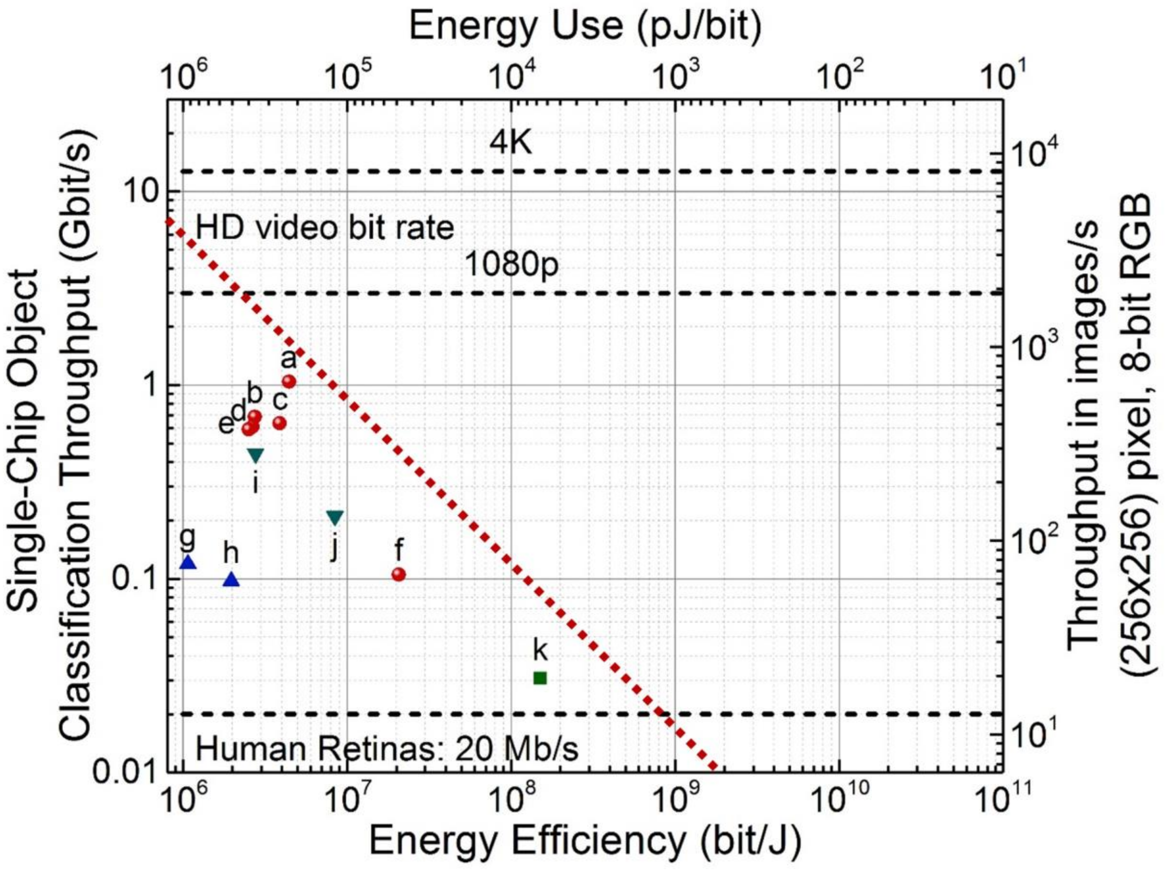

Supplementary Figure 1. Benchmarks of single-chip image object classification performance and energy efficiency (EE) in silicon GPU (data a-f), CPU (data g, h), ASIC (data i, j), and neuromorphic (NMC) (data k) processors, all running deep convolutional neural network (CNN) algorithms for inference on standard image sets. A record EE of 6.7 nJ/bit was realized in a silicon NMC processor (TrueNorth, data k)[1], which is one decade better than the record EE of 48.4 nJ/bit for GPUs (Nvidia Tegra X1, data f), and two decades better than the record EE of 509.7 nJ/bit for CPUs (Intel i7 6700K, data h)[2,3]. However, the throughput (in bit/s) of a silicon NMC chip is the lowest among the three categories, reaching only 31 Mbit/s, barely faster than a human operator (~20 Mbit/s). Best-case performance of ASIC (TPUv2, data i)[4] is comparable to that of GPUs. Dotted line is a speculated empirical boundary of the chip-level inference throughput·EE product for the surveyed technologies, i.e. the so-called 'performance-efficiency dilemma', possibly limited by the silicon CMOS device physics. The bit rates for 60Hz 1080p or 4K video are 2.99 Gb/s and 12.7 Gb/s, respectively. For a GPU to deliver inference throughput at 1080p HD bit rate, the extrapolated chip-level power consumption is ~3.8 kW. Standard AlexNet architecture and non-batched ImageNet image set (8-bit RGB images with 65536 pixels) are used in GPU and CPU benchmarking. A custom CNN architecture and CIFAR10 image set (8-bit RGB images with 1024 pixels) is used in NMC benchmarking. The reported inference throughput values in frame/s were first converted to pixel/s then to bit/s by the formula of (bit count) = (pixel count) × (color channel) × (color depth) = pixel count × 24.

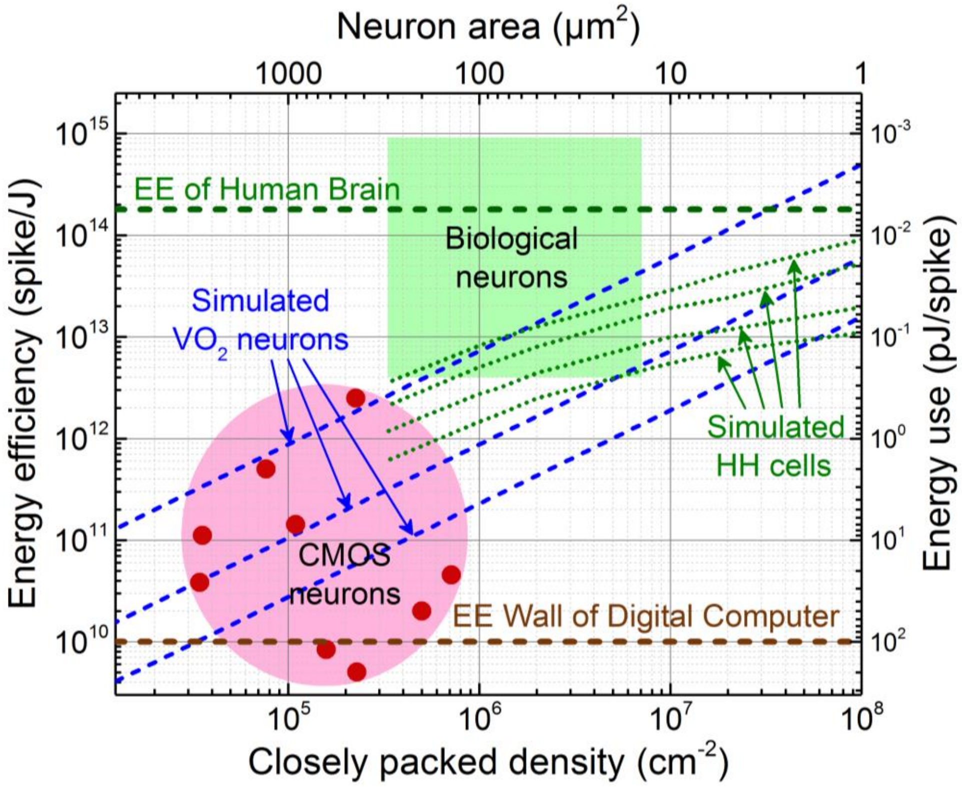

Supplementary Figure 2. The scaling of neuron energy efficiency (EE, in number of spikes per Joule) vs. neuron area. Data points encircled by the magenta ellipse are from silicon CMOS neurons published in 2008-2014[5-13], with the lowest reported energy use at 0.4 pJ/spike[9]. The estimated domain of biological neurons is outlined by the green rectangle. Simulated Hodgkin–Huxley (HH) cells (green dotted lines, simulated at ion channel density of 0.5, 1, 2, and 4 μm^{-2} respectively, from top to bottom) illustrate trends of higher EE for smaller neurons, and higher EE for lower ion channel densities[14]. Simulated VO_2 memristor neurons (blue dashed lines, at specific membrane capacitance of 1, 10, and 43 fF μm^{-2}, from top to bottom) show a similar trend of higher EE for smaller neurons (but with a higher slope of change), and higher EE for smaller specific membrane capacitance. This is understandable, since the dynamic spiking energy is proportional to the membrane capacitance (see Supplementary Fig. 36). At the same neuron (and capacitor) area, lower specific membrane capacitance translates into lower dynamic spiking energy and hence higher EE. At 1 fF μm^{-2} specific capacitance (the topmost blue line), VO_2 neurons show superior EE-area scaling than the best-case HH cells at neuron sizes smaller than 70 μm^2, and can surpass the estimated human brain EE (horizontal green dashed line) of 1.8×10^{14} spike/J (or 5.6 fJ/spike energy use) at neuron sizes smaller than 3 μm^2. Orange line is the conceived fundamental limit of EE for digital computers[15]. For simplicity, a one-to-one conversion is assumed between the EE of multiply–accumulate operations (MAC) per Joule in a digital computer and the EE of spikes per Joule in the brain, i.e. 1 MAC/J = 1 spike/J. The unit of EE used in simulated HH cells[14] is bit/ATP, which is converted to spike/J by conversion factors of 1 ATP = 10^{-19} J and 1 bit = 1 spike. In simulated VO_2 neurons, VO_2 channel radius (length) is fixed at r (L) =10 (10) nm, and the two VO_2 memristors contribute 2.3 fJ of switching energy in each spike. It is assumed that 80 % of the total neuron area is occupied by membrane capacitors. This ratio may vary with specific designs, but adjusting it will only cause a small lateral shift in the trend lines without affecting the slope and the main conclusions.

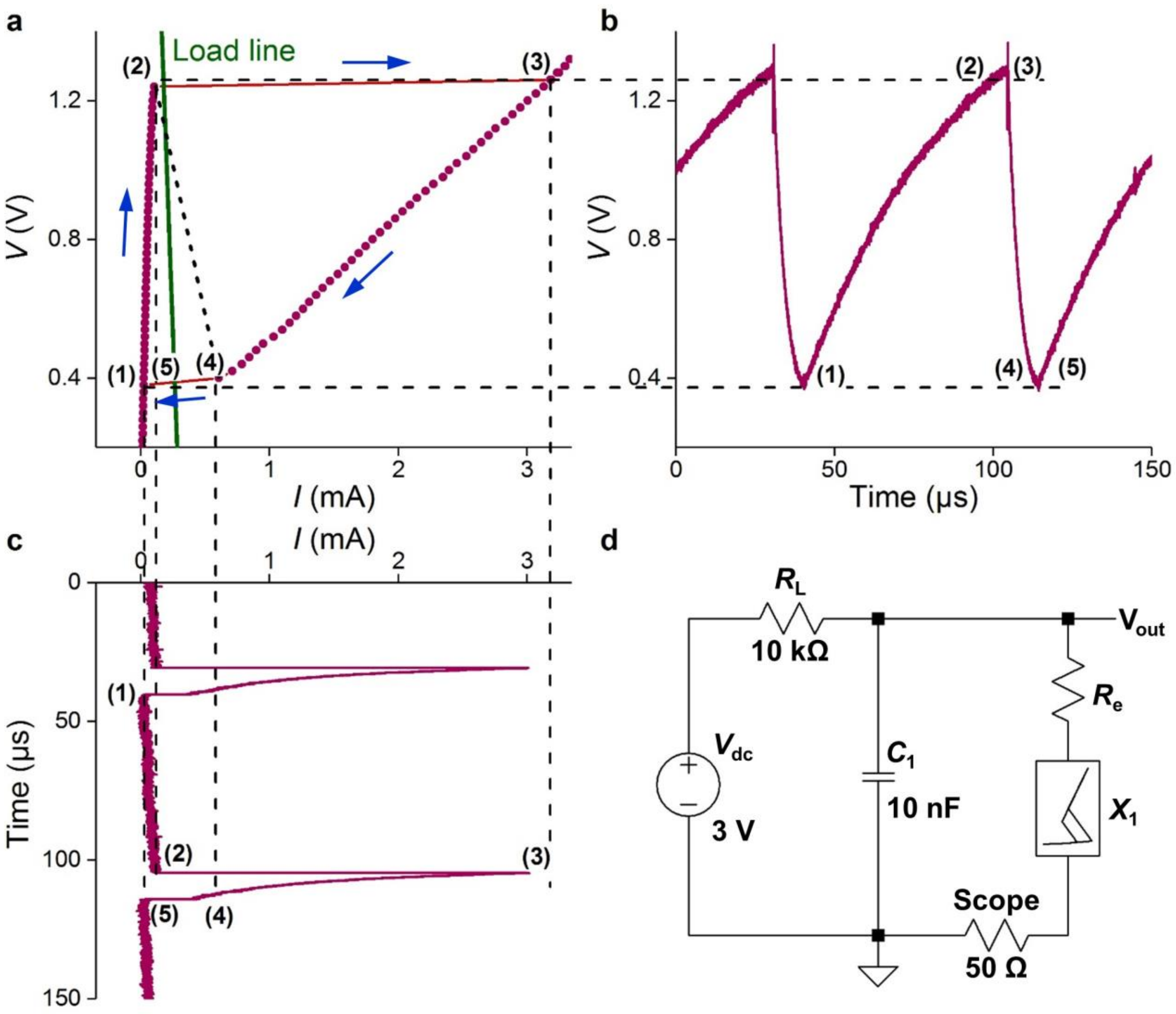

Supplementary Figure 3. Astable oscillator characteristics measured in a VO_2 Pearson-Anson relaxation oscillator. a, Two-terminal quasi-d.c. *V*–*I* characteristics (force *V*, measure *I*) of the VO_2 device X_1 in circuit **d** (including R_e) by sweeping the d.c. bias from 0 to 1.6 V and then back to 0. R_e (≈370 Ω) is the metal wire resistance in the crossbar device. To enable astable oscillations, the load line (green), defined by the d.c. bias (V_{dc}) of 3 V and the load resistor (R_L) of 10 kΩ in circuit **d**, must intersect the *V*–*I* curve in its negative resistance region connecting (2) and (4). **b**, Waveform of the output voltage (V_{out}) in circuit **d**, showing sawtooth-shaped relaxation oscillations. **c**, Waveform of the current flowing through the VO_2 device X_1 (monitored by an oscilloscope channel with 50 Ω input resistance to ground), showing Mott transitions from (2) to (3) and from (4) to (5). The actual rise or fall time in (2)–(3) and (4)–(5) transitions are much shorter than the sample interval used (2 ns). A complete cycle of astable oscillation from (1) to (5) has four stages (see arrows). From (1) to (2): switch X_1 remains open, capacitor C_1 is charged till V_{out} reaches the switching threshold of X_1. From (2) to (3): closing of X_1 causes a surge in current, but V_{out} is held constant by C_1. From (3) to (4): capacitor C_1 is discharged till V_{out} reaches the minimum holding threshold for X_1 to stay metallic. From (4) to (5): X_1 is reopened.

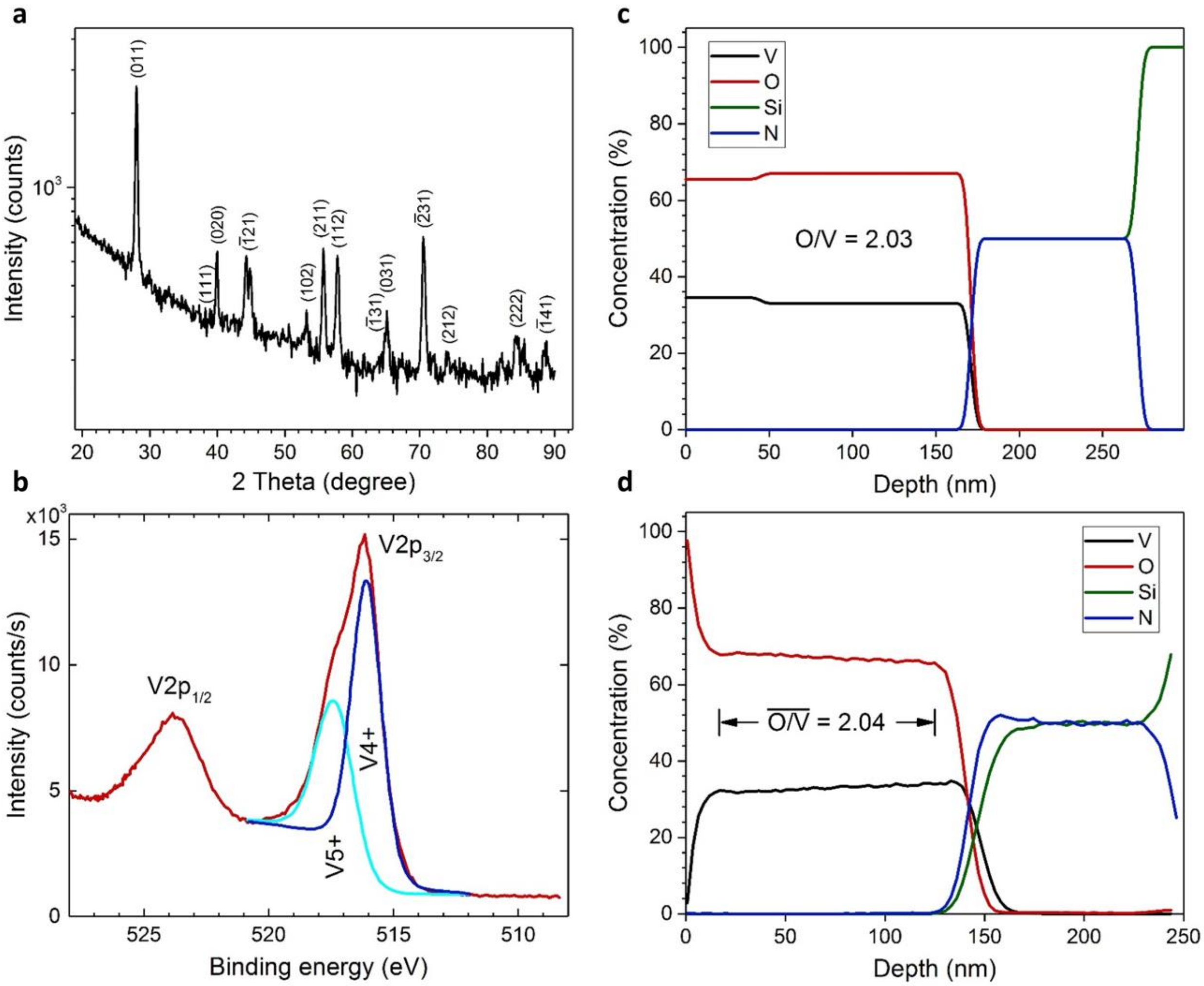

Supplementary Figure 4. Structural and compositional characterizations of a 100nm-thick VO_2 film grown on SiN_x/Si substrate. a, Grazing incidence X-ray diffraction (GIXRD) spectrum acquired at 1.54059 Å Cu Kα1 wavelength. Indexed lines are the results of a phase-identification analysis by using the whole pattern fitting method. The best match (at an R factor of 7.28 %) is found with a monoclinic VO_2 phase (space group: P21/c (14) PDF# [98-001-4290]). The relative intensities indicate some preferred orientation of the crystallites. **b**, High-resolution X-ray photoemission spectroscopy (XPS) of the V2p spectral doublet ($V2p_{1/2}$ and $V2p_{3/2}$). The $V2p_{3/2}$ peak is curve fitted in an attempt to quantify the oxidation states of V, showing a dominating V^{4+} oxidation state (64 %) with the rest of it being at V^{5+} state (36 %). Since XPS only detects the top few nm thickness of the film, the V^{5+} state was possibly due to native oxidation after the film was exposed to air. **c**, Rutherford backscattering spectrum (RBS) showing atomic concentrations of O at (67±4) % and V at (33±1) %, or a O:V ratio of 2.03:1. The thickness in RBS is estimated by assuming a density of 7.15×10^{22} atoms cm^{-3}. **d**, Secondary ion mass spectroscopy (SIMS) showing an average O:V ratio of 2.04:1 (in the depth of 40–120 nm).

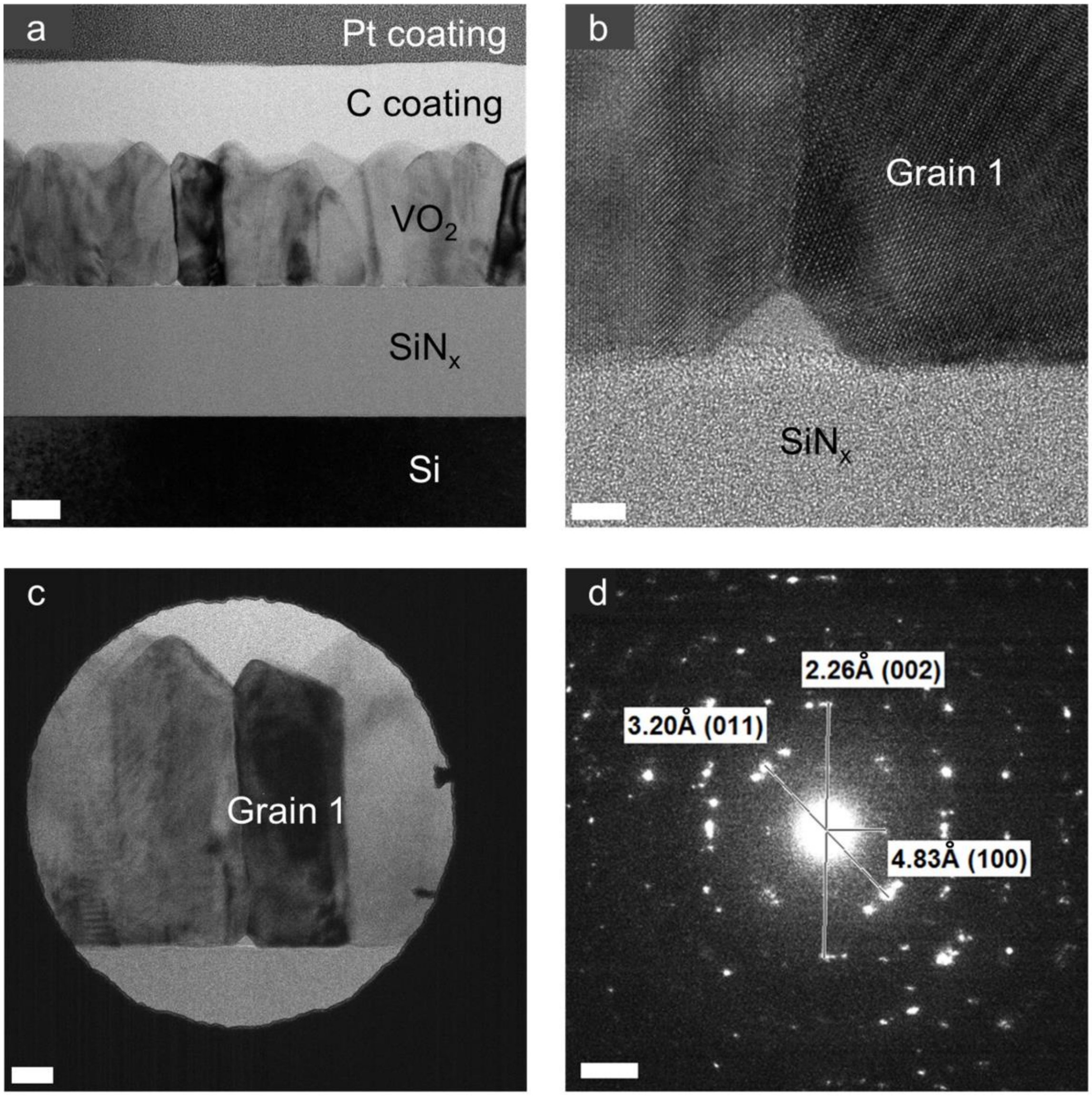

Supplementary Figure 5. Transmission electron microscopy and electron diffraction characterizations of a 100nm-thick VO_2 film grown on SiN_x/Si substrate. a, Cross-sectional bright-field transmission electron microscopy (BFTEM) image of the VO_2 sample prepared by focused ion beam cutting. The polycrystalline nature of the VO_2 film with columnar grain structures is clearly resolved. The brighter and darker contrasts seen across grain boundaries are caused by electron beam diffractions by lattice planes with a slight tilt from one grain to another. Scale bar: 50 nm. **b**, High-resolution (HRTEM) image of the VO_2/SiN_x interface. Scale bar: 4nm. **c**, BFTEM image of one particular grain ("grain 1") selected for electron diffraction study. Scale bar: 20 nm. **d**, Selected area electron diffraction (SAED) pattern of grain 1 showing diffraction spots that can be matched nearly perfectly with the d-spacing of a few low-index lattice planes, (002), (100), and (011), from a monoclinic VO_2 phase (space group: P21/c (14) PDF# [98-001-4290]). Scale bar: 2 nm^{-1}.

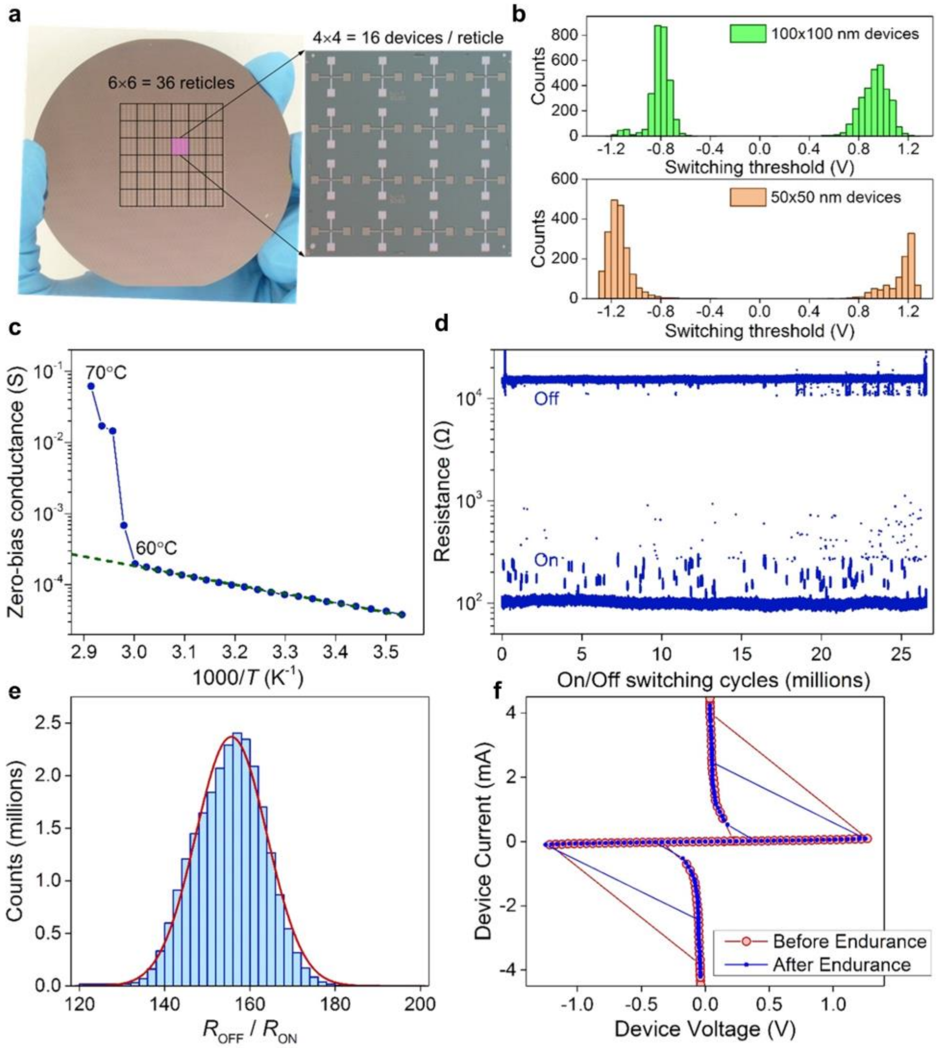

Supplementary Figure 6. Characteristics of electroform-free VO_2 active memristor devices. **a**, Photo of an array of 576 VO_2 crossbar devices (36 reticles and 16 devices in each reticle) fabricated on a 3-inch SiN_x/Si substrate. **b**, Histograms of the switching threshold voltage measured from 288 100 × 100 nm^2 VO_2 devices (top) and 288 50 × 50 nm^2 devices (bottom) fabricated on the same wafer. The mean values of switching thresholds are size-dependent and tunable by the VO_2 film process conditions. **c**, Temperature dependence of device conductance G near zero bias (50 mV) measured in a heating cycle, showing a sharp Mott transition when temperature rises above 60 °C. Thermally activated electron transport in the insulating state is shown by a least-square fit (R^2 = 0.996) by $\ln(G/T^2)$ vs. $1000/T$ (dashed line) with a single activation energy of (0.205 ± 0.003) eV. **d**, Switching endurance data of > 26.6 million pulsed-mode on/off switching cycles. All the switching events were measured without subsampling. The data show no sign of device degradation or drift in resistance values during the endurance test. **e**, Histogram of the R_{OFF}/R_{ON} resistance ratio in the endurance measurement of **d**. Red line is a Gaussian fit with R^2 = 0.99, having a center of 155.65 ± 0.15 and FWHM of 16.72 ± 0.30. **f**, Four-terminal quasi d.c. I–V characteristics (force V, measure I) of the device tested in **d** before and after the endurance test, showing no deterioration or drift in its switching characteristics.

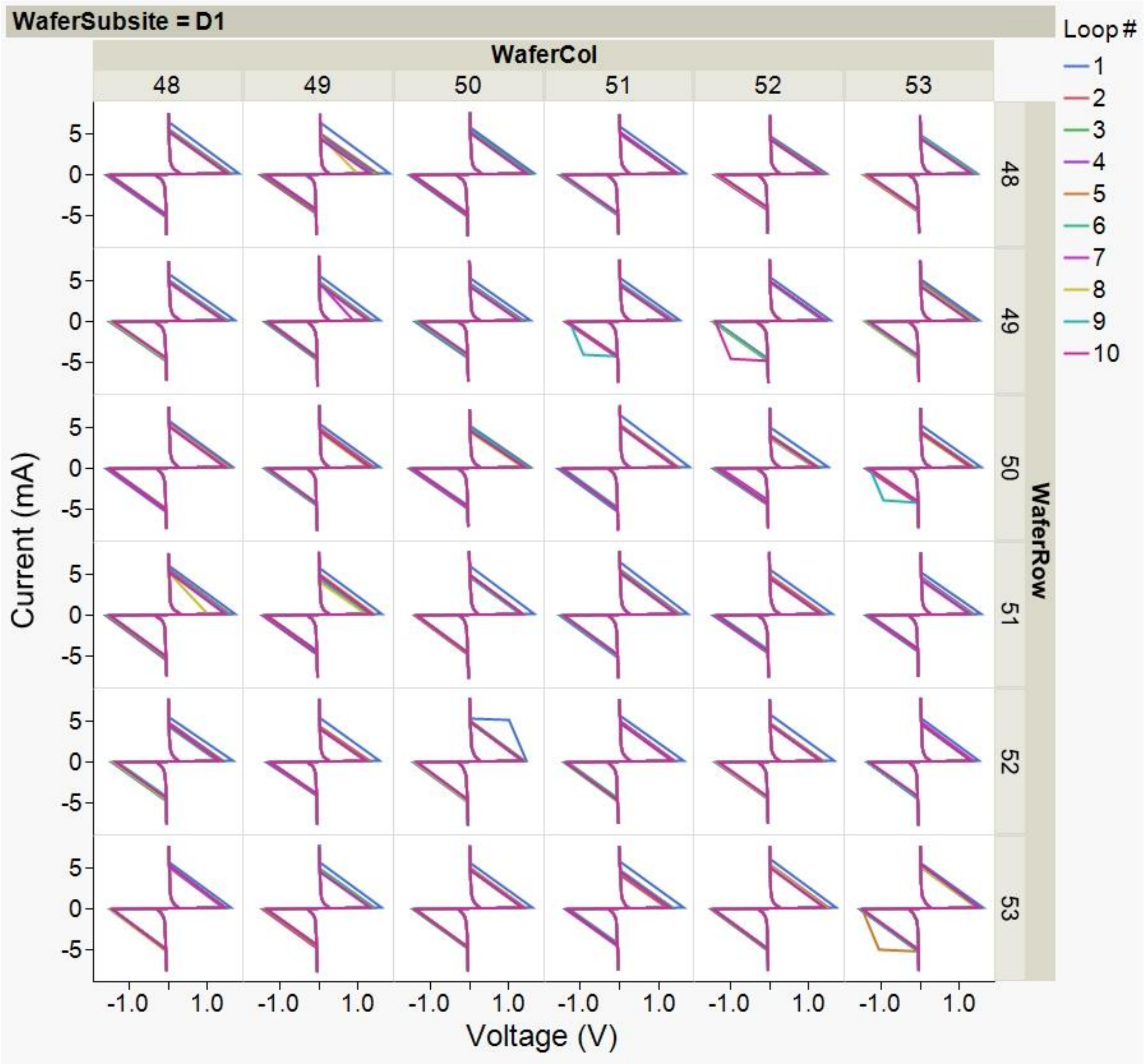

Supplementary Figure 7. Wafer-scale uniform and electroform-free switching characteristics of VO_2 devices. Plotted are 36 sets of four-terminal quasi-d.c. *I–V* traces (force *V*, measure *I*) measured from VO_2 crossbar devices, sampled one device per reticle from 36 reticles (labeled by the wafer row and column numbers, from 48 to 53) in the same wafer. All the devices are identified as WaferSubsite = D1, meaning that they are all located at the same relative position inside each reticle (D1 is the first device at the bottom left corner in each reticle, as shown in Supplementary Figure 6a). For each device, the force *V* and measure *I* sweeps is repeated 10 times at the same setting. Majority of the as-grown VO_2 devices, ~98 %, showed upfront resistive switching and NDR in the very first *I–V* sweep without the need of electroforming. The uniformity of switching is demonstrated in both the run-to-run repeatability and the low device-to-device variation in switching thresholds (see Supplementary Figure 8).

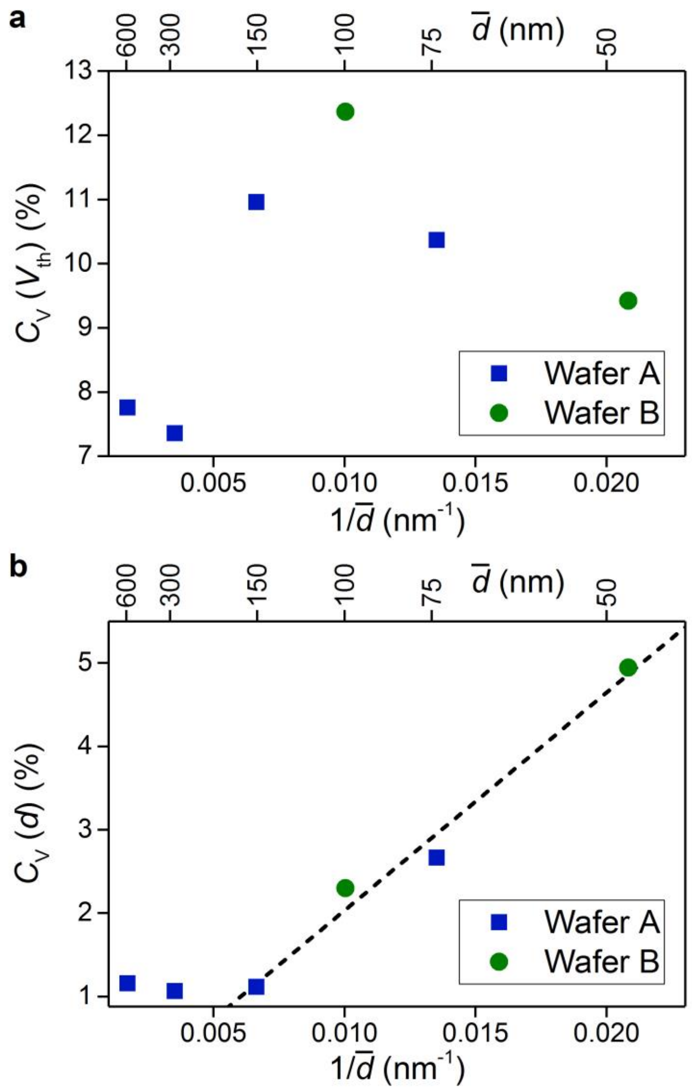

Supplementary Figure 8. Statistical dispersions in switching threshold voltage V_{th} and device size d of 1152 VO_2 nano-crossbar devices, as measured by coefficient of variation $C_V \equiv \sigma/\mu$ (ratio of the standard deviation σ to the mean μ). The device size d, as defined by the electrode linewidth, is measured by a critical dimension scanning electron microscope (CDSEM) system. **a**, C_V in switching threshold V_{th} vs. the mean device size $\bar{d}$ and its inverse $1/\bar{d}$. **b**, C_V in device size d vs. the mean device size $\bar{d}$ and its inverse $1/\bar{d}$. A total number of 1152 VO_2 devices, with six different designed dimensions, are measured from two 3-inch wafer samples (wafer A: 75×75 nm^2, 150×150 nm^2, 300×300 nm^2, and 600×600 nm^2; wafer B: 50×50 nm^2 and 100×100 nm^2). Each data point in wafer A is the result from 144 distinctive VO_2 devices with the same designed size. For each device, the force V and measure I sweeps is repeated 10 times at the same setting to obtain 20 switching events (including both positive and negative bias polarities). Each data point in wafer B is the result from 288 distinctive VO_2 devices in the same manner as in wafer A. At d < 150 nm, C_V in device size starts to increase linearly with the inverse of device size due to the impact of edge roughness in ebeam lithography. In the worst-case, $C_V(d)$ is ~5 % for 50×50 nm^2 devices. No such trend is observed in the relative variation of switching threshold, and all the $C_V(V_{th})$ data are less than 13 %.

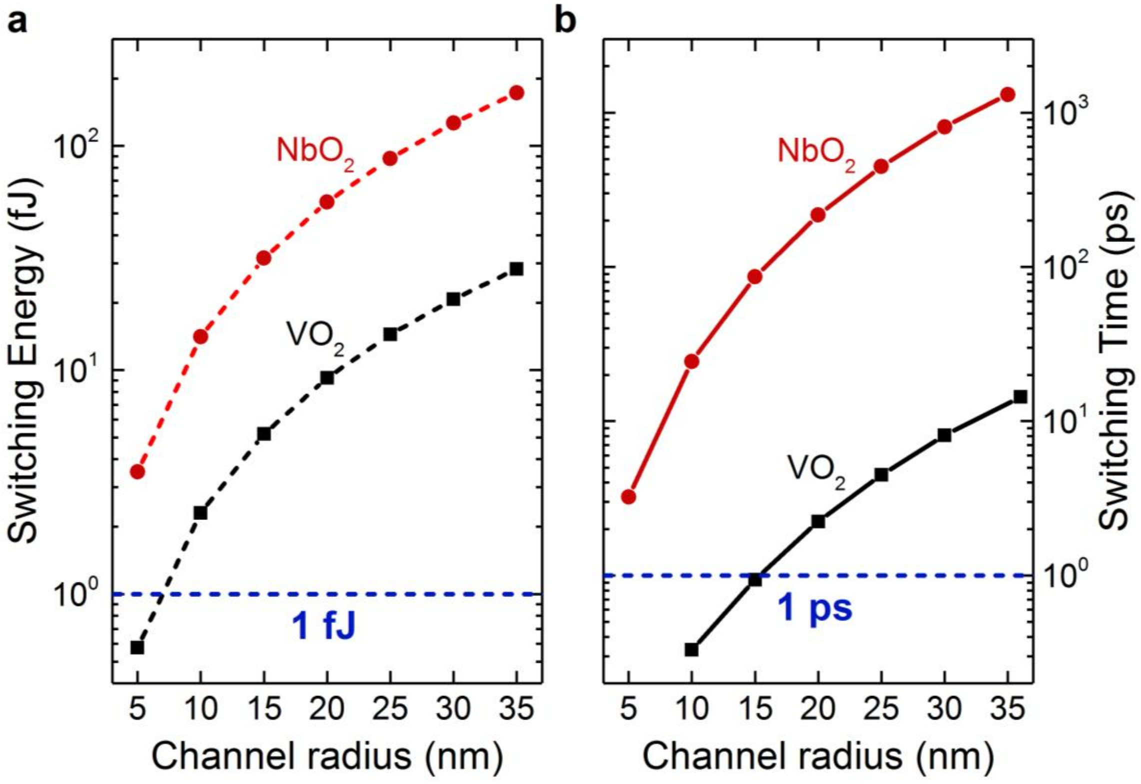

Supplementary Figure 9. Comparison of switching energy and switching time (speed) of Mott IMT in VO_2 and NbO_2 devices with channel radius of 5–35 nm. a, Calculated switching energy vs. channel radius for devices with a film thickness of 20 nm. In VO_2, due to the much smaller temperature rise needed for IMT to occur (40 K vs. 800 K), the volumetric free energy cost for IMT in VO_2 is only one-sixth of that for NbO_2 at the same crystal volume, and is less than 1 fJ at channel radii smaller than 7 nm. For details, see Supplementary Table 1. **b**, Simulated switching time vs. channel radius for devices with a film thickness of 50 nm. SPICE simulations of a VO_2-based Pearson-Anson relaxation oscillator (see the circuit in Supplementary Figure 3d) are used to estimate the switching time (speed) of Mott IMT from the rising edges of device current in each oscillation period[16]. The VO_2 channel radius is varied while all the other VO_2 model parameters are kept the same. Note that the switching speed is a material-dependent parameter, and is not affected by the values of R_L and C_1 passive components (R_L from 5 kΩ to 100 kΩ and C_1 of 22 pF were used). Simulations found that, at the same channel dimensions, Mott IMT switching in VO_2 is 100 times faster than in NbO_2, and is faster than 1 ps at channel radii smaller than 15 nm.

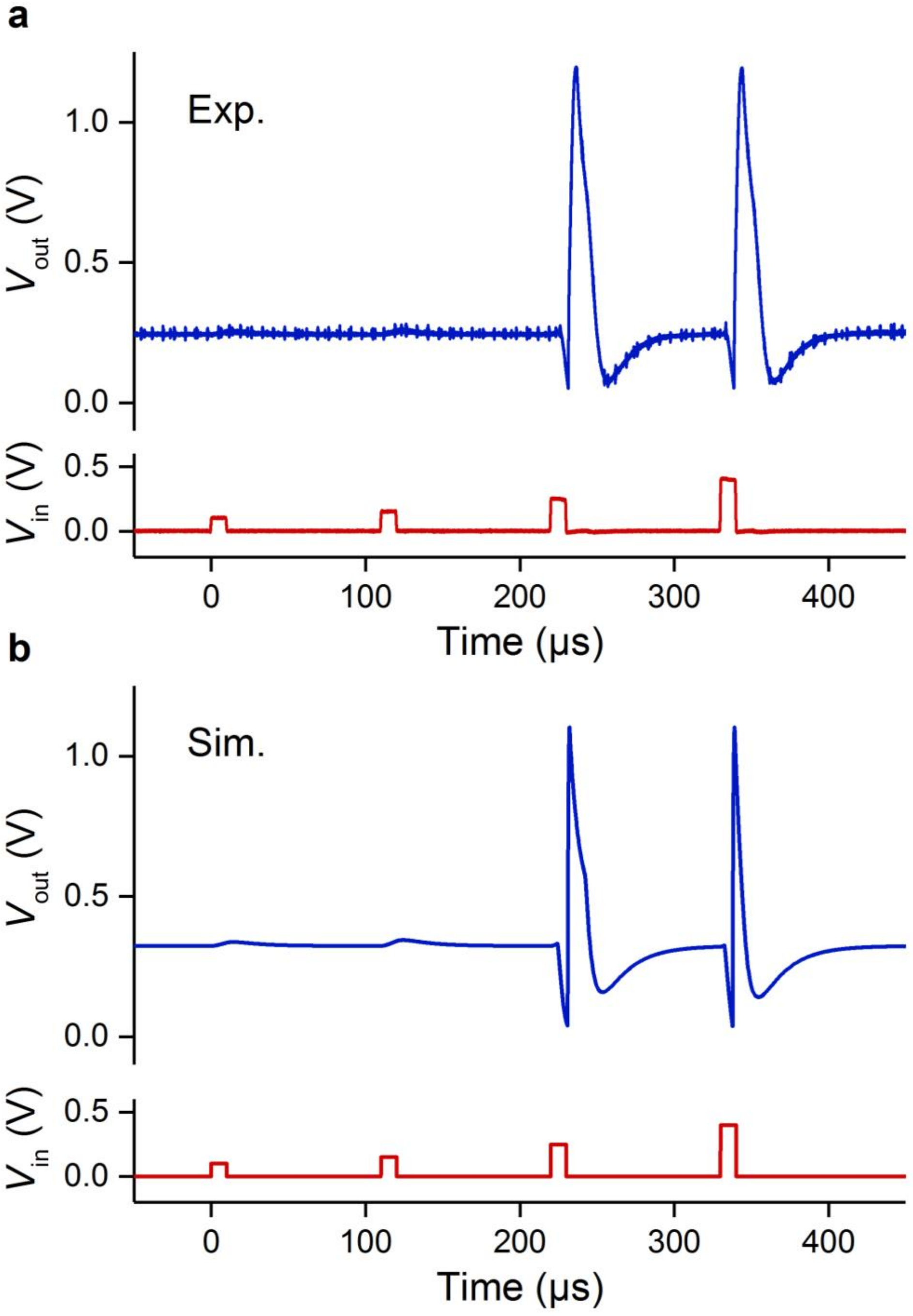

Supplementary Figure 10. All-or-nothing firing behavior in a tonic VO_2 neuron circuit. a, Experimentally measured all-or-nothing behavior, showing no response at subthreshold input voltage pulses of 0.1 V and 0.15 V, and spiking at suprathreshold input voltage pulses of 0.25 V and 0.4 V. In the suprathreshold regime, the shape or amplitude of neuron spikes does not change with an increase in the input voltage pulse amplitude. **b**, Simulated all-or-nothing behavior of the same tonic VO_2 neuron circuit. The pulse width in all the data shown is 10 µs.

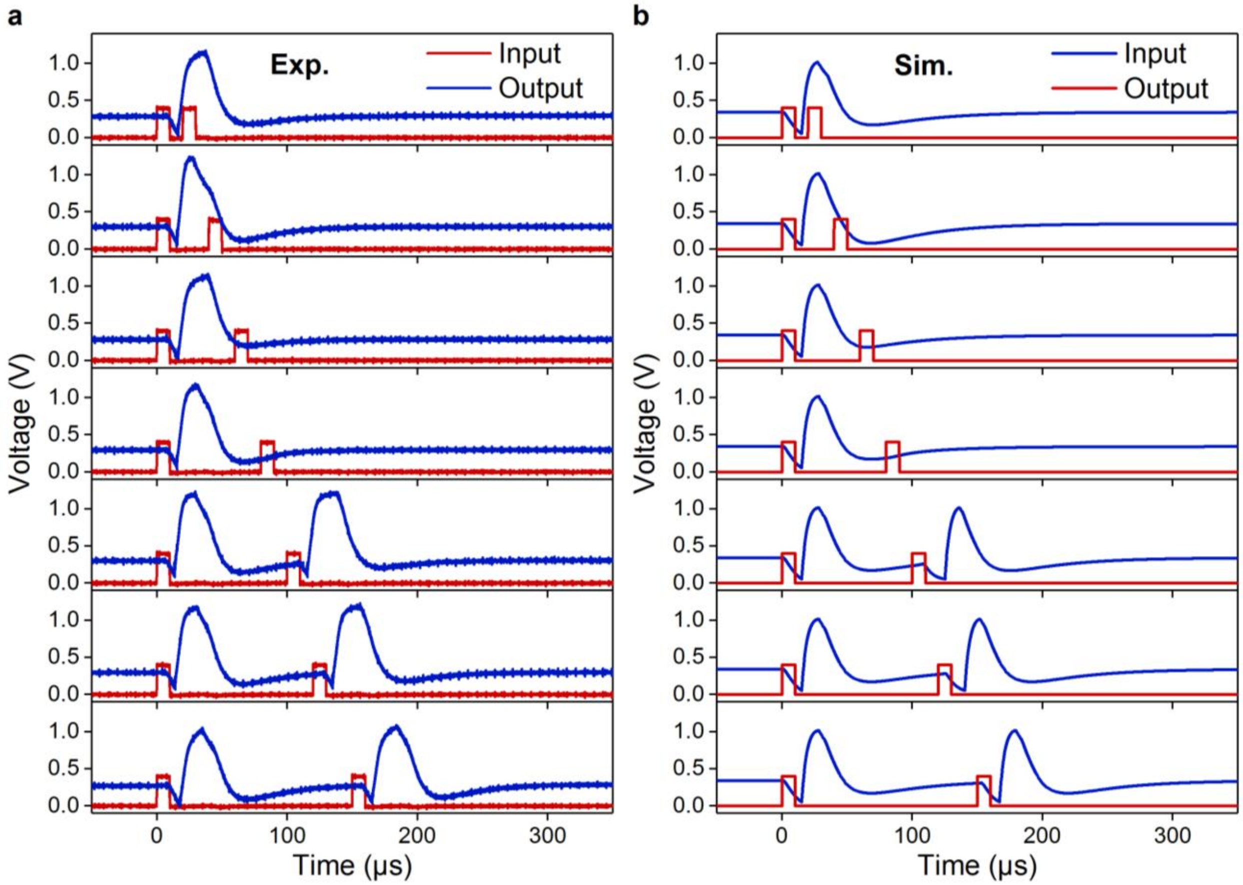

Supplementary Figure 11. Refractory period behavior in a tonic VO_2 neuron circuit. a, Experimentally measured refractory period behavior, showing a spiking in response to the first suprathreshold input voltage pulse, but no response to the second input voltage pulse if it occurred within the refractory period (panels 1 to 4 from the top). The neuron fires again if the second input voltage pulse is outside the refractory period (panels 5 to 7 from the top). From top to bottom, the periods of the input voltage pulse doublets are 20 μs, 40 μs, 60 μs, 80 μs, 100 μs, 120 μs, and 150 μs, respectively. **b**, Simulated refractory period behavior of the same tonic VO_2 neuron circuit. The pulse width in all the data shown is 10 μs.

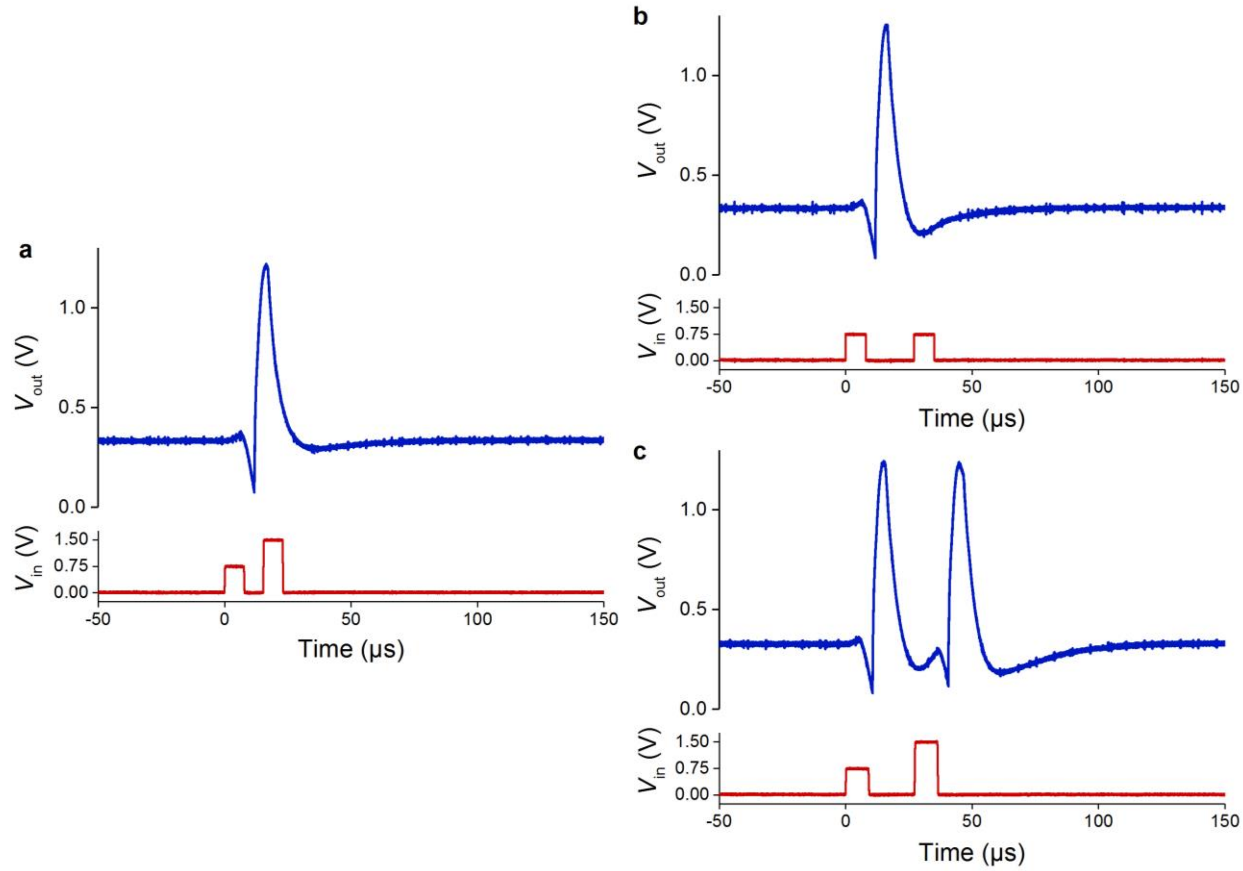

Supplementary Figure 12. Absolute and relative refractory periods experimentally observed in a tonic VO_2 neuron circuit. a, Absolute refractory period behavior, showing that if a second input voltage pulse is applied within the absolute refractory period, regardless of its strength (in this example 1.5 V was used, an amplitude greater than the spike amplitude), the neuron will never fire a second action potential in response. **b**, Relative refractory period behavior, showing that if the second input voltage pulse applied within the relative refractory period has the same strength as the first input pulse (in this example 0.75 V) , the neuron will not fire a second action potential. **c**, Relative refractory period behavior, showing that if the second input voltage pulse applied within the relative refractory period is much stronger than the first input pulse (in this example 1.5 V vs. 0.75 V) , the neuron will respond to it and fire a second action potential. The pulse width in all the data shown is 8 μs.

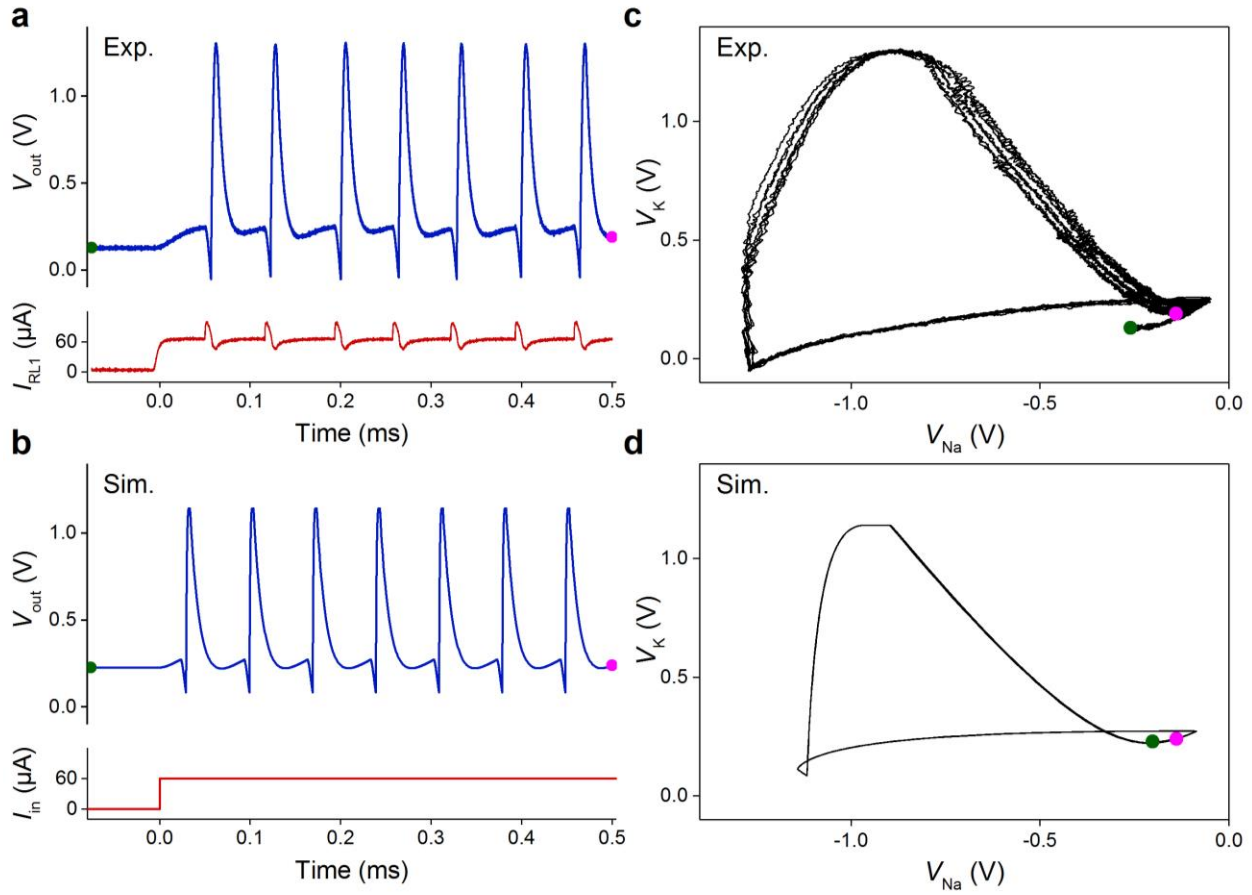

Supplementary Figure 13. Tonic spiking behavior in a tonic VO_2 neuron circuit. a, Experimentally measured tonic spiking behavior, showing that the neuron continues to fire a train of spikes in response to a d.c. input current. I_{RL1}, the current flowing through R_{L1}, was monitored by probing the voltage across it using two high-impedance (10 MΩ) oscilloscope probes. The current jitters coinciding with the output spikes are likely caused by the reflection of action potentials toward the neuron input, i.e. "back actions". **b**, Simulated tonic spiking behavior of the same tonic VO_2 neuron circuit in response to a d.c. input current. **c**, Experimental phase plane of the K^+ membrane potential V_K (aka V_{out}) vs. the Na^+ membrane potential V_{Na}. In the phase plane representation, time-domain tonic spiking turns into a limit cycle attractor. **d**, Simulated phase plane of V_K vs. V_{Na}, showing a limit cycle attractor similar to the experimental data in a qualitative manner. The green and magenta dots in **a–d** show the first and last plotted data points.

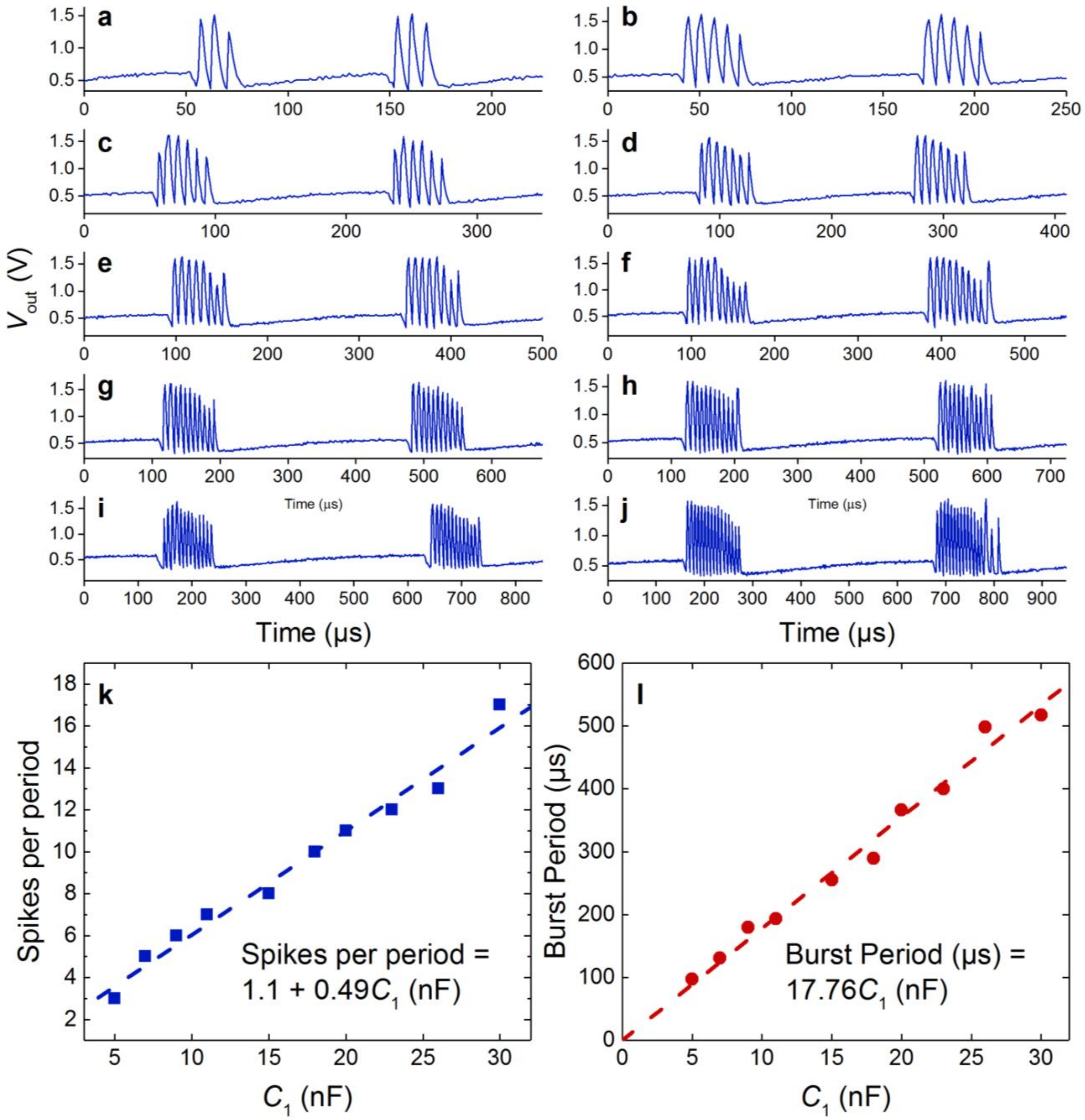

Supplementary Figure 14. Tunable tonic bursting behavior measured in a tonic VO_2 neuron circuit. a–j, Experimental tonic burst patterns measured with a fixed value of C_2 capacitor and an increasing value of C_1 capacitor ($C_1 \gg C_2$ in all the cases). As C_1 becomes larger, both the number of spikes in each burst period and the burst period itself increase. **k**, Experimental C_1 dependence of the number of spike in each burst period. Dashed line is a linear fit (with $R^2 = 0.98$) which shows a linear trend that the number of spike per period = (1.1±0.5) + (0.49±0.03)·C_1 (nF). **l**, Experimental C_1 dependence of the burst period. Dashed line is a linear fit (with $R^2 = 0.997$) which shows a linear trend that the burst period (μs) = (17.76±0.33)·C_1 (nF). In all the data shown, $C_2 \approx 1$ nF comes from the stray capacitance in the experimental setup.

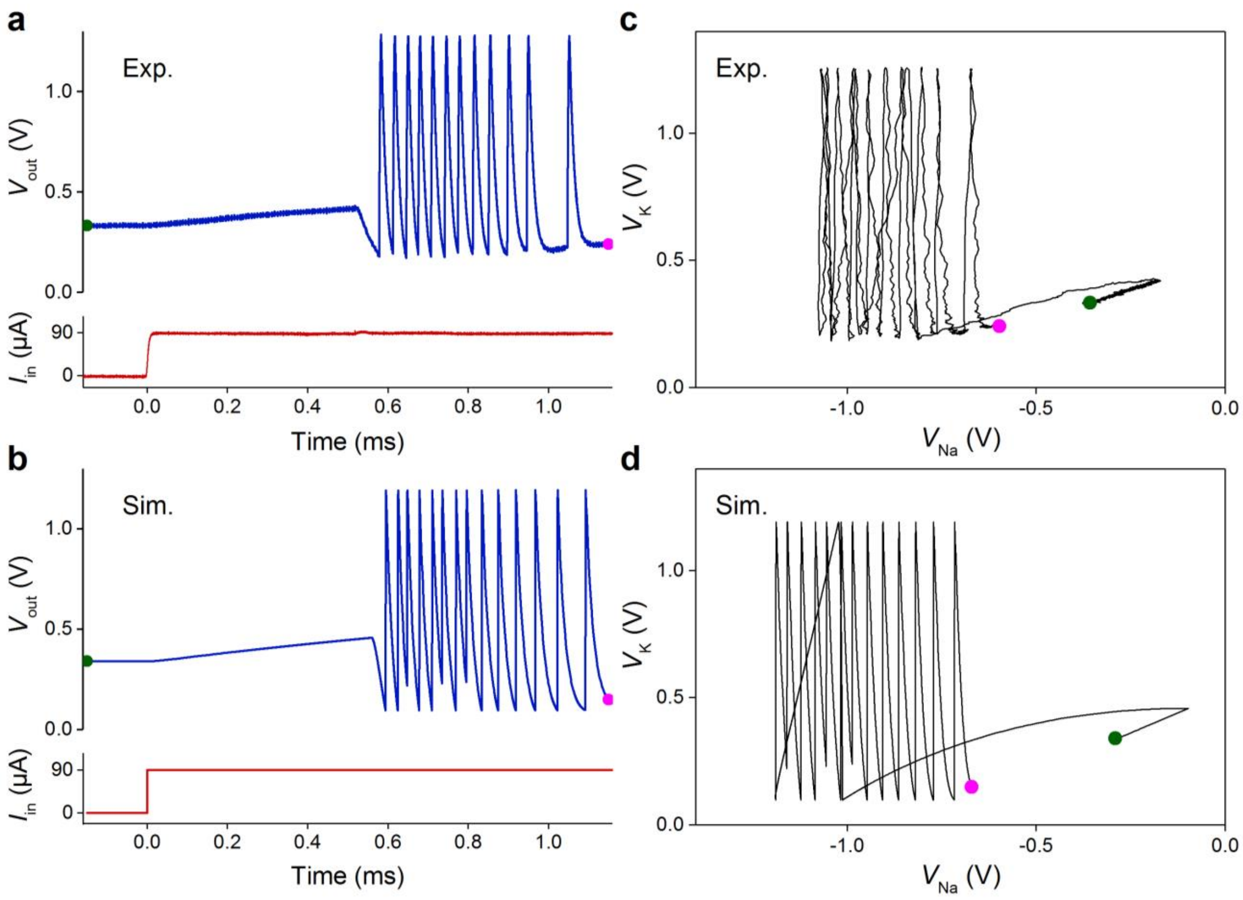

Supplementary Figure 15. Spike frequency adaptation behavior in a tonic VO_2 neuron circuit. **a**, Experimentally measured spike frequency adaptation in tonic burst under a sustained d.c. current stimulation. The spike frequency is relatively high at the onset of tonic bursting, and then it decreases over time, i.e., the neuron adapts. **b**, Simulated spike frequency adaptation behavior of the same tonic VO_2 neuron circuit in response to a d.c. input current. **c**, Experimental phase plane of the K^+ membrane potential V_K (aka V_{out}) vs. the Na^+ membrane potential V_{Na}. A 100-fold down-sampling followed by 5-point adjacent-averaging was applied to the raw oscilloscope data to smooth the curve. **d**, Simulated phase plane of V_K vs. V_{Na}, showing trajectories similar to the experimental data in a qualitative manner. The green and magenta dots in **a**–**d** show the first and last plotted data points, respectively.

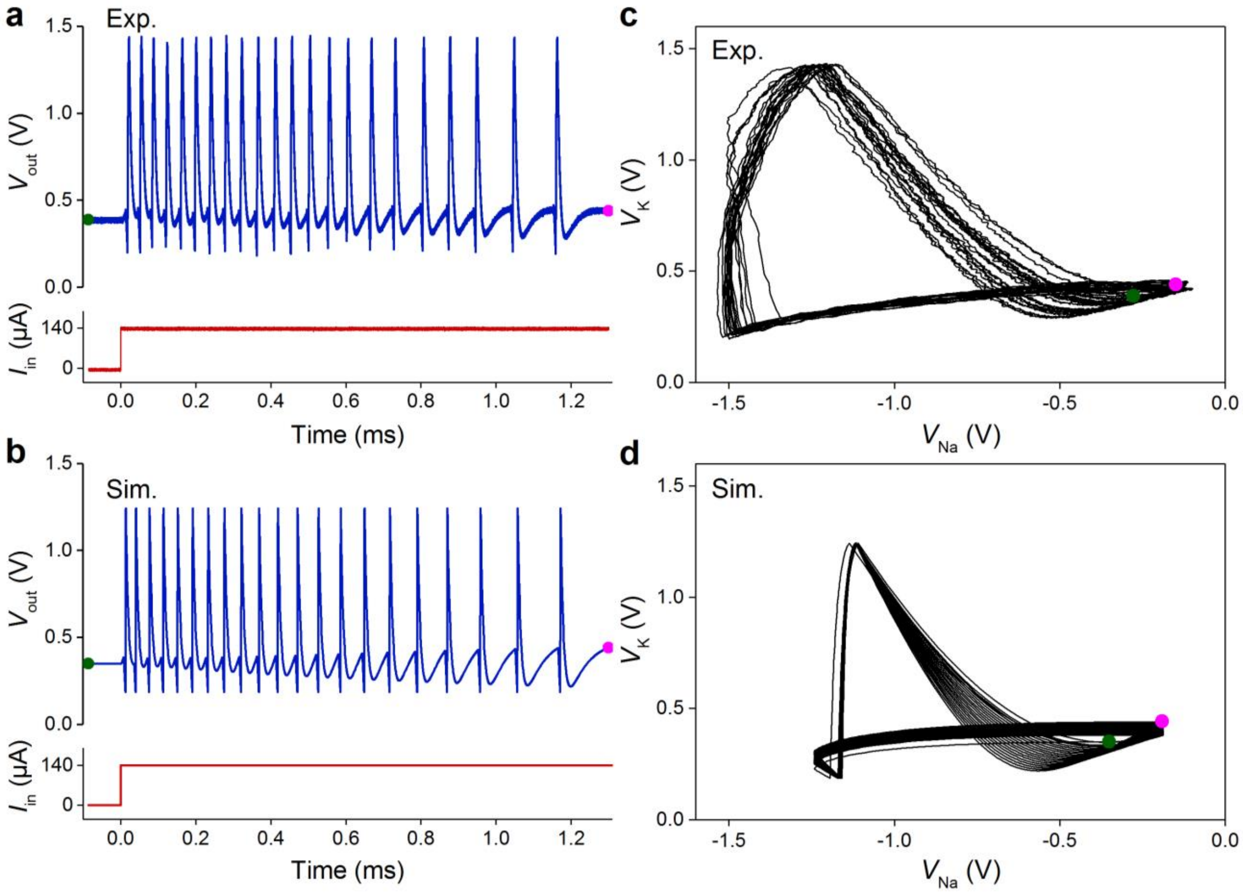

Supplementary Figure 16. Spike frequency adaptation behavior in a phasic VO_2 neuron circuit. a, Experimentally measured spike frequency adaptation in phasic burst under a sustained d.c. current stimulation. The spike frequency is relatively high at the onset of stimulation, and then it decreases over time, i.e., the neuron adapts. **b**, Simulated spike frequency adaptation behavior of the same phasic VO_2 neuron circuit in response to a d.c. input current. **c**, Experimental phase plane of the K^+ membrane potential V_K (aka V_{out}) vs. the Na^+ membrane potential V_{Na}. A 10-fold down-sampling followed by 5-point adjacent-averaging was applied to the raw oscilloscope data to smooth the curve. **d**, Simulated phase plane of V_K vs. V_{Na}, showing trajectories similar to the experimental data in a qualitative manner. The green and magenta dots in **a–d** show the first and last plotted data points.

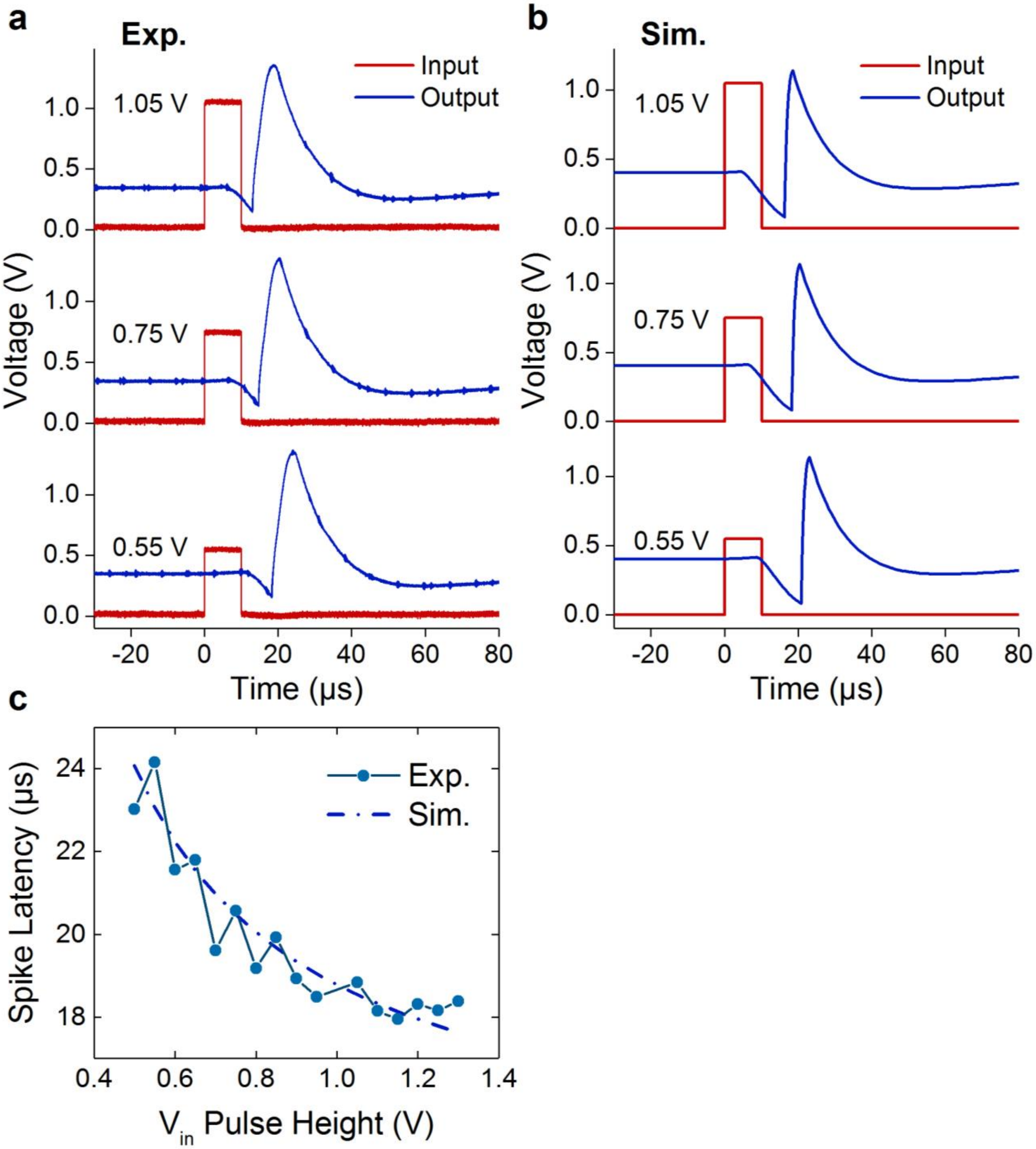

Supplementary Figure 17. Spike latency behavior in a tonic VO_2 neuron circuit. a, Experimental spike latencies in responding to suprathreshold 10 μs input voltage (V_{in}) pulses. Spike delay is longer for a relatively weak input, and it diminishes as the input gets stronger. **b**, Simulated spike latencies of the same tonic VO_2 neuron circuit. **c**, Dependences of measured and simulated spike latencies on the amplitude of the input pulse. Spike latency is arbitrarily defined as the delay between the onset of V_{in} and the peak time of spiking. Simulated spike latency τ can be fitted (with R^2 = 0.9995) by a logarithmic formula $\tau = \tau_0 + b\ln(E - V_{in})$, where τ_0 = (17.29 ± 0.02) μs, b = (3.20 ± 0.07) μs/ln(V), and E = (0.382 ± 0.007) V. The logarithmic dependence of spike latency on the input amplitude can be accounted for by the logarithmic formula of the capacitor discharge time in a relaxation oscillator[17].

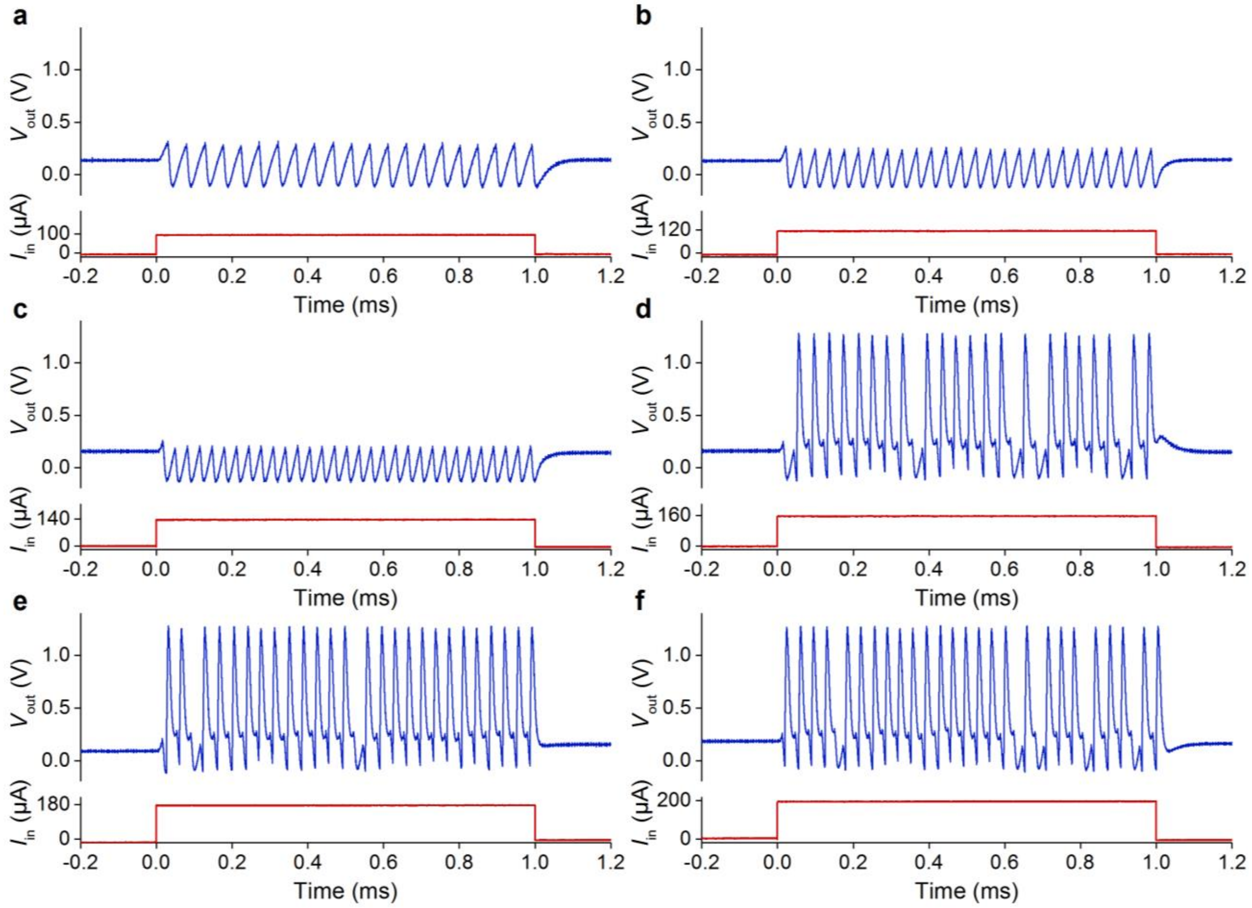

Supplementary Figure 18. Subthreshold oscillation behavior measured in a tonic Class 2 VO_2 neuron circuit. a–c, Subthreshold oscillations in the neuron output, i.e., the K^+ channel membrane potential, under a sustained d.c. current stimulation of 100 µA, 120 µA, and 140 µA, respectively. It is evident that the frequency of the oscillations increases with the input current level. **d–f**, Tonic spiking intermixed with occasional subthreshold oscillations (as demonstrated by the missing spikes) in the neuron output under a sustained d.c. current stimulation of 160 µA, 180 µA, and 200 µA, respectively. The transition from subthreshold oscillations to tonic spiking is better observed by ramping up the current stimulation. See Figure 4b in the main text for details.

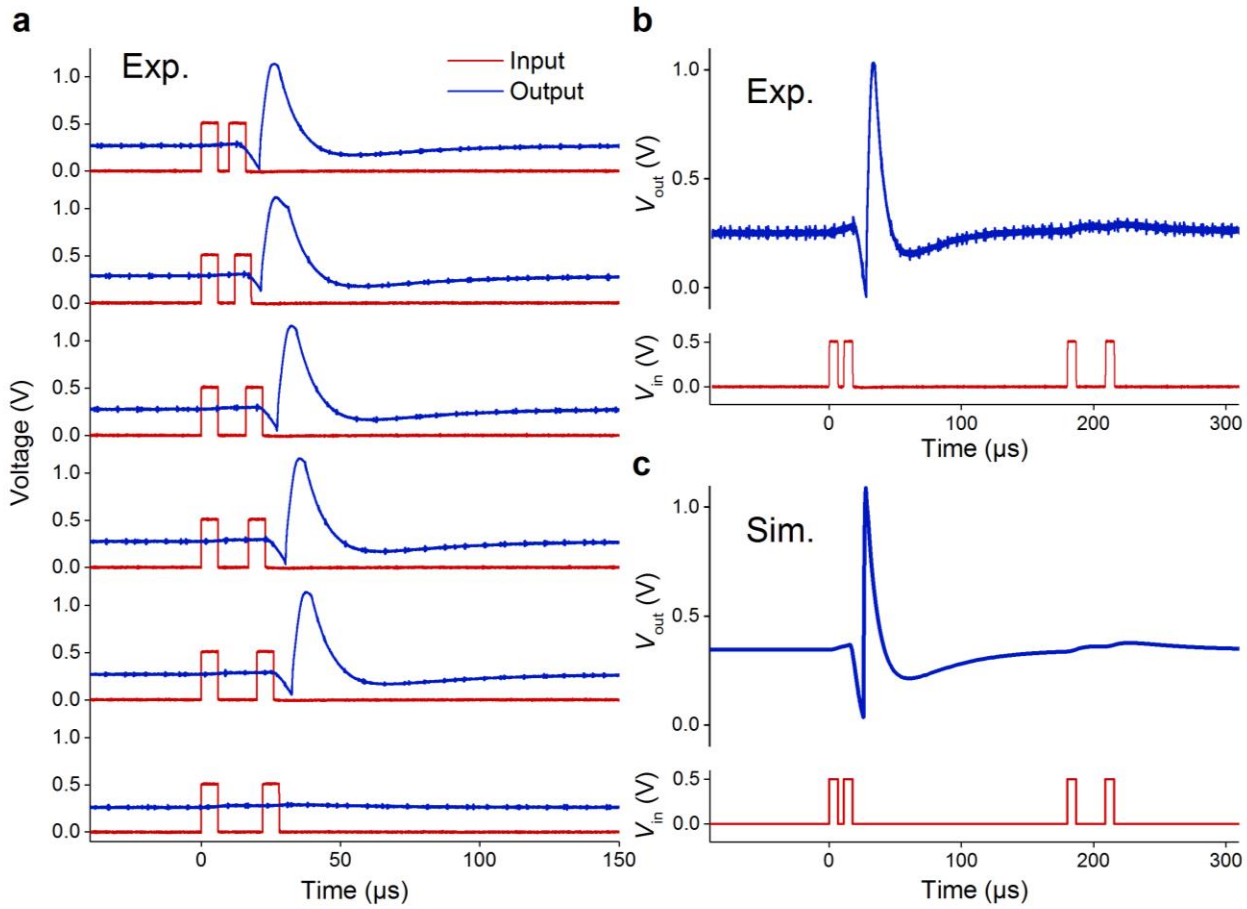

Supplementary Figure 19. Integrator behavior in a tonic Class 1 VO_2 neuron circuit. a, Experimentally measured integrator behavior, showing that the neuron fires an action potential if a doublet of two subthreshold input voltage pulses are applied with sufficiently short interval between them The pulse interval for data in panel 1–5 starting from the top is 4 μs, 6 μs, 10 μs, 11 μs, and 14 μs, respectively. The neuron does not spike if the two subthreshold input voltage pulses are too far apart from each other, i.e., the data in the bottom panel, with an interval of 16 μs. **b**, Experimental integrator behavior of the same neuron circuit as in **a**, demonstrated by applying two doublets of subthreshold input voltage pulses in the same measurement. The neuron fires a spike in response to the first input pulse doublet with a shorter interval (5 μs), but does not fire in response to the second input pulse doublet with a longer interval (23 μs). **c**, Simulated integrator behavior of the same neuron circuit as in **b**. The pulse width and amplitude in all the data shown is 6 μs and 0.5 V, respectively.

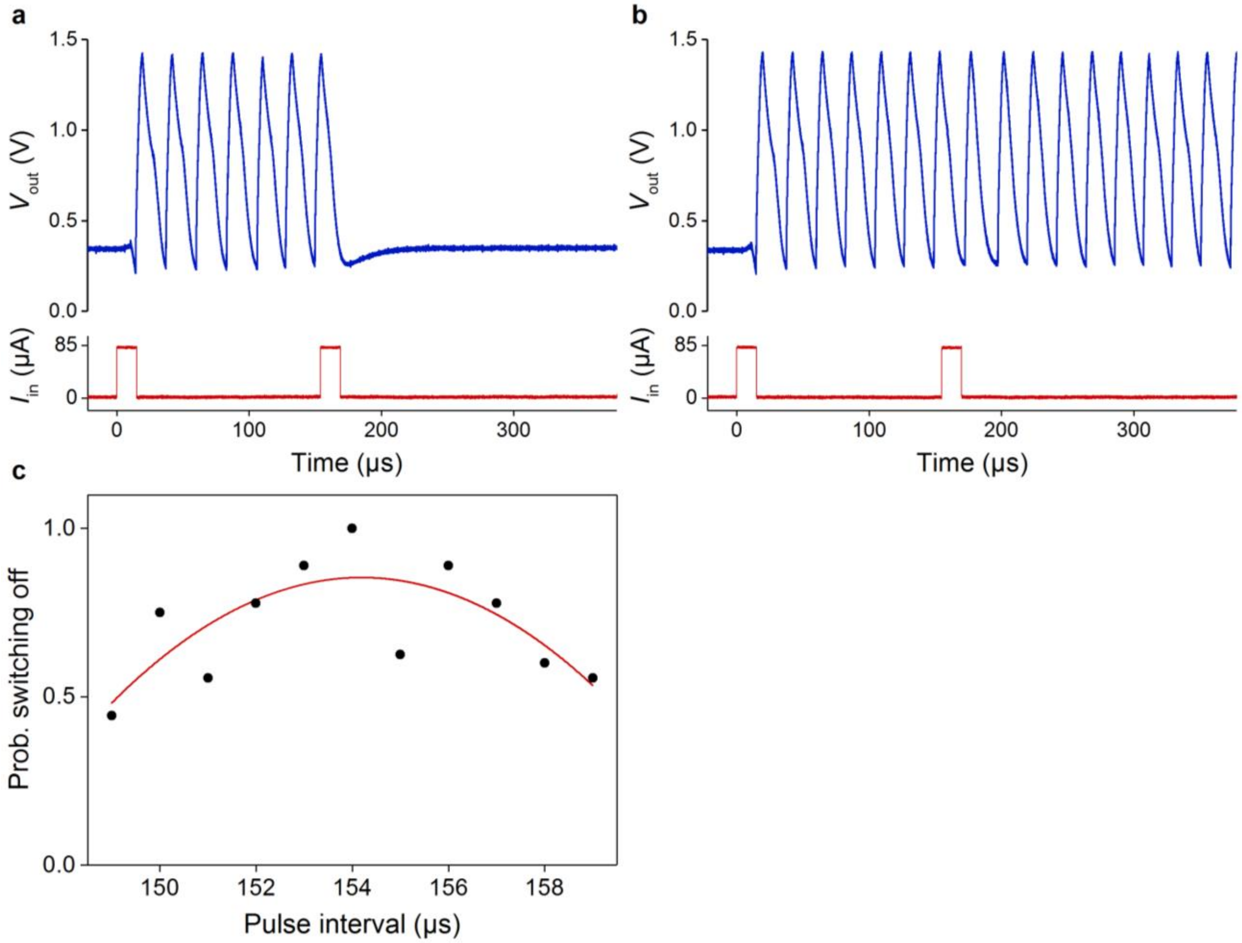

Supplementary Figure 20. Bistability behavior measured in a tonic VO_2 neuron circuit. a, The neuron is driven from the resting mode into a persistent tonic spiking mode (a self-oscillation) by applying the first input pulse stimulation. A second input pulse arriving at an interval of 154 µs successfully switches the neuron from tonic spiking back to the resting mode. **b**, A second input pulse arriving at an interval of 155 µs fails to switch the neuron from tonic spiking back to the resting mode. In the measurements, 0.85 V and 15 µs wide voltage pulses sent from an arbitrary waveform generator (AWG) were converted into 85 µA input current pulses (I_{in}) using a stimulus isolator with a gain of 0.1 mA V^{-1}. Input current was not monitored because the load resistor R_{L1} was set to be zero to enable bistability. The plotted I_{in} waveforms are calculated from the monitored AWG voltage waveforms. **c**, Probability (success rate) of the second input pulse switching off the self-oscillation vs. the pulse interval. Red line is a second-order polynomial fit. Each data point represents the statistics from 8 to 10 such attempts. At an interval of 154 µs, the success rate is 100 %, or the neuron self-oscillation was switched off in 10 out of 10 attempts. At an interval of 155 µs, the success rate dropped to 62.5 %, or 5 out of 8 attempts. Despite the scattering of data points, it is evident that the success rate peaks at around 154 µs interval, and it drops off as the interval is detuned away. This observation is consistent with the interpretation that the input must arrive at an appropriate phase of oscillation for it to switch the neuron from tonic spiking back to the resting mode. Large scattering in the success rates may be explained by the stochastic onset of tonic spiking, which shifts the phase of oscillation randomly with respect to the fixed intervals used in measurements.

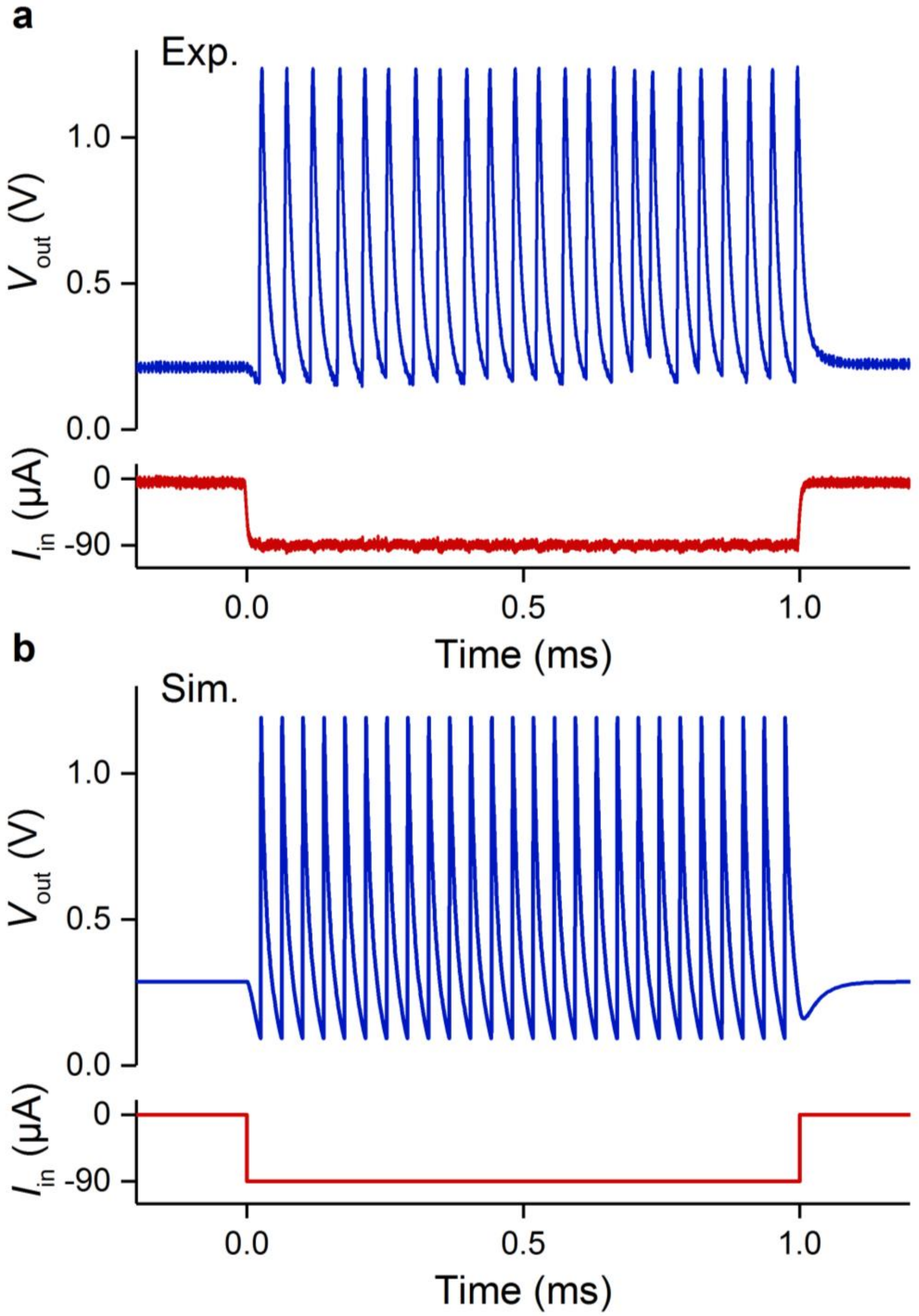

Supplementary Figure 21. Inhibition-induced spiking (IIS) behavior in a tonic VO_2 neuron circuit. a, Experimentally measured inhibition-induced spiking behavior, showing that the neuron is quiescent (at rest) when there is no input current, but fires a tonic spike train when it is hyperpolarized by an inhibitory (negative) input current of -90 µA. **b**, Simulated inhibition-induced spiking behavior of the same tonic VO_2 neuron circuit. In biology, many thalamo-cortical neurons exhibit the IIS feature. The mechanism was attributed to inhibitory-input induced activation of the *h*-current and deactivation of the Ca^{2+} *T*-current[18].

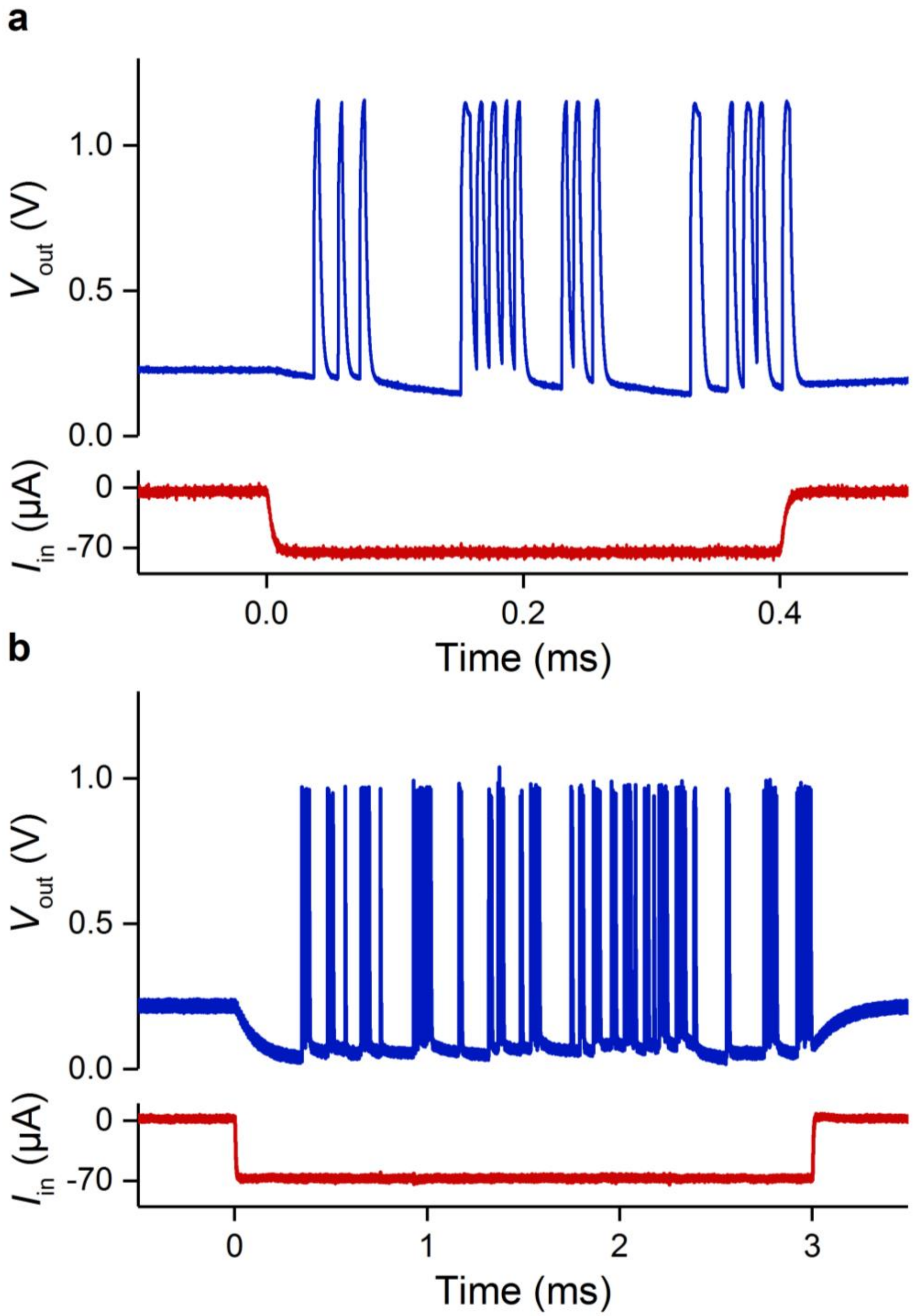

Supplementary Figure 22. Experimental inhibition-induced bursting (IIB) behaviors in tonic VO_2 neuron circuits. The neuron is quiescent (at rest) when there is no input current, but fires irregular bursts of spikes when it is hyperpolarized by an inhibitory (negative) input current of -70 µA. Similar to the case of tonic bursting induced by excitatory (positive) inputs, IIB requires a much slower Na^+ channel than the K^+ channel, or $C_1 \gg C_2$. **a**, IIB measured from a tonic VO_2 neuron circuit with the discrete membrane capacitors set at C_1 = 35 nF and C_2 = 0 nF. **b**, IIB measured from another tonic VO_2 neuron circuit with C_1 = 21 nF and C_2 = 0 nF. In the test setup, for each discrete capacitor there also exists a stray capacitance of ~1 nF, mostly contributed by the cables. Other circuit parameters can be found in Supplementary Table 2.

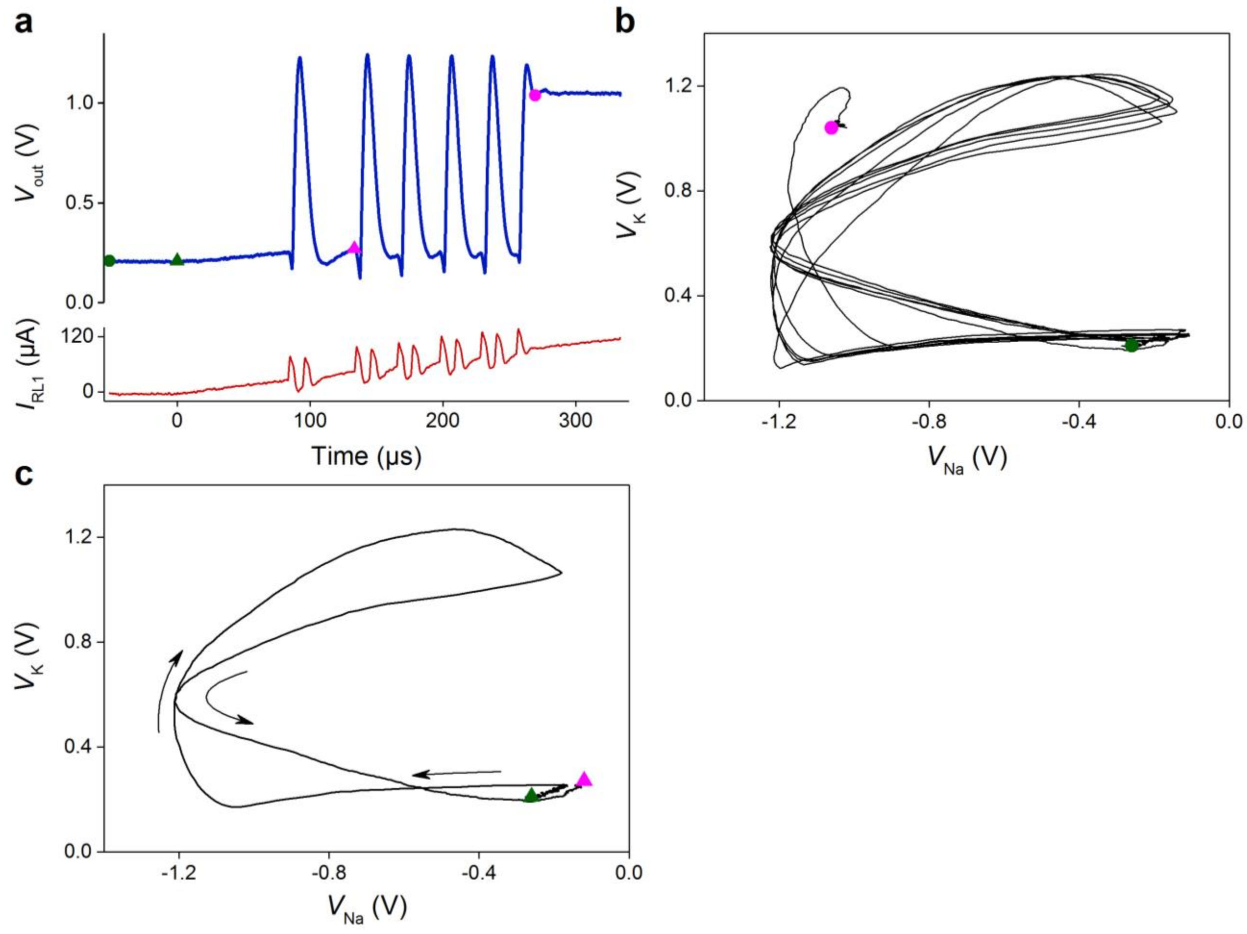

Supplementary Figure 23. Experimental excitation block behavior in a Class 2 tonic VO_2 neuron circuit. a, Experimentally measured excitation block behavior, showing that the neuron fires tonic spikes when the input current ramps above the spiking threshold at 25 µA. As the stimulus current further increases beyond 95 µA, the neuron suddenly ceases to spike and the output is locked to an elevated value (1.05 V). **b**, Experimental phase plane of the K^+ membrane potential V_K (aka V_{out}) vs. the Na^+ membrane potential V_{Na} for the data shown in **a**. The green and magenta dots in **a** and **b** show the onset of current ramp and the onset of excitation block. The V_K–V_{Na} trajectory shows characteristics of a distorted letter 'B'-shaped limit cycle attractor. Each loop of the trajectory corresponds to a complete cycle of action potential generation in **a**. Further increase of the stimulus current beyond 95 µA causes the disappearance of the limit cycle attractor and the locking of neuron state (the magenta dot). A 100-fold down-sampling followed by 16-point adjacent-averaging was applied to the raw oscilloscope data to smooth the curve. **c**, Experimental phase plane of V_K vs. V_{Na} for the time duration of the first tonic spike in **a** (marked by green and magenta triangles). The loop of letter 'B'-shaped trajectory is marked with arrows. In theory, excitation block is attributed to the conversion from a spiking limit cycle to a supercritical Andronov-Hopf bifurcation phenomenon and is explained in FitzHugh-Nagumo model by the phase plane approach, which shows a stimulus-induced shift of the equilibrium through stable-unstable-stable branches of the 'N'-shaped nullcline[19].

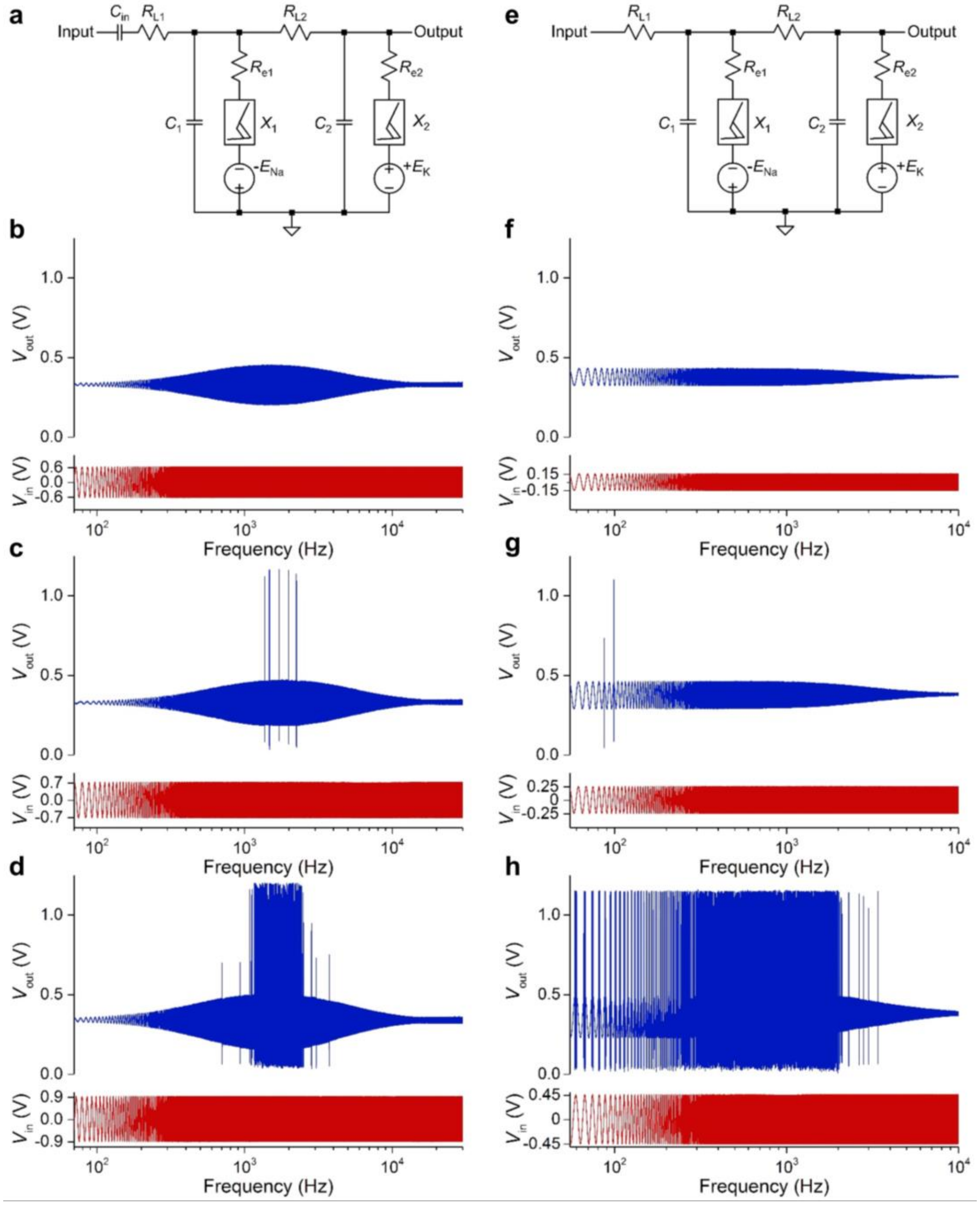

Supplementary Figure 24. Experimental resonator behavior in a phasic VO_2 neuron (a) as compared with integrator behavior in a tonic VO_2 neuron (b). a, The phasic neuron circuit with a capacitively-coupled input. **b**, Oscilloscope-captured phasic neuron response to a subthreshold (0.6 V) frequency-sweeping sinusoidal voltage input (ZAP sweep[20]), showing a pass band centered around ~17 kHz. **c**, Sporadic spikes occur near ~17 kHz as the ZAP sweep amplitude increased to 0.7 V. **d**, Intensified spiking near ~17 kHz as the ZAP sweep amplitude further increased to 0.9 V. **e**, The tonic neuron circuit with all the circuit elements the same as the phasic neuron in **a**, except for the missing C_{in} capacitor. **f–g**, Frequency-domain response of the tonic neuron to ZAP sweeps with an amplitude of 0.6 V, 0.7 V, 0.9 V, respectively. The integrator nature is reflected by the low-pass filter characteristics of the neuron response.

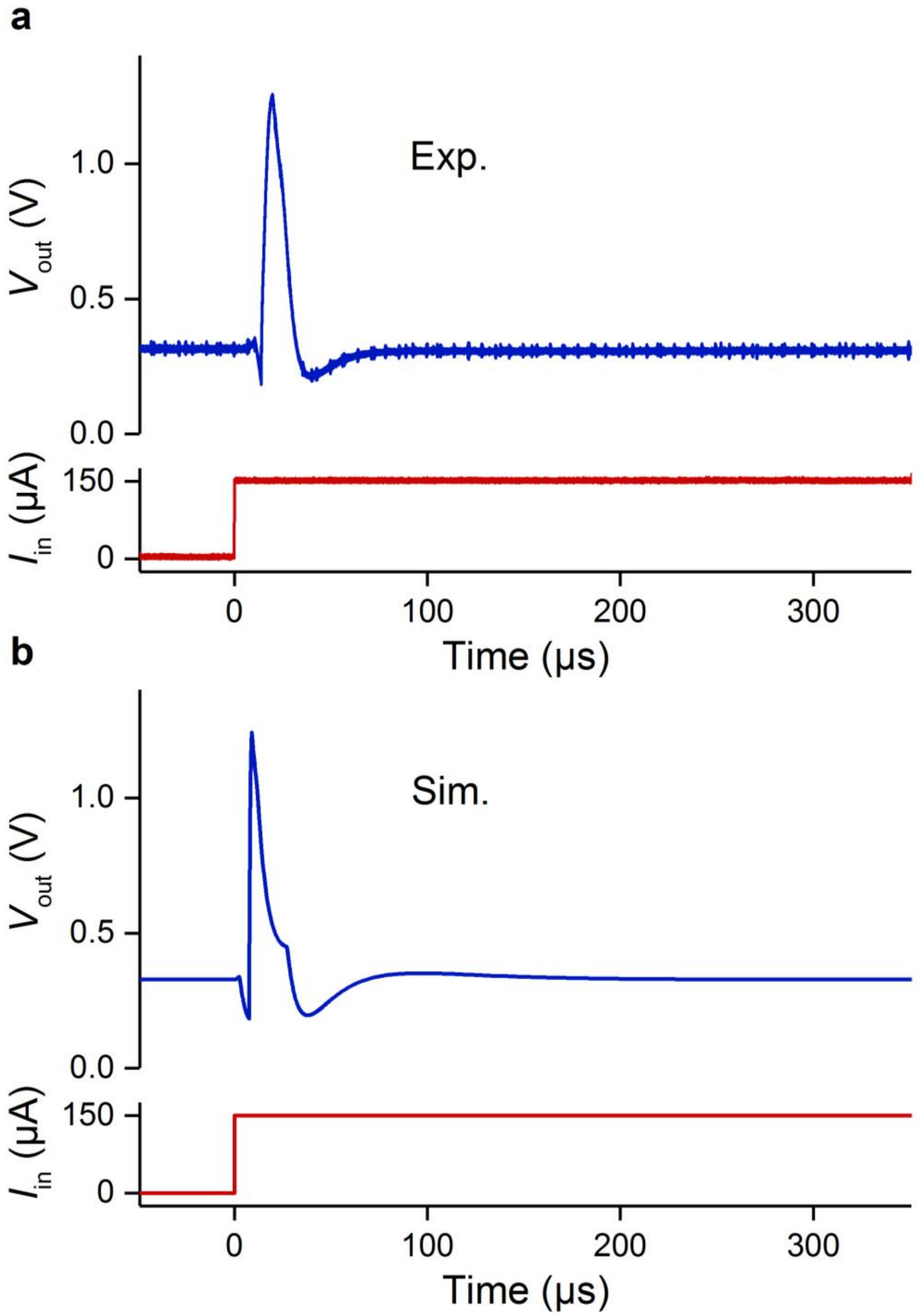

Supplementary Figure 25. Phasic spiking (Class 3 excitable) behavior in a phasic VO_2 neuron circuit. a, Experimentally measured phasic spiking behavior, showing that the neuron fires only a single spike at the onset of a d.c. input current, and then it remains quiescent even in the presence of the input current. The plotted input current (I_{in}) waveform is calculated from the monitored AWG voltage waveform, because in the phasic neuron circuit the load resistor R_{L1} is replaced with a capacitor C_{in}. **b**, Simulated phasic spiking behavior of the same phasic VO_2 neuron circuit.

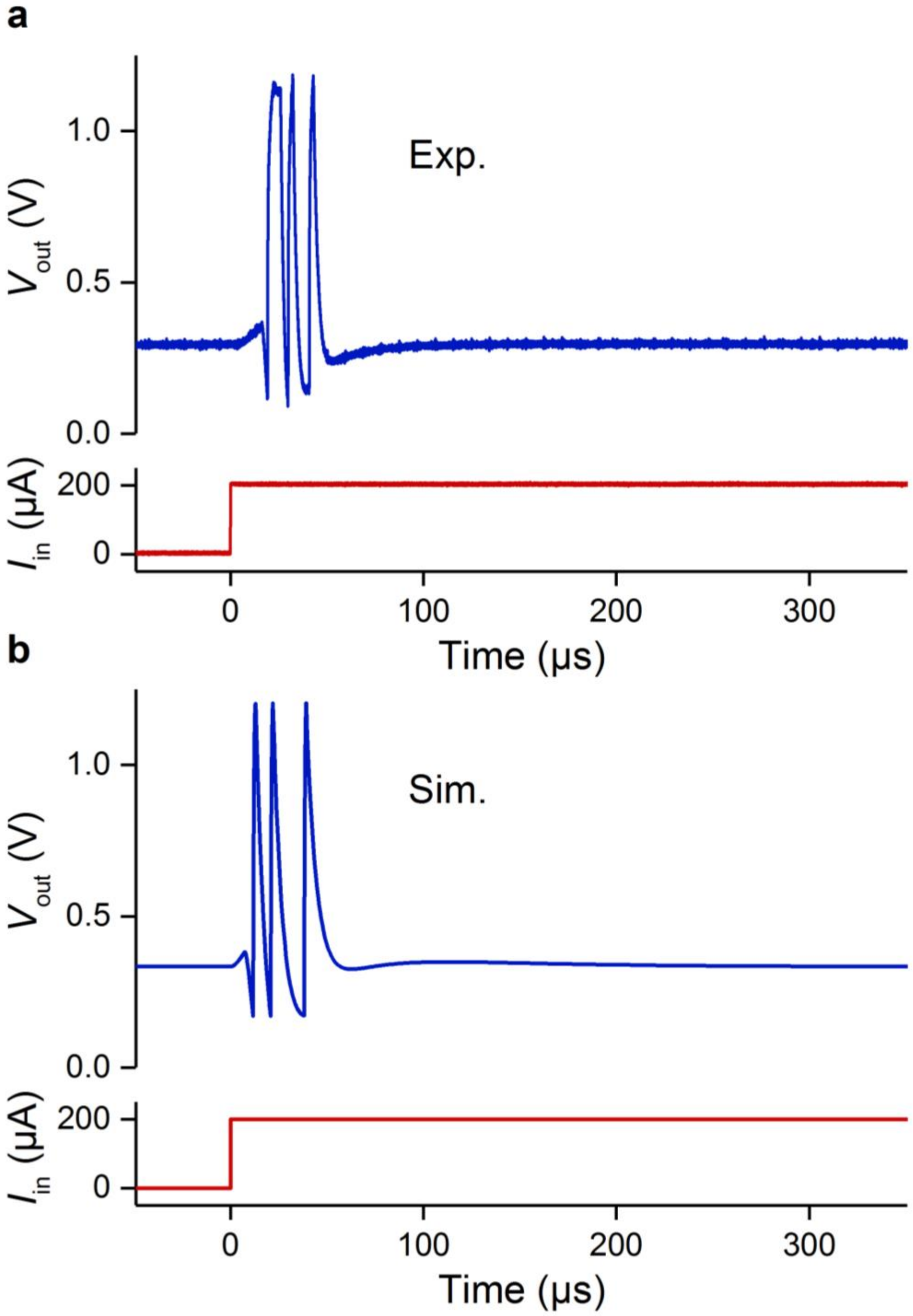

Supplementary Figure 26. Phasic bursting behavior in a phasic VO_2 neuron circuit. a, Experimentally measured phasic bursting behavior, showing that the neuron fires only a single period of burst spikes at the onset of a d.c. input current, and then it remains quiescent even in the presence of the input current. The plotted input current (I_{in}) waveform is calculated from the monitored AWG voltage waveform, because the load resistor R_{L1} is replaced with a capacitor C_{in} in the phasic neuron circuit. **b**, Simulated phasic bursting behavior of the same phasic VO_2 neuron circuit.

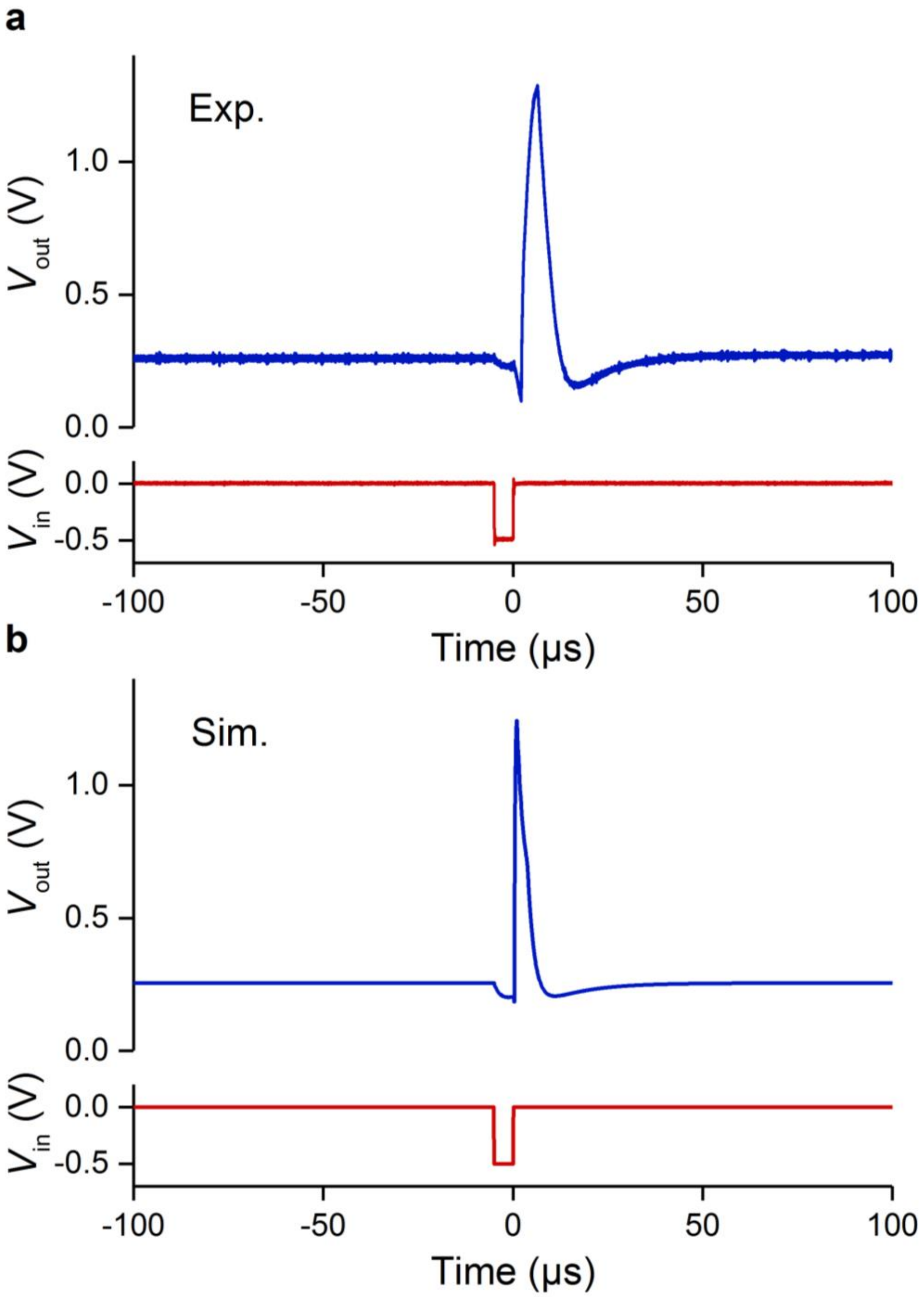

Supplementary Figure 27. Rebound spike behavior in a phasic VO_2 neuron circuit. a, Experimentally measured rebound spike behavior, showing that when the neuron receives and then is released from an inhibitory (negative) input, it fires a post-inhibitory (rebound) spike, in response to the release (the rise edge) of the inhibitory input waveform). **b**, Simulated rebound spike behavior of the same phasic VO_2 neuron circuit. In the case of excitatory input, a phasic spike is fired at the rise edge of the input current (see Supplementary Fig. 25). In the case of inhibitory input, a rebound spike is fired also at the rise edge of the input current. Equipped with a capacitively-coupled input, the phasic neuron essentially acts as a rise-edge detector.

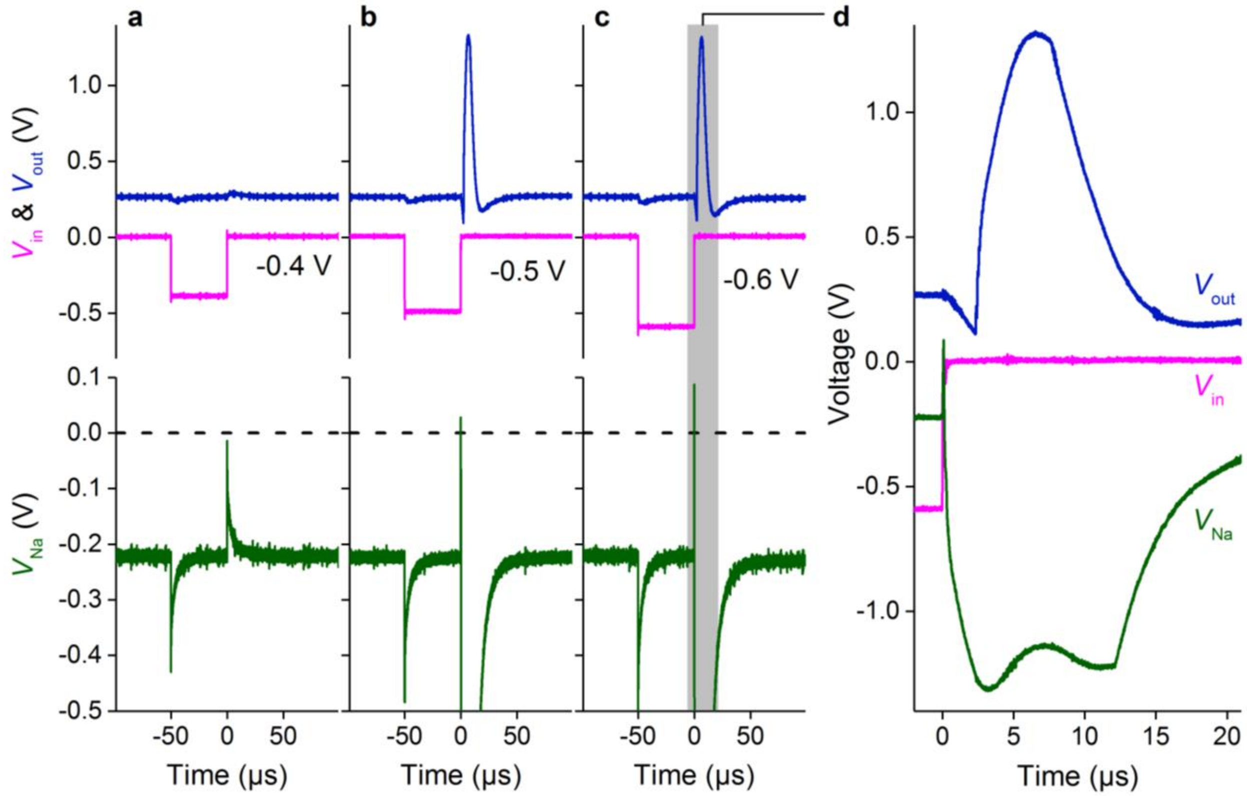

Supplementary Figure 28. Experimental all-or-nothing characteristics in the rebound spike behavior of a phasic VO_2 neuron circuit. Similar to the all-or-nothing response to an excitatory stimulus, for an inhibitory input, there also exists a threshold in its amplitude for a rebound spike to be fired when the input is released. **a**, The lack of response to a subthreshold inhibitory input pulse (-0.4 V), which is not strong enough for the neuron to fire a rebound spike at the rise edge of the input. It is noticed that the Na^+ channel membrane potential surges up at the rise edge of the input, but it still stays below zero and therefore does not trigger the Na^+ channel to open. **b–c**, A rebound spike is fired in response to a suprathreshold inhibitory input pulse of -0.5 V and -0.6 V, respectively. In both cases, the Na^+ channel membrane potential surges above zero at the rise edge of the input, triggering the coordinated opening or closing of the Na^+ and K^+ channels and an action potential generation. **d**, a close-up view of the greyed-out area in **c**, showing more details in the time evolution of the input V_{in}, the Na^+ channel membrane potential V_{Na}, and the K^+ channel membrane potential (the neuron output) V_{out}.

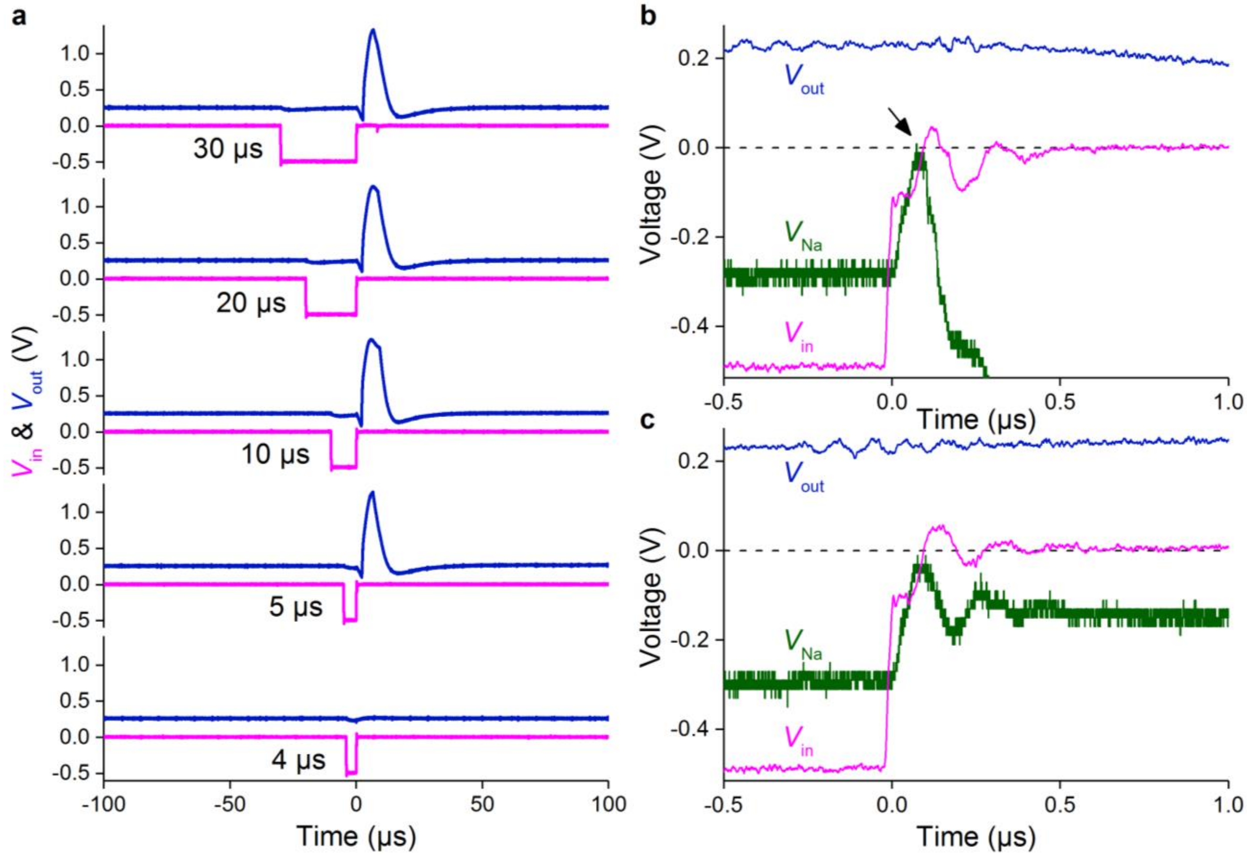

Supplementary Figure 29. Experimental input-duration threshold characteristics in the rebound spike behavior of a phasic VO_2 neuron circuit. a, From top to bottom, a rebound spike is fired in response to a suprathreshold inhibitory input pulse of -0.5 V with a duration of 30 μs, 20 μs, 10 μs, and 5 μs, respectively, but the neuron does not fire if the duration is further shortened to 4 μs (the bottom panel). **b**, a close-up view of the case for 5 μs inhibitory input in **a**, showing that the Na^+ channel membrane potential V_{Na} surges above zero at the rise edge of the input (arrow), triggering a rebound spike. **c**, a close-up view of the case for 4 μs inhibitory input in **a**, showing that the Na^+ channel membrane potential V_{Na} surges but stays below zero at the rise edge of the input, and is thus incapable of triggering the rebound spike.

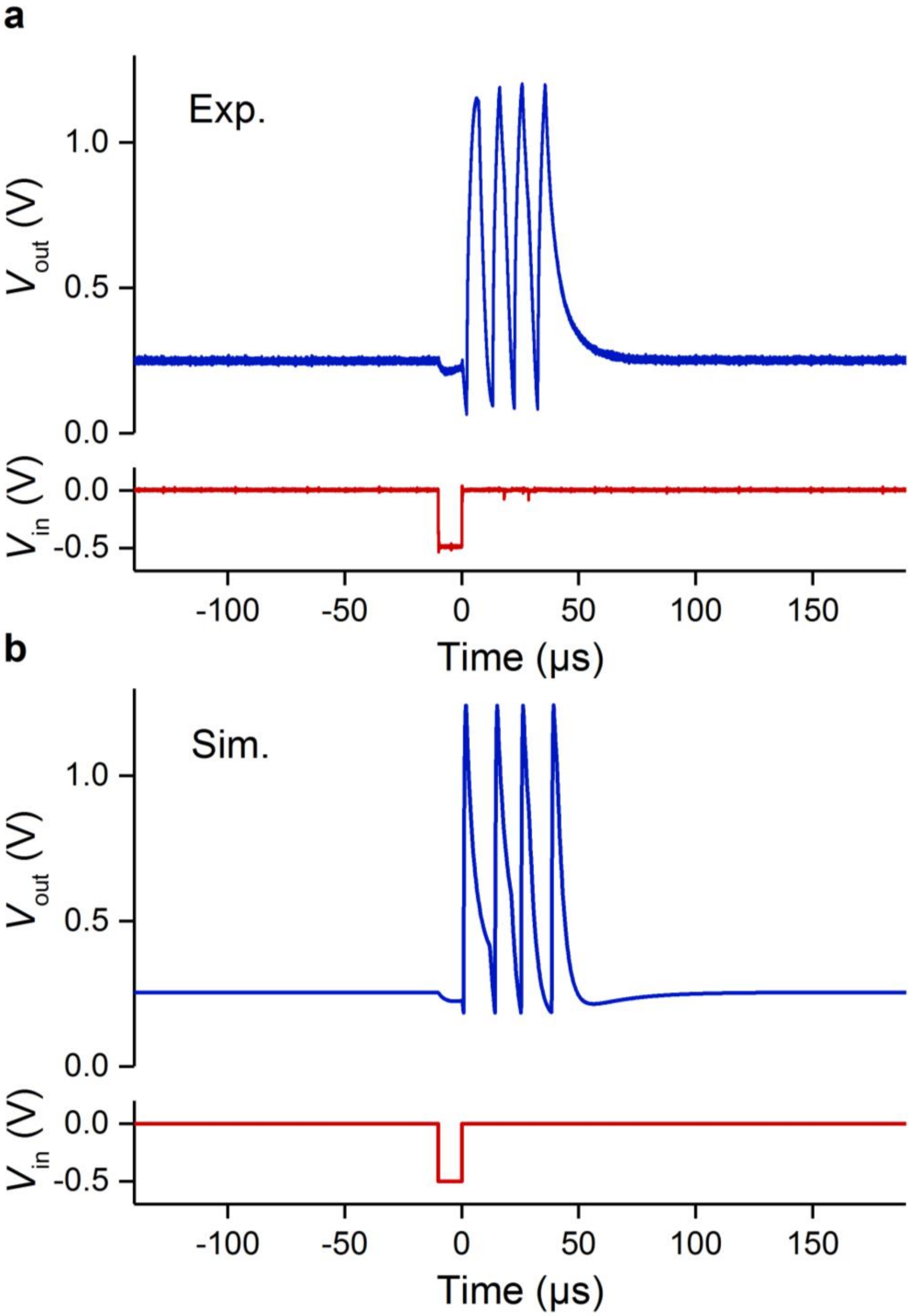

Supplementary Figure 30. Rebound burst behavior in a phasic VO_2 neuron circuit. a, Experimentally measured rebound burst behavior, showing that when the neuron receives and then is released from an inhibitory (negative) input, it fires a post-inhibitory (rebound) burst of spikes, in response to the release (the rise edge) of the inhibitory input waveform). **b**, Simulated rebound burst behavior of the same phasic VO_2 neuron circuit.

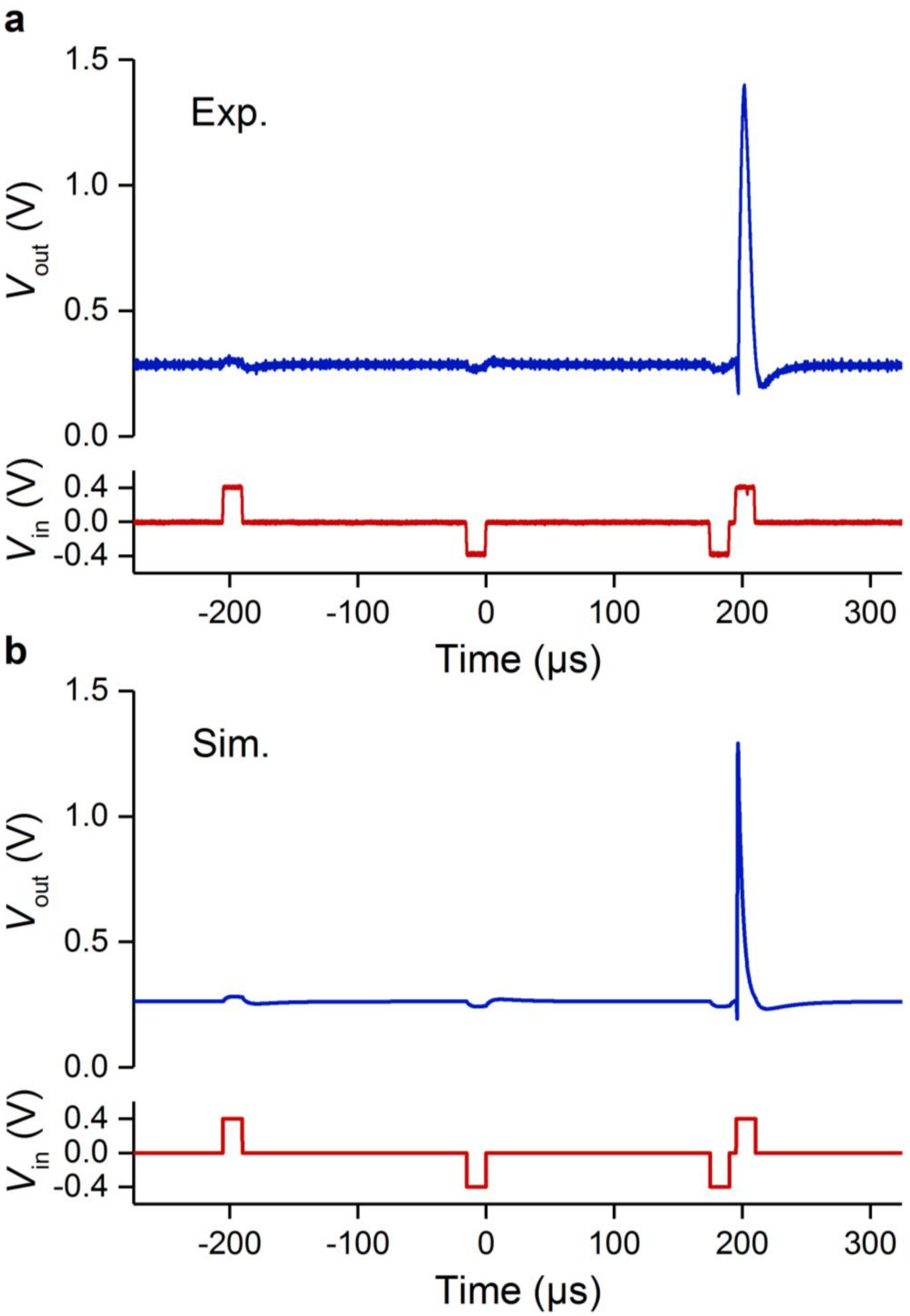

Supplementary Figure 31. Threshold variability behavior in a phasic VO_2 neuron circuit. a, Experimental threshold variability, showing that the neuron does not fire when it receives a brief subthreshold excitatory or inhibitory input pulse, but it fires a spike if the inhibitory input pulse is followed by an excitatory pulse (both are subthreshold) as long as the interval is short enough. The preceding inhibitory pulse lowers the threshold and makes the neuron more excitable. **b**, Simulated threshold variability of the same phasic VO_2 neuron circuit. Combining the rise edges of the inhibitory and excitatory pulses into one effective rise edge, threshold variability is caused by the same mechanism as the threshold seen in rebound spike (See Supplementary Fig. 28).

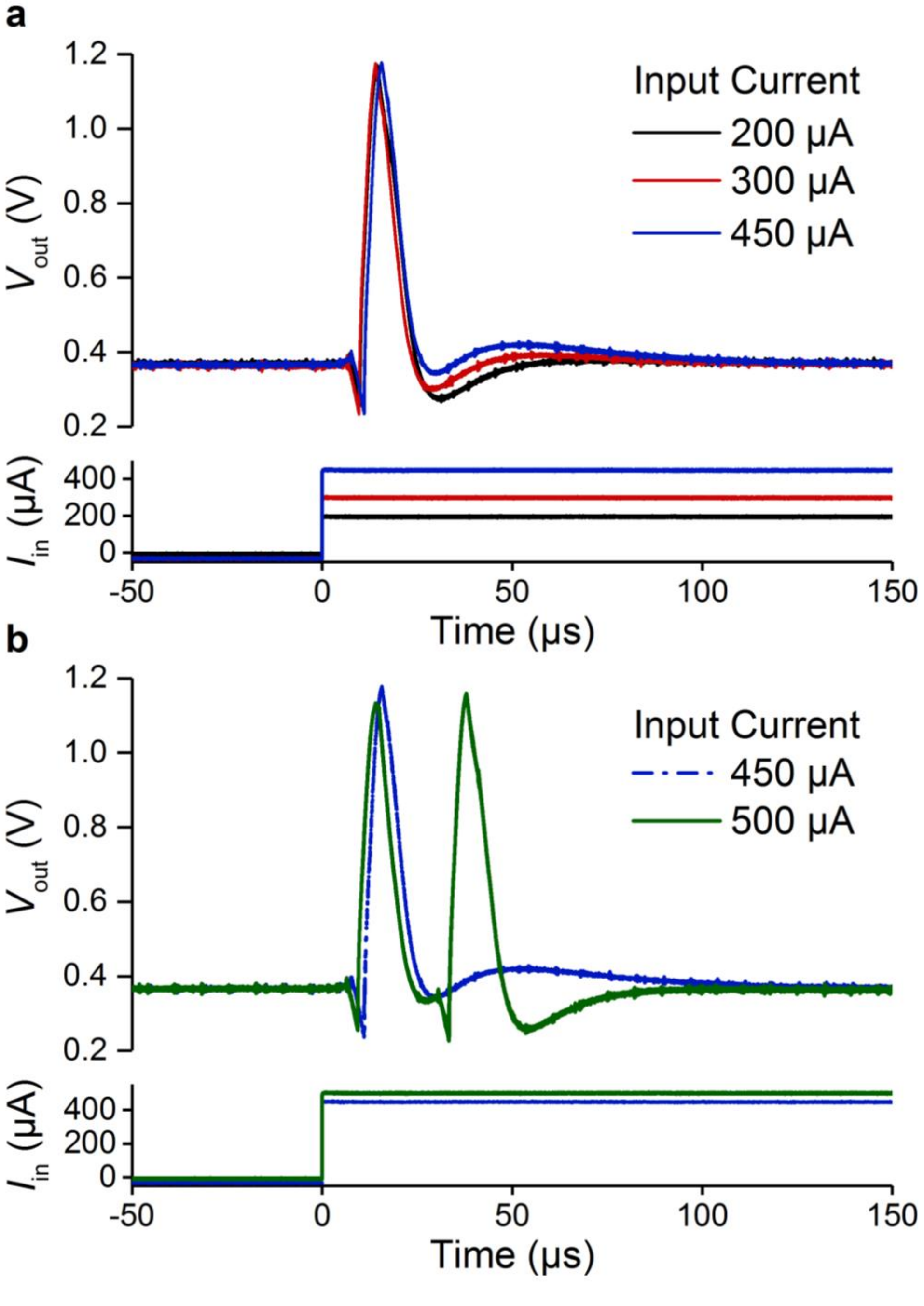

Supplementary Figure 32. Experimental depolarizing after-potential behavior of a phasic VO_2 neuron circuit. a, Single phasic spikes fired at the onset of d.c. input currents of 200 µA, 300 µA, and 450 µA levels. At relatively weaker input currents, the neuron membrane potential develops the commonly seen hyperpolarizing after-potential (HAP) that goes below the resting level. As the input strengthens, the HAP gradually weakens and morphs into a depolarizing after-potential (DAP) that goes above the resting level. **b**, Once a DAP is developed, the neuron has shortened refractory period and becomes superexcitable. A slightly stronger input, from 450 µA to 500 µA, causes the neuron to fire a second spike. The second spike is triggered by Na^+ channel reactivation when the relative refractory period is nullified by the formation of DAP.

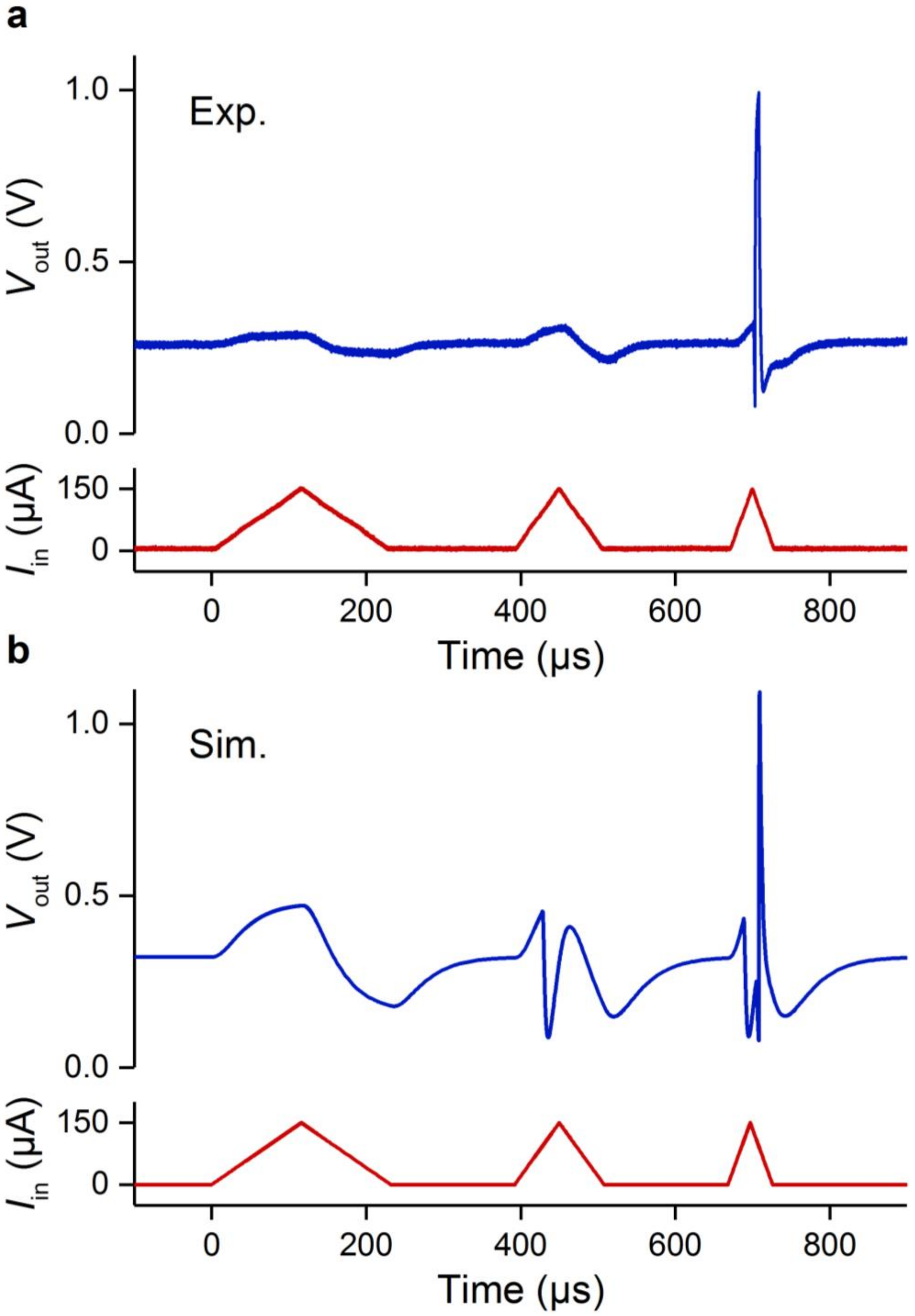

Supplementary Figure 33. Accommodation behavior in a phasic VO_2 neuron circuit. a, Experimental accommodation behavior, showing that a slowly ramped input current does not trigger the neuron to fire. In other words, the neuron accommodates the input change and becomes less excitable. A sharply ramped current, however, triggers a spike. All the current ramps have the same maximum amplitude of 150 µA. **b**, Simulated accommodation behavior of the same phasic VO_2 neuron circuit.

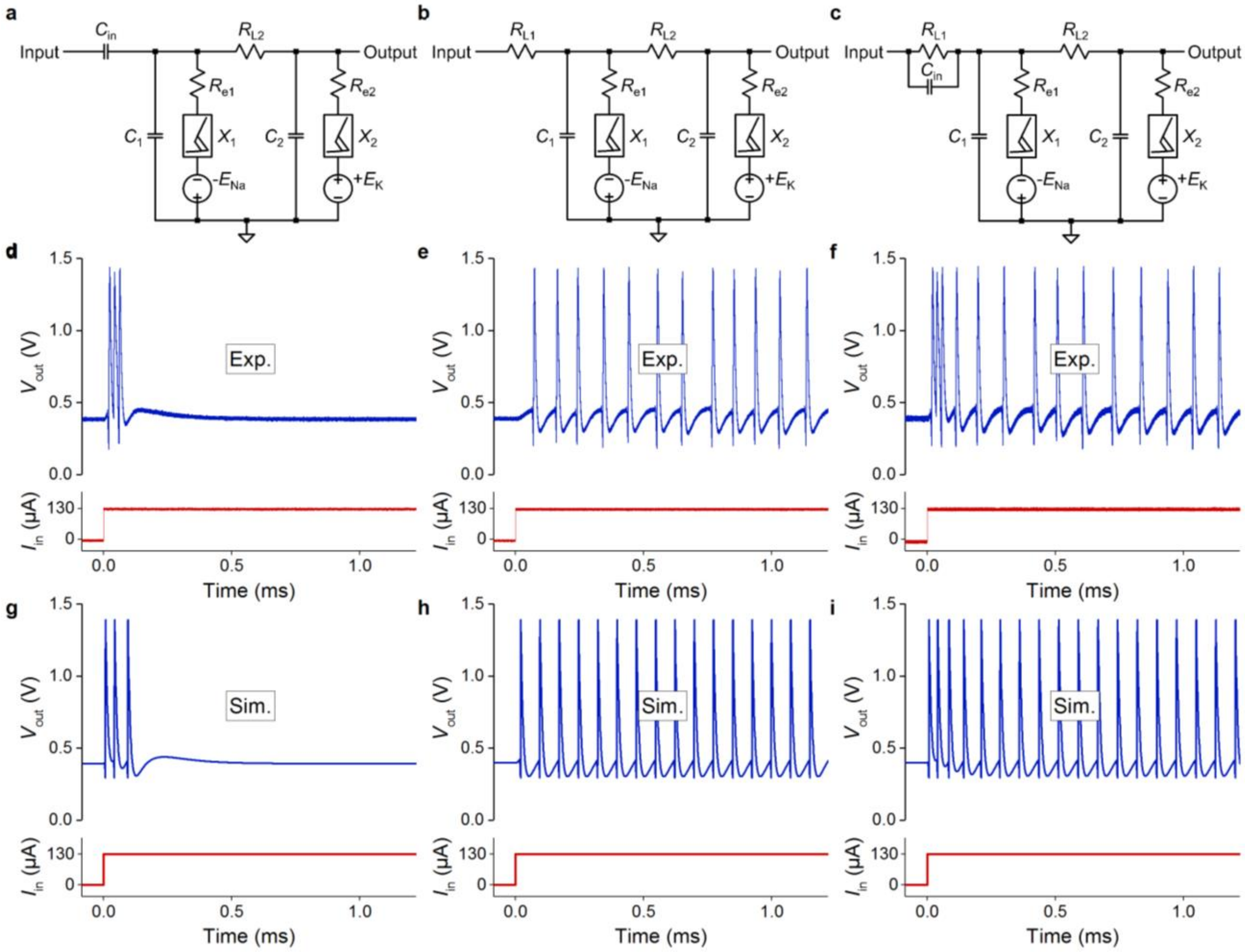

Supplementary Figure 34. Mixed-mode spiking behavior of a mixed-mode VO_2 neuron circuit. **a**, A phasic neuron with a capacitive coupling (C_{in}) to dendritic inputs. **b**, A tonic neuron with a resistive coupling (R_{L1}) to dendritic inputs. **c**, A mixed-mode neuron with both capacitive and resistive couplings (C_{in} in parallel with R_{L1}) to dendritic inputs. Except for the difference in input impedance, the tested neuron circuits in **a–c** are identical, including the VO_2 devices and d.c. biases used. **d**, Phasic bursting measured from the phasic neuron circuit in **a**. **e**, Tonic spiking measured from the tonic neuron circuit in **b**. **f**, Mixed-mode spiking, i.e., phasic bursting followed by tonic spiking, measured from the mixed-mode neuron circuit in **c**. **g**, Simulated phasic bursting of the phasic neuron circuit in **a**. **h**, Simulated tonic spiking of the tonic neuron circuit in **b**. **i**, Simulated mixed-mode spiking of the mixed-mode neuron circuit in **c**.

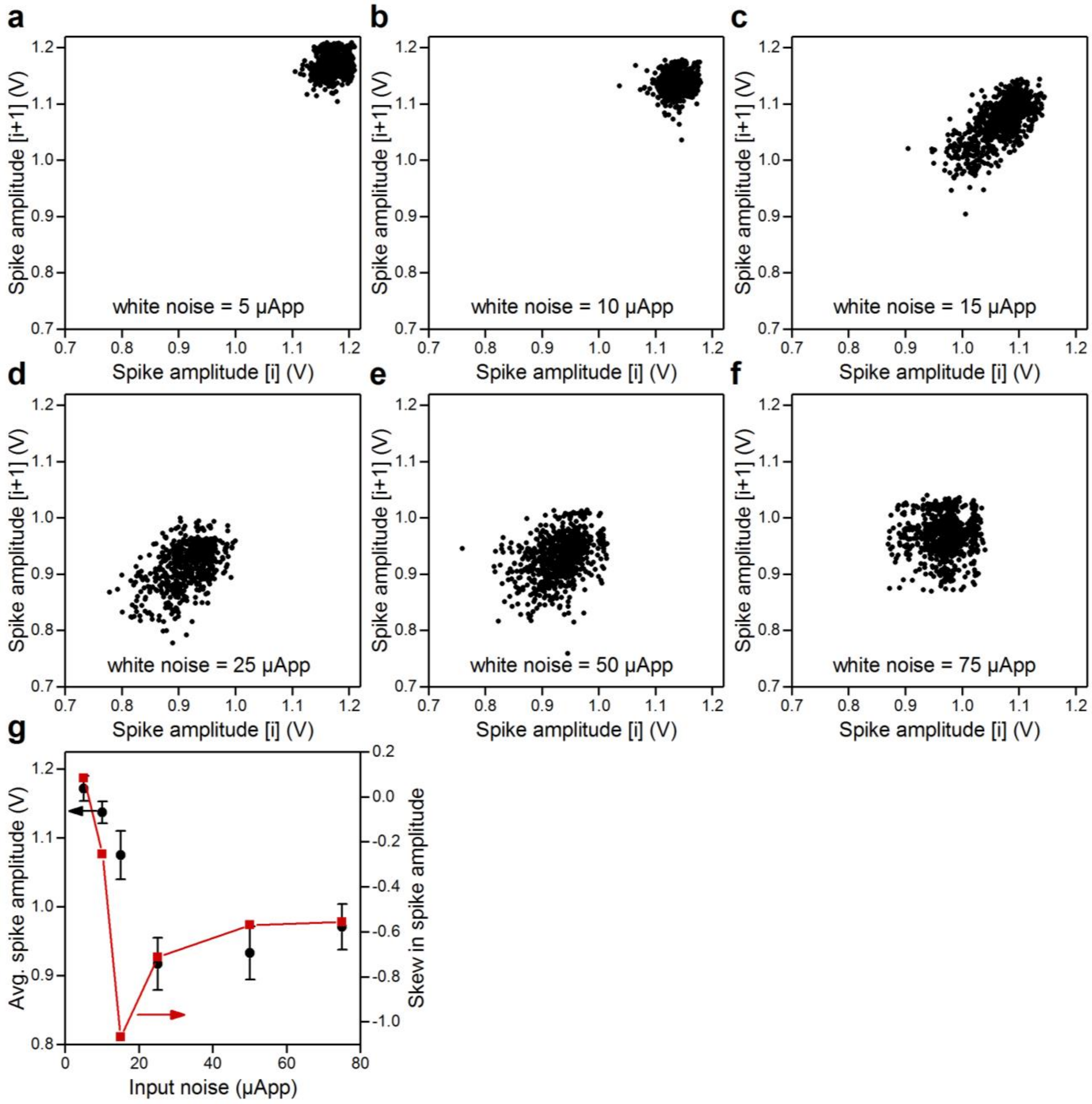

Supplementary Figure 35. Experimental recurrence plots (Poincaré plots) of spike amplitudes in a tonic VO_2 neuron circuit (from the same data as Fig. 6). a–f, Scatter recurrence plots of adjacent spike amplitudes at input white noise (peak-to-peak) levels of 5 µApp, 10 µApp, 15 µApp, 25 µApp, 50 µApp, and 75 µApp, respectively, showing that irregularities develop in both the spike timing (See Fig. 6) and the spike amplitude as the input stimulus becomes noisier. **g**, Dependences of the mean spike amplitude and the skewness in its distribution on the input noise level, showing similar trends of initially a fast decrease with the input noise, then a partial recovery if the input noise level is higher than ~20 µApp.

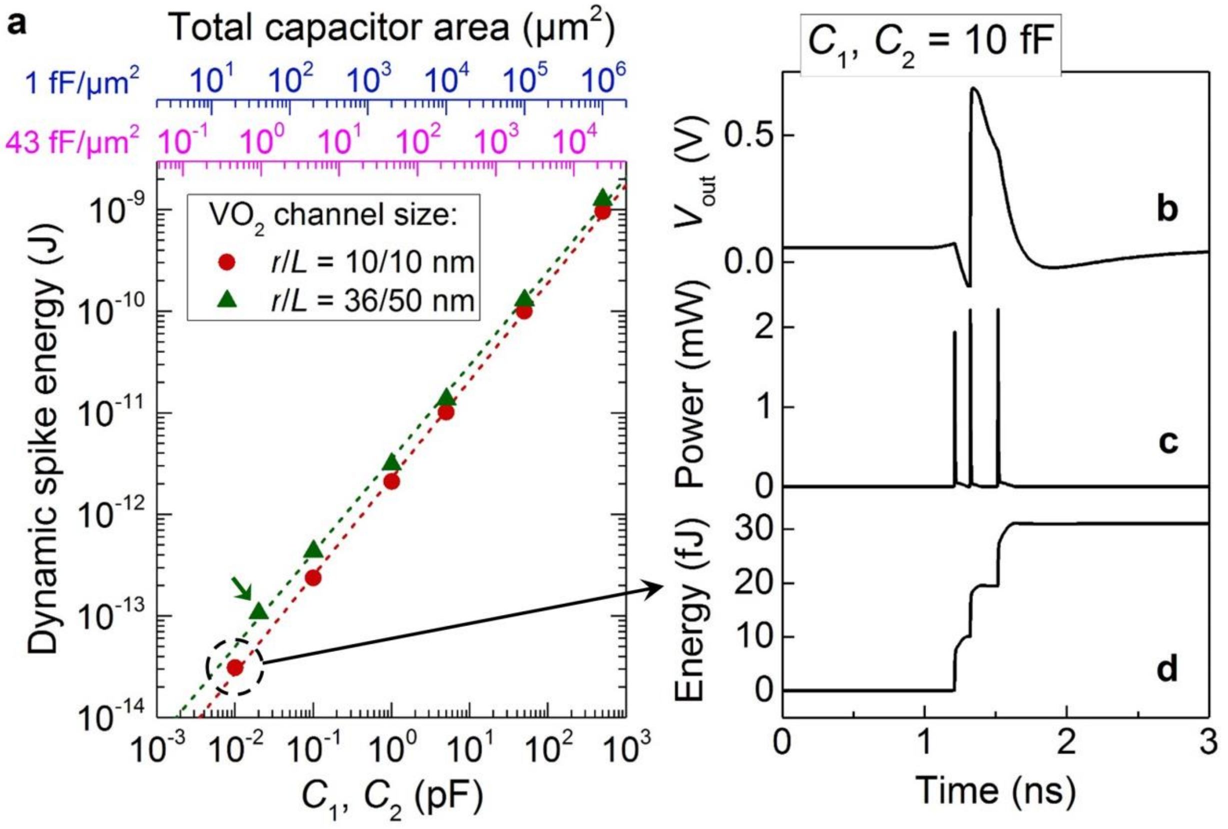

Supplementary Figure 36. Simulated dynamic power scaling of a VO_2 neuron. a, Simulated dynamic spike energy of a tonic VO_2 model neuron circuit vs. membrane capacitances, showing a nearly linear scaling with the capacitor values that also determine the neuron area. This is because the energy dissipated in a spike comes from the coordinated discharging of the two membrane capacitors. The dynamic spike energy is calculated by first summing the total power supplied by the $-E_{Na}$ and $+E_K$ voltage sources, then integrating over time (see example in **b–d**). For simplicity, $C_1 = C_2$ was assumed in the simulations. Two sets of VO_2 channel dimensions of r/L = 10/10 nm and r/L = 36/50 nm are compared. Here r is the VO_2 channel radius, L is the channel length (film thickness). The results show that the impact of the VO_2 channel dimensions on the neuron dynamic spike energy is relatively small. By aggressively shrinking the VO_2 volume by a factor of 18×, from r/L = 36/50 nm to r/L = 10/10 nm, the spike energy is only reduced by 24 %. The top x axes show the calculated total capacitor area at capacitance density of 1 fF μm^{-2} (blue) and 43 fF μm^{-2} (magenta), respectively. A dynamic spike energy use <0.1 pJ/spike (green arrow) can be achieved at a total capacitor area of ~1 μm^2 by using 20 fF membrane capacitors, which can be realized by today's integrated high-κ metal-insulator-metal (MIM) capacitors with a record-high capacitance density[21] of 43 fF μm^{-2} (typical MIM capacitance density of high-κ dielectrics is in the range of 15–20 fF μm^{-2}). Note that at a given capacitor area, lower capacitance value (by using lower capacitance density) will result in a lower spiking energy and hence a better EE. This is clearly shown in the EE–area scaling trend lines of VO_2 neurons (See Supplementary Fig. 2). **b**, Time dependent neuron spike waveform at C_1, C_2 = 10 fF and r/L = 10/10 nm (the circled red dot in **a**). **c**, Time dependent total dynamic power supplied by the $-E_{Na}$ and $+E_K$ voltage sources. **d**, Time dependent dynamic energy consumption, calculated by integrating the total dynamic power simulated in **c** over time.

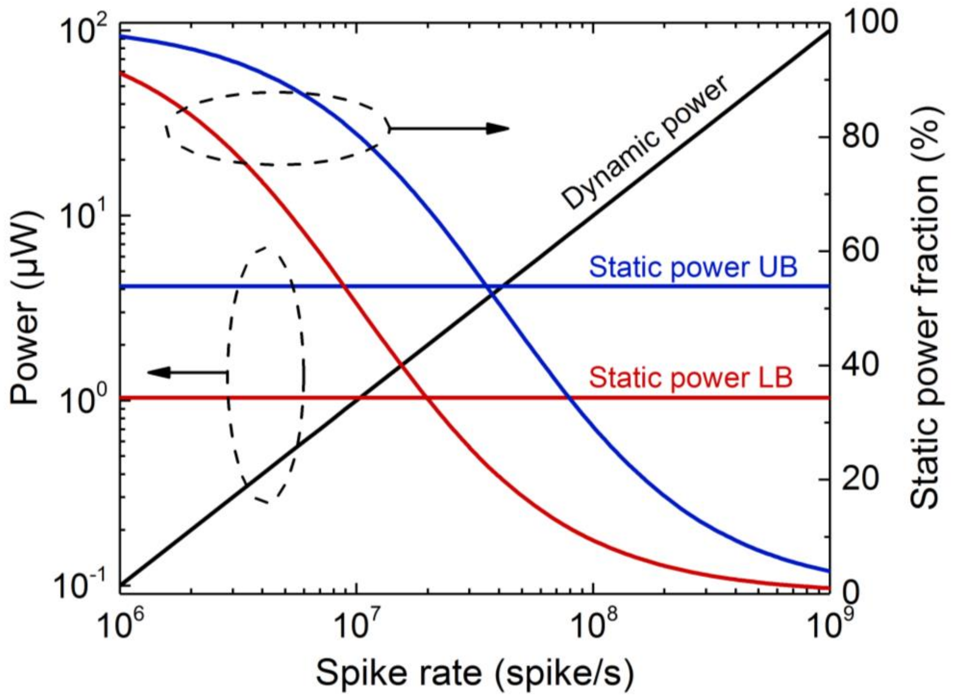

Supplementary Figure 37. Simulated dynamic and static power scaling of a VO_2 neuron over its spike rate. Left axis shows simulated dynamic and static power consumptions of a tonic VO_2 model neuron, and the right axis shows the percentage of static power in the total power consumption. Static power is dissipated by the neuron membrane leakage current in the resting state, i.e., leakage current drawn by d.c. biased VO_2 devices due to the finite resistivity of the insulating phase (~1 Ω·cm). With V_{th} as the switching threshold voltage, the upper bound (UB) and lower bound (LB) of static power are calculated at d.c. bias $V_{dc} = V_{th}$ and $V_{th}/2$, respectively. Since the neuron only spikes if $(V_{in} + V_{dc}) \geq V_{th}$, the signal gain, capped by V_{dc}/V_{in}, is always smaller than $V_{dc}/(V_{th} – V_{dc})$. The gain will becomes less than 1 if V_{dc} is less than $V_{th}/2$. While static power dissipation is independent of the spike rate, dynamic power dissipation is proportional to the spike rate. Therefore at low spike rates, static power may dominate the total power consumption. At sufficiently high spike rates, the static power makes only an insignificant contribution to the total power consumption, and is not expected to be of major concern for system level energy efficiency. The LB and UB of static power is less than 10 % of the total power at a spike rate higher than 100 MHz and 400 MHz, respectively, and the overall energy use is better than 0.11 pJ/spike. At 100 MHz spike rate, the single neuron total power consumption is 11 μW (LB) to 14 μW (UB). The SPICE simulation assumes a VO_2 channel of r/L = 10/10 nm, and the VO_2 model neuron has an energy use of 0.1 pJ/spike at C_1, C_2 = 38.3 fF (see the red dashed line in Supplementary Fig. 36a for details).

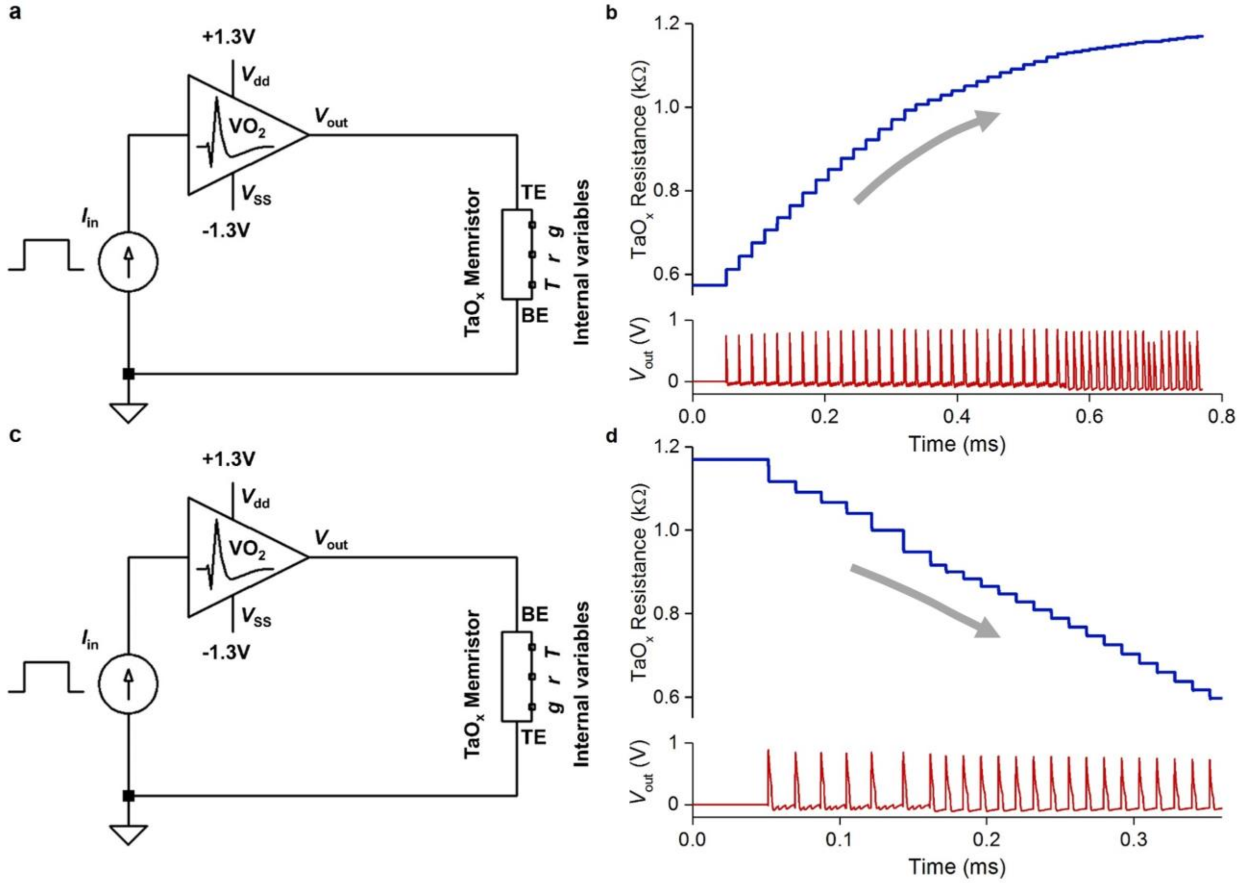

Supplementary Figure 38. Simulated potentiation and depression of a TaO_x memristor synapse by a VO_2 tonic neuron. a, Circuit diagram of the simulated depression (reset) of a TaO_x memristor with its top electrode connected to the output of a VO_2 tonic neuron circuit (the amplifier symbol). The VO_2 neuron fires a spike train in response to a square wave current input. **b**, Evolution of the TaO_x device resistance over time in circuit **a**, showing that each step in resistance rise is caused by a presynaptic VO_2 neuron spike. **c**, Circuit diagram of the simulated potentiation (set) of a TaO_x memristor with its bottom electrode connected to the VO_2 neuron output. **d**, Evolution of the TaO_x device resistance over time in circuit **c**, showing that each step in resistance drop is caused by a presynaptic VO_2 neuron spike. The TaO_x memristor SPICE model is from Ref. 22.

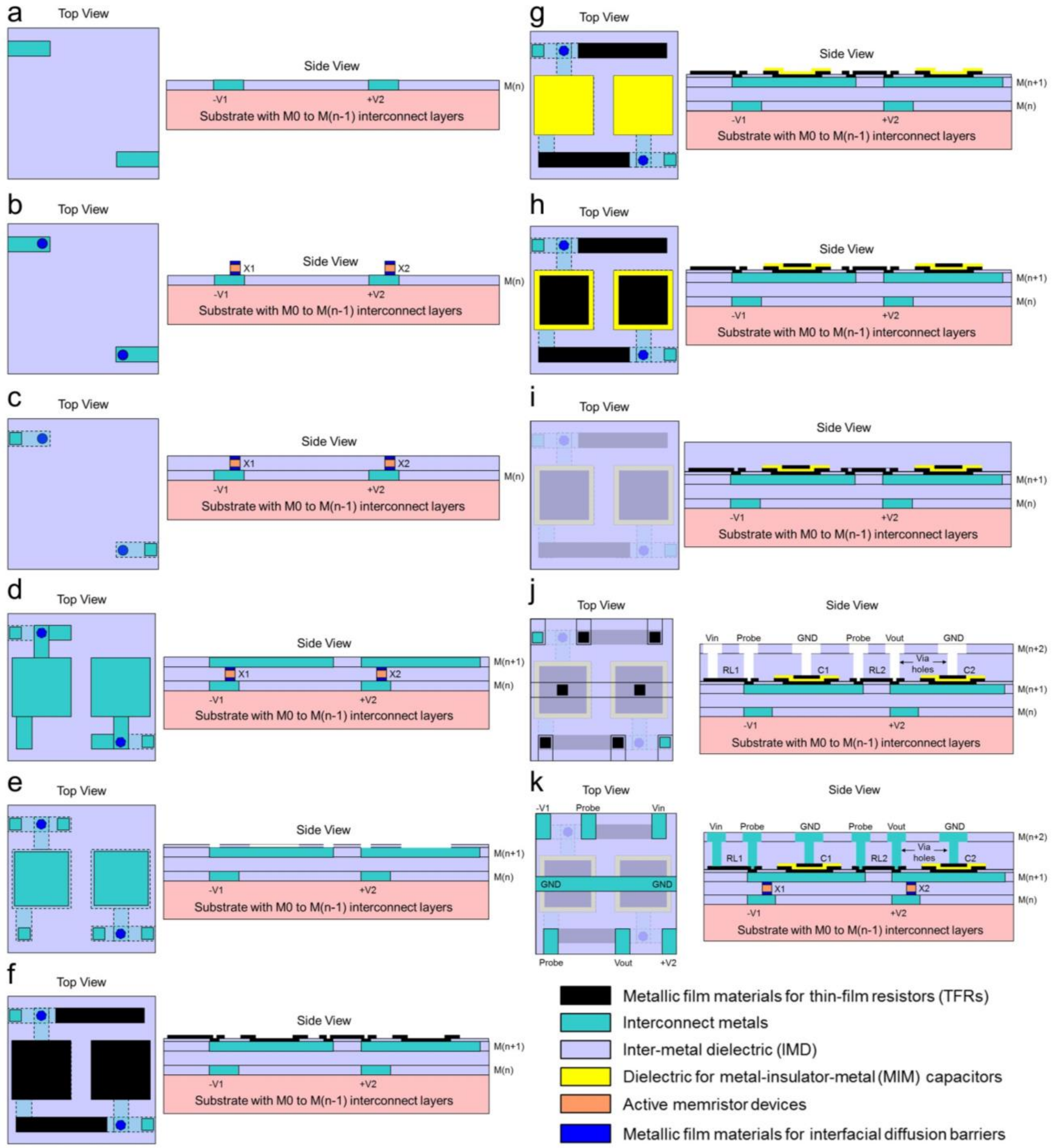

Supplementary Figure 39. Schematic top view and side cross-section view (not to scale) of possible process steps to manufacture an integrated active memristor neuron circuit. The complete integrated circuit only requires up to three layers of interconnect metals (M(n) to M(n+2)). The structures made in each process steps are: **a**, Bottom electrodes (BE) of memristors at M(n). **b**, Active memristor device stack that include metallic diffusion barriers. **c**, Inter-metal dielectric. **d**, Top electrodes (TE) of memristors (also as bottom electrodes of capacitors) at M(n+1). **e**, Dielectric passivation and contact openings of the TE. **f**, Thin-film resistors (also as the bottom contact layer for capacitors). **g**, Dielectric for capacitors. **h**, Top contact layer for capacitors. **i**, Inter-metal dielectric. **j-k**, Contact pads at M(n+2) by a dual-damascene process. Other methods, e.g. single damascene, can also be used.

Supplementary Tables

Supplementary Table 1. Comparison of volumetric free energy cost for Mott phase transitions in NbO_2 and VO_2 materials. Values for NbO_2 are from Table 1 in Ref. 16 (and references therein). At the same volume, the free energy cost for NbO_2 phase transition is 6.1 times that for VO_2. The total volumetric enthalpy change in a VO_2 nano-crossbar device with channel radius r = 10 nm and length L = 10 nm is merely 1.15 fJ.

**Material**	**NbO_2**	**VO_2**
Mott transition critical temperature T_C	1080 K	340 K
Temperature rise ΔT	800 K	43 K
Volumetric heat capacity c_p	2.6×10^6 J m^{-3} K^{-1}	3.3×10^6 J m^{-3} K^{-1}
Volumetric enthalpy change of IMT Δh_{tr}	1.6×10^8 J m^{-3}	2.35×10^8 J m^{-3}
Total volumetric enthalpy change	2.24×10^9 J m^{-3}	3.67×10^8 J m^{-3}
Factor of improvement	–	6.1×

Supplementary Table 2. Experimental circuit parameters used in neuron spiking tests. The VO_2 nano-crossbar devices X_1 and X_2 with the same nominal size of 100 × 100 nm^2 and film thickness of 100 nm are randomly selected from the same wafer. C_1 and C_2 values in the table are the values of discrete capacitors connected through coaxial cables to VO_2 devices. In simulations, stray capacitances in the setup, typically in the range of ~1 nF, are added to C_1 and C_2 values.

Spiking behavior	Figure No.	R_{L1} (kΩ)	R_{L2} (kΩ)	C_1 (nF)	C_2 (nF)	C_{in} (nF)	$-E_{Na}$ (V)	$+E_K$ (V)	X_1 (ID)	X_2 (ID)
All-or-nothing	S10	6	6	2	2	–	-1.35	1.35	5251-13	5251-9
Refractory period	S11	5	5	5	5	–	-1.6	1.6	5050-15	5050-7
Absolute & relative refractory periods	S12	6	6	4	1	–	-1.45	1.45	5352-1	5252-13
Tonic spike	S13	5	5	5	2	–	-1.5	1.5	5151-7	5151-3
Tonic burst	S14	10	10	5–30	0	–	-1.85	1.85	5051-9	5051-5
Class 1 excitable	5c	5	5	5	5	–	-1.5	1.5	5151-7	5151-3
Class 2 excitable	5b	5	5	1	5	–	-1.5	1.5	5151-7	5151-3
Spike frequency adaptation (tonic)	S15	10	10	200	2	–	-1.4	1.4	5251-13	5251-9
Spike frequency adaptation (phasic)	S16	–	9	4	1.2	9	-1.6	1.6	5351-11	5351-7
Spike latency	S17	6	6	10	3	–	-1.5	1.5	5352-1	5252-13
Subthreshold oscillation	S18	5	5	2	3	–	-1.4	1.4	5350-11	5350-7
Integrator	S19	6	6	8.5	2	–	-1.4	1.4	5251-13	5251-9
Bistability	S20	0	7	1.5	2	–	-1.58	1.58	5352-1	5252-13
Inhibition-induced spike	S21	6	6	6	2	–	-1.4	1.4	5251-13	5251-9
Inhibition-induced burst	S22a	6	6	35	0	–	-1.4	1.4	5251-13	5251-9
	S22b	7	7	21	0	–	-1.5	1.5	5049-3	4949-15
Excitation block	S23	6	6	0	2	–	-1.4	1.4	5251-13	5251-9
Resonator	S24	5	7	5	0	5	-1.5	1.5	5250-13	5250-9
Phasic spike	S25	–	7	1	2	0.3	-1.6	1.6	5352-1	5252-13
Phasic burst	S26	–	7	4	0	0.3	-1.6	1.6	5352-1	5252-13

(Continued on next page)

Spiking behavior	Figure No.	R_{L1} (kΩ)	R_{L2} (kΩ)	C_1 (nF)	C_2 (nF)	C_{in} (nF)	$-E_{Na}$ (V)	$+E_K$ (V)	X_1 (ID)	X_2 (ID)
Rebound spike	S27–S29	–	5.9	0	1	0.3	-1.5	1.5	5352-1	5252-13
Rebound burst	S30	–	5.9	0	0.5	0.3	-1.5	1.5	5352-1	5252-13
Threshold variability	S31	–	5.9	0	0.5	0.3	-1.5	1.5	5352-1	5252-13
Depolarizing after-potential	S32	–	6	0.9	2	0.3	-1.3	1.3	5352-1	5252-13
Accommodation	S33	–	7	1	0	0.3	-1.68	1.68	5352-1	5252-13
Mixed mode	S34	240	9	4	1.2	1	-1.6	1.6	5351-11	5351-7
Skipping	S35	7	7	1	1	–	-1.5	1.5	5149-11	5149-7

Supplementary Table 3. VO_2 material and structural parameters used in SPICE model simulations. In most cases, the Mott physics-based analytical compact SPICE model can faithfully reproduce experimental VO_2 device switching dynamics and neuron spiking behaviors (See all the simulated spiking patterns in Supplementary Figures). The model uses published VO_2 material properties. All the simulated neuron behaviors used the same VO_2 device model with a cylindrical-shaped VO_2 conduction channel of 56 nm in radius and 100 nm in length to match the actual VO_2 crystal volume in 100 × 100 nm^2 sized and 100 nm-thick nano-crossbar devices used in the experiments, and only varied the values of R, C elements. Series electrode resistance of 150–500 Ω, and parallel VO_2 channel leakage resistance of 13 kΩ to 17 kΩ were included in simulations to take into account their effects on the voltage drop across the memristors and the standby current in the insulating phase. 'Exp.' stands for experimentally determined.

Memristor Model Property	Symbol	Model Value	Unit	Reference
Volumetric heat capacity	c_p	3.30×10^{6}	$J\ m^{-3}\ K^{-1}$	23
Volumetric enthalpy change of IMT	Δh_{tr}	2.35×10^{8}	$J\ m^{-3}$	24
Thermal conductivity of insulating phase	κ	3.5	$W\ m^{-1}\ K^{-1}$	23
Metallic phase electrical resistivity	ρ_{met}	3×10^{-6}	Ω·m	23,24
Insulating phase electrical resistivity	ρ_{ins}	1×10^{-2}	Ω·m	24
Temperature rise	ΔT	43	K	24
Conduction channel radius	r_{ch}	56	nm	Exp.
Conduction channel length	L_{ch}	100	nm	Exp.
Series electrode resistance	R_e	150 to 500	Ω	Exp.
Parallel leakage resistance	R_{shunt}	13k to 17k	Ω	Exp.

Supplementary Table 4. Biological fidelity and computational cost of neuron models in comparison with experimentally demonstrated biological fidelity of VO_2 neurons. The neurocomputational properties of neuron models are adopted and augmented from Fig. 2 in Ref. 18. "# of FLOPS" is the approximate number of floating point operations needed to simulate the neuron model for a 1 ms duration using a digital computer. (+), (-) and empty square represents possessed, missing, and unconfirmed properties of the model. For VO_2 neurons, the only property that remains unconfirmed is chaos.

Neuron Models	All-or-nothing firing	Refractory period	Excitation block	Biophysically meaningful	Tonic spiking	Phasic spiking	Tonic bursting	Phasic bursting	Mixed mode	Spike frequency adaptation	Class 1 excitable	Class 2 excitable	Spike latency	Subthreshold oscillations	Resonator	Integrator	Rebound spike	Rebound burst	Threshold variability	Bistability	DAP	Accommodation	Inhibition-induced spiking	Inhibition-induced bursting	Chaos	# of FLOPS
integrate-and-fire	+	+	-	-	+	-	-	-	-	-	+	-	-	-	-	+	-	-	-	-	-	-	-	-	-	5
integrate-and-fire with adapt.	+	+	-	-	+	-	-	-	-	+	+	-	-	-	-	+	-	-	-	-	+	-	-	-	-	10
integrate-and-fire-or-burst	+	+	-	-	+	+		+	-	+	+	-	-	-	-	+	+	+	-	+	+	-	-	-		13
resonate-and-fire	+	+	+	-	+	+	-	-	-	-	+	+	-	+	+	+	+	-	-	+	+	+	-	-	+	10
quadratic integrate-and-fire	+	+	-	-	+	-	-	-	-	-	+	-	+	-	-	+	-	-	+	+	-	-	-	-	-	7
Izhikevich (2003)	+	+	+	-	+	+	+	+	+	+	+	+	+	+	+	+	+	+	+	+	+	+	+	+	+	13
FitzHugh-Nagumo	*	+	+	-	+	+	-		-	-	+	-	+	+	+	-	+	-	+	+	-	+	+	-	-	72
Hindmarsh-Rose	+	+	+	-	+	+	+			+	+	+	+	+	+	+	+	+	+	+	+	+	+		+	120
Morris-Lecar	+	+	+	+	+	+	-		-	-	+	+	+	+	+	+	+		+	+	-	+	+	-	-	600
Wilson	+	+	+	-	+	+	+			+	+	+	+	+	+	+	+	+	+		+	+				180
Hodgkin-Huxley	*	+	+	+	+	+	+			+	+	+	+	+	+	+	+	+	+	+	+	+	+		+	1200
HRL VO_2 Neuron	+	+	+	+	+	+	+	+	+	+	+	+	+	+	+	+	+	+	+	+	+	+	+	+		

Experimentally demonstrated

Supplementary Notes

Supplementary Note 1: VO_2 active memristor relaxation oscillator

VO_2 is well-known for its first-order thermodynamically-driven Mott insulator-to-metal (IMT) phase transition with a critical temperature T_C near 67 °C[25]. Joule heating produced by electrical current through a metal/VO_2/metal device generates Mott IMT-induced volatile hysteretic resistive switching and an NDR regime, which forms the basis to construct oscillators, amplifiers, and impulse circuits (neurons). Mott memristors, a type of active memristors based on Mott IMT, were previously realized by producing crystalline NbO_2 in an electroforming process from amorphous Nb_2O_5 films[26]. Electroformed devices suffer from large device variability that is undesirable for integration. In our case, electroform-free VO_2 active memristor nano-crossbar devices with typical device yield of 98–100 %, low-voltage (down to ~0.5 V), high-endurance, and low device variability (<13 % coefficient of variation in switching threshold voltage) are fabricated on CMOS-compatible 3-inch SiN_x-coated silicon substrates (See Methods). Pearson-Anson (PA) relaxation oscillator is the prototype electronic circuit analogue for voltage-gated Na^+ or K^+ nerve membrane ion channels. Other ion channels, e.g. Cl^- or Ca^{2+}, can also be emulated in a similar manner. If two such relaxation oscillators are coupled with proper impedance, the overall circuit can generate an action potential[26-28]. Supplementary Fig. 3 shows the circuit diagram and astable oscillator characteristics measured in a VO_2 relaxation oscillator. A one-to-one correspondence can be identified between the quasi d.c. V–I trace (force V, measure I) of the VO_2 device (without the capacitor) and the V–time and I–time waveforms of the astable oscillations under an external d.c. bias V_{dc}[17]. For astable oscillation to occur, the load line, defined by V_{dc} and the load resistor R_L, must intersect the V–I curve in its NDR regime. A complete cycle of astable oscillation, from point (1) to (5), has four stages as explained by figure caption. The actual switching time scale of VO_2 is much faster than the rise time of the oscilloscope and cannot be measured. Values ranging from 100 fs to 5 ps have been measured by pump-probe methods[29,30].

Although many transition metal oxides exhibit Mott IMT, T_C in many of these materials are well below 300 K (room temperature). Mott insulators with T_C > 300 K, e.g. VO_2, Ti_2O_3, Ti_3O_5, NbO_2, $SmNiO_3$, $LaCoO_3$, are more suitable for electronic applications[31]. NbO_2 is a demonstrated material for spiking neurons[26]. However, its T_C of 1080 K requires a large local temperature rise of 800 K to operate, which negatively impacts both power consumption and device longevity[31] (See Supplementary Table 1 for volumetric free energy cost of Mott IMT in NbO_2 and VO_2). We applied SPICE simulations of a VO_2-based relaxation oscillator to estimate the switching energy and switching time (speed) of Mott IMT[26]. The switching time of the phase transition is estimated from the rising edges of device current in each oscillation period. The VO_2 channel radius is varied while all the other model parameters, including the channel length (50 nm), are fixed. As shown in Supplementary Fig. 9, at the same channel dimensions, simulated Mott IMT switching in VO_2 is 100 times faster than in NbO_2, and only consumes about one-sixth (16 %) of the energy. Note that the switching speed is a material-dependent parameter, and is not affected by the values of R, C passive elements. <1 fJ switching energy and <1 ps switching speed can be achieved at VO_2 channel radius of 7–15 nm, dimensions feasible for advanced-node lithography.

Supplementary Note 2: Device modeling of VO_2 active memristors

We use the same analytical mathematical equations developed by the authors of Ref. 16 to model the dynamics of VO_2 active memristors. The VO_2 model parameters are summarized in Supplementary Table 3. The main equations are relisted below:

$$v = R_{\mathrm{ch}}(u) \cdot i \tag{1}$$

$$\frac{du}{dt} = \left(\frac{d\Delta H}{du}\right)^{-1} \cdot \left(i^2 R_{\mathrm{ch}}(u) - \Gamma_{\mathrm{th}}(u)\Delta T\right) \tag{2}$$

$$R_{\mathrm{ch}}(u) = \frac{\rho_{\mathrm{ins}} L_{\mathrm{ch}}}{\pi r_{\mathrm{ch}}^2}\left[1 + \left(\frac{\rho_{\mathrm{ins}}}{\rho_{\mathrm{met}}} - 1\right) u^2\right]^{-1} \tag{3}$$

$$\Gamma_{\mathrm{th}}(u) = 2\pi L_{\mathrm{ch}} \kappa \left(\ln\frac{1}{u}\right)^{-1} \tag{4}$$

$$\frac{d\Delta H}{du}(u) = \pi L_{\mathrm{ch}} r_{\mathrm{ch}}^2 \left[c_p \Delta T \frac{1 - u^2 + 2u^2 \ln u}{2u(\ln u)^2} + 2\Delta h_{\mathrm{tr}} u\right] \tag{5}$$

Supplementary Eq. (1) is the instantaneous Ohm's law relationship between current and voltage, wherein the VO_2 channel resistance $R_{ch}(u)$ is determined by a single state variable $u \triangleq r_{\mathrm{met}}/r_{\mathrm{ch}}$, i.e. the normalized radius of the metallic cylindrical conducting channel heated above the T_C of Mott transition. Supplementary Eq. (2) is the first-order differential equation that drives the state dynamics. Supplementary Eqs. (3)–(5) are equations for three auxiliary functions, including Supplementary Eq. (3) for the state-dependent resistance $R_{\mathrm{ch}}(u)$, Supplementary Eq. (4) for the state-dependent thermal conductance $\Gamma_{\mathrm{th}}(u)$, and Supplementary Eq. (5) for the differential change of enthalpy $d\Delta H/du$ with respect to the state u.

The SPICE compact model of VO_2 devices is constructed in a similar manner as outlined in the supplementary materials of Ref. 16. All the SPICE simulations were performed on a personal computer using the LTspice IV software.

Supplementary Note 3: Dynamics equations of an active memristor neuron circuit

Starting with the active memristor device model equations from Ref. 16, after applying Kirchhoff's voltage law (KVL) and Kirchhoff's current law (KCL), we derived the four coupled first-order ordinary differential equations (ODEs) that drive the dynamics of a model tonic active memristor neuron circuit (See Supplementary Fig. 40). Similar procedure can be applied to derive the model equations for phasic neuron circuits, which are not included here. The reference convention is that the potential inside the nerve cell is fixed at the ground level. A positive current flows toward the ground, and a positive voltage will produce a current flowing toward the ground. Following the convention in biological neuron models, the d.c. biases $-E_{Na}$ and $+E_K$ applied on the two active memristors X_1 and X_2 are rewritten as electromotive forces E_1 and E_2 (amplitudes only). Their polarities are taken care of when incorporated into equations. For simplicity, we temporarily assume that there is no external load that draws a current at the cell output, and only consider the situation of current clamp, i.e. an external input current I is fed into the cell. The case for voltage clamp can be derived in a similar manner.

To simplify the expressions, let's define $\mathcal{H}(u) \triangleq (d\Delta H/du)^{-1}$for the differential change of enthalpy, $Q(u) \triangleq \Gamma_{\mathrm{th}}(u)\Delta T$ for the heat flux, and remove the subscript 'ch' of channel resistance in $R_{\mathrm{ch}}(u)$. The model equations for the two active memristors are rewritten as:

$$v_1 = R(u_1) \cdot i_1 \tag{6}$$

$$\frac{du_1}{dt} = \mathcal{H}(u_1) \cdot (i_1^2 R(u_1) - Q(u_1)) \tag{7}$$

$$v_2 = R(u_2) \cdot i_2 \tag{8}$$

$$\frac{du_2}{dt} = \mathcal{H}(u_2) \cdot (i_2^2 R(u_2) - Q(u_2)) \tag{9}$$

Currents flowing through the two membrane capacitors C_1 and C_2 are:

$$I_{C1} = C_1 \frac{d(v_1 - E_1)}{dt} = C_1 \frac{dv_1}{dt} \tag{10}$$

$$I_{C2} = C_2 \frac{d(v_2 + E_2)}{dt} = C_2 \frac{dv_2}{dt} \tag{11}$$

Applying KCL at the joint connecting R_{L1}, C_1, X_1 and R_{L2}, the external input current is

$$I = I_{C1} + i_1 + I_{C2} + i_2 \tag{12}$$

Substituting I_{C1}, i_1, I_{C2} and i_2 with Supplementary Eqs. (10), (6), (11) and (8), we have

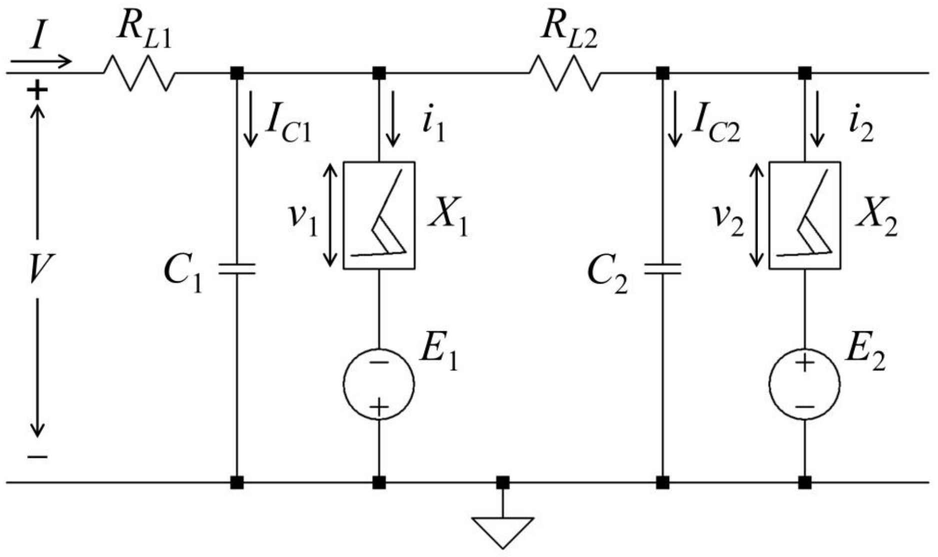

Supplementary Figure 40. Circuit diagram of a tonic memristor neuron circuit with legends of voltages and currents to assist modeling.

$$I = C_1\frac{dv_1}{dt} + \frac{v_1}{R(u_1)} + C_2\frac{dv_2}{dt} + \frac{v_2}{R(u_2)} \tag{13}$$

Applying KVL at the joint connecting R_{L1}, C_1, X_1 and R_{L2},

$$v_1 - E_1 = \left(C_2\frac{dv_2}{dt} + \frac{v_2}{R(u_2)}\right)R_{L2} + v_2 + E_2 \tag{14}$$

Supplementary Eq. (14) is then rewritten in the form of a first-order ODE:

$$\frac{dv_2}{dt} = \frac{1}{R_{L2}C_2}\left[v_1 - \left(1 + \frac{R_{L2}}{R(u_2)}\right)v_2 - E_1 - E_2\right] \tag{15}$$

Substituting dv_2/dt in Supplementary Eq. (13) with the formula of Supplementary Eq. (15), Supplementary Eq. (13) can be rewritten in the form of a first-order ODE:

$$\begin{aligned}\frac{dv_1}{dt} = \frac{1}{C_1}&\left\{I - \frac{v_1}{R(u_1)} - C_2\frac{dv_2}{dt} - \frac{v_2}{R(u_2)}\right\}\\ &= \frac{1}{C_1}\left\{I - \frac{v_1}{R(u_1)} - \frac{1}{R_{L2}}\left[v_1 - \left(1 + \frac{R_{L2}}{R(u_2)}\right)v_2 - E_1 - E_2\right] - \frac{v_2}{R(u_2)}\right\}\\ &= \frac{1}{C_1}\left\{I - \left(\frac{1}{R(u_1)} + \frac{1}{R_{L2}}\right)v_1 + \left(\frac{1}{R_{L2}} + \frac{1}{R(u_2)}\right)v_2 + \frac{E_1 + E_2}{R_{L2}} - \frac{v_2}{R(u_2)}\right\}\\ &= \frac{1}{C_1}\left\{I - \left(\frac{1}{R(u_1)} + \frac{1}{R_{L2}}\right)v_1 + \left(\frac{1}{R_{L2}}\right)v_2 + \frac{E_1 + E_2}{R_{L2}}\right\}\end{aligned}$$

Multiplying both sides by R_{L2}, it becomes:

$$\frac{dv_1}{dt} = \frac{1}{R_{\mathrm{L2}} C_1} \left[I \cdot R_{\mathrm{L2}} - \left(1 + \frac{R_{\mathrm{L2}}}{R(u_1)}\right) v_1 + v_2 + E_1 + E_2 \right] \tag{16}$$

Supplementary Eqs. (7), (9), (15) and (16) are the four coupled first-order ODEs that solve the four state variables(u_1, v_1, u_2, v_2) which drive the dynamics of the neuron circuit. They are grouped together as below

$$\begin{cases} v_1' = \frac{1}{R_{\mathrm{L2}} C_1} \left[I \cdot R_{\mathrm{L2}} - \left(1 + \frac{R_{\mathrm{L2}}}{R(u_1)}\right) v_1 + v_2 + E_1 + E_2 \right] \\ v_2' = \frac{1}{R_{\mathrm{L2}} C_2} \left[v_1 - \left(1 + \frac{R_{\mathrm{L2}}}{R(u_2)}\right) v_2 - E_1 - E_2 \right] \\ u_1' = \mathcal{H}(u_1) \cdot \left(i_1^2 R(u_1) - Q(u_1) \right) \\ u_2' = \mathcal{H}(u_2) \cdot \left(i_2^2 R(u_2) - Q(u_2) \right) \end{cases}$$

Experimentally it's more convenient to probe the Na^+ and K^+ channel membrane potentials $V_{\mathrm{Na}} = v_1 - E_1$ and $V_{\mathrm{K}} = v_2 + E_2$. Substituting v_1 with V_{Na} and v_2 with V_{K} in the above equations, the dynamics equations can be recasted as

$$\begin{cases} V_{\mathrm{Na}}' = \frac{1}{C_1} \cdot \left[I - \left(\frac{1}{R_{\mathrm{L2}}} + \frac{1}{R(u_1)} \right) V_{\mathrm{Na}} + \frac{1}{R_{\mathrm{L2}}} V_{\mathrm{K}} - \frac{1}{R(u_1)} E_1 \right] & (17) \\ V_{\mathrm{K}}' = \frac{1}{C_2} \cdot \left[\frac{1}{R_{\mathrm{L2}}} V_{\mathrm{Na}} - \left(\frac{1}{R_{\mathrm{L2}}} + \frac{1}{R(u_2)} \right) V_{\mathrm{K}} + \frac{1}{R(u_2)} E_2 \right] & (18) \\ u_1' = \mathcal{H}(u_1) \cdot \left[\frac{(V_{\mathrm{Na}} + E_1)^2}{R(u_1)} - Q(u_1) \right] & (19) \\ u_2' = \mathcal{H}(u_2) \cdot \left[\frac{(V_{\mathrm{K}} - E_2)^2}{R(u_2)} - Q(u_2) \right] & (20) \end{cases}$$

We noted that the dynamic equations (17) and (18) have been presented in Ref. 32. However, in that reference, the dynamic equations of state variables u_1 and u_2 are not used for reduced-dimension V_{Na}–V_{K} nullcline analysis, instead a hard switching between two preset resistance values R_{ON} and R_{OFF} were assumed. Such an overly-simplified approach unavoidably will miss some important aspects of the nonlinear dynamics of Supplementary Eqs. (7) and (9). Applying Ohm's law, we rewrite Supplementary Eqs. (7) and (9) as Supplementary Eqs. (19) and (20), wherein the channel currents i_1 and i_2 are replaced by the corresponding membrane potentials V_{Na} and V_{K}.

Supplementary Note 4: Dynamic and static power scaling of VO_2 neurons

We use SPICE simulations to analyze the dynamic and static power scaling of tonic VO_2 neurons (See Supplementary Figs. 37 and 38). The dynamic spike energy is calculated by first summing the total power supplied by the d.c. voltage sources, then integrating over time through the course of a spike.

In Supplementary Fig. 36, the dynamic spiking energy scales almost linearly with the capacitance of membrane capacitors, with a power-law fitted slope of 0.96 at r/L = 10/10 nm and 0.924 at r/L = 36/50 nm, respectively. The neuron area also scales linearly with the membrane capacitance as capacitor elements dominate the circuit area. It is therefore desirable to make smaller neurons to achieve higher (dynamic) spiking energy efficiency (EE) (See Supplementary Fig. 2). Note that area scaling of membrane capacitors is not a limiting factor for the neuron area scaling, because memristor neurons do not require a minimum value of membrane capacitors to operate, and therefore there is no size constraint posted by the requirement on certain membrane capacitance value. Another observation is that since VO_2 switching energy is extremely low (See Supplementary Table 1), only 1.15 fJ/device at r/L = 10/10 nm, aggressive VO_2 device scaling is not needed: an 18-fold volume reduction, from r/L = 36/50 nm to 10/10 nm, only reduces the neuron spike energy by ~24 %.

If only considering dynamic power consumption for the case of r/L = 36/50 nm, <0.1 pJ/spike energy use can be achieved at a total capacitor area of ~1 μm^2 by using 20 fF membrane capacitors (see green arrow in Supplementary Fig. 36a), which can be realized by today's integrated high-κ metal-insulator-metal (MIM) capacitors with a record-high capacitance density[21] of 43 fF μm^{-2} (typical MIM capacitance density of high-κ dielectrics is in the range of 15–20 fF μm^{-2}). However, using high-κ dielectric to boost the capacitance density is not a good strategy to achieve higher EE. At a given capacitor area, lower capacitance value (by using lower capacitance density) will result in a lower spiking energy and hence a better EE. This is clearly shown in the EE-area scaling trend lines of VO_2 neurons (See Supplementary Fig. 2) stimulated at capacitance density of 1, 10, and 43 fF μm^{-2}. At the same neuron (and capacitor) area, lower capacitance density translates into lower dynamic spiking energy and hence higher EE. At 1 fF μm^{-2} capacitance density, VO_2 neurons show superior EE-area scaling than the best-case HH cells at neuron sizes smaller than 70 μm^2, and can surpass the estimated human brain EE of 1.8×10^{14} spike/J (or 5.6 fJ/spike energy use) at neuron sizes smaller than 3 μm^2.

The static power consumption is dissipated by standby current through d.c. biased VO_2 devices due to the finite resistivity of the insulating phase (~1 Ω·cm)[24]. At low firing rates, static power may dominate the total power consumption. Since the dynamic power is proportional to the firing rate, while the static power remains a constant, the percentage of static power in total power decreases with firing rate. The lower and higher bounds of static power, estimated at d.c. bias of V_{th} and $V_{th}/2$ (V_{th} is the switching threshold) respectively, is <10 % of the total power at a firing rate higher than 100 MHz and 400 MHz, respectively, and the overall energy use is lower than 0.11 pJ/spike. We have not considered the possibility that the insulating-phase resistivity of VO_2 can be improved. Note that the neuron EE is not the only factor that determines the network power consumption. The firing rate, the synapse resistance, and the synapse/neuron ratio also need to be considered.

Supplementary References

1 Esser, S. K. et al. Convolutional networks for fast, energy-efficient neuromorphic computing. *Proc. Nat. Acad. Sci.* **113**, 11441–11446 (2016).

2 Anonymous. GPU-based deep learning inference: a performance and power analysis. *Nvidia whitepaper* https://www.nvidia.com/content/tegra/embedded-systems/pdf/jetson_tx1_whitepaper.pdf (2015).

3 Ovtcharov, K. et al. Accelerating deep convolutional neural networks using specialized hardware. *Microsoft whitepaper* http://research.microsoft.com/apps/pubs/?id=240715 (2015).

4 Hauβmann, H. Comparing Google's TPUv2 against Nvidia's V100 on ResNet-50. *RiseML Blog* https://blog.riseml.com/comparing-google-tpuv2-against-nvidia-v100-on-resnet-50-c2bbb6a51e5e (2018).

5 Wijekoon, J. H. B. & Dudek, P. Compact silicon neuron circuit with spiking and bursting behavior. *Neural Netw.* **21**, 524–534 (2008).

6 Livi, P. & Indiveri, G. A current-mode conductance-based silicon neuron for Address-Event neuromorphic systems. *In IEEE Int. Symp. Circ. Sys.* https://doi.org/10.1109/ISCAS.2009.5118408 (2009).

7 Schemmel, J. et al. A wafer-scale neuromorphic hardware system for large-scale neural modeling. *2010 IEEE Int. Symp. Circ. Sys.* https://doi.org/10.1109/ISCAS.2010.5536970.

8 Yu, T., Park, J., Joshi, S., Maier, C. & Cauwenberghs, G. 65k-neuron integrate-and-fire array transceiver with address-event reconfigurable synaptic routing. *2012 IEEE Biomed. Circ. Sys. Conf.* https://doi.org/10.1109/BioCAS.2012.6418479 (2012).

9 Cruz-Albrecht, J. M., Yung, M. W. & Srinivasa, N. Energy-efficient neuron, synapse and STDP integrated circuits. *IEEE Trans. Biomed. Circuits Syst.* **6**, 246-256 (2012).

10 Joubert, A., Belhadj, B., Temam, O. & Heliot, R. Hardware spiking neurons design: analog or digital? *2012 IEEE Int. J. Conf. Neural Netw.* https://doi.org/10.1109/IJCNN.2012.6252600 (2012).

11 Park, J., Ha, S., Yu, T., Neftci, E. & Cauwenberghs, G. A 65k-neuron 73-Mevents/s 22-pJ/event asynchronous micro-pipelined integrate-and-fire array transceiver. *2014 IEEE Biomed. Circ. Sys. Conf.* https://doi.org/10.1109/BioCAS.2014.6981816 (2014).

12 Benjamin, B. V. et al. Neurogrid: a mixed-analog-digital multichip system for large-scale neural simulations. *Proc. IEEE* **102**, 699-716 (2014).

13 Merolla, P. A. et al. A million spiking-neuron integrated circuit with a scalable communication network and interface. *Science* **345**, 668-673 (2014).

14 Sengupta, B., Faisal, A. A., Laughlin, S. B. & Niven, J. E. The effect of cell size and channel density on neuronal information encoding and energy efficiency. *J. Cerebral Blood Flow & Metabolism* **33**, 1465–1473 (2013).

15 Hasler, J. M., B. Finding a roadmap to achieve large neuromorphic hardware systems. *Front. Neurosci.* **7**, 118 (2013).

16 Pickett, M. D. & Williams, R. S. Sub-100 fJ and sub-nanosecond thermally driven threshold switching in niobium oxide crosspoint nanodevices. *Nanotechnol.* **23**, 215202 (2012).

17 Gruver, G. W. *A study of one-port negative resistance oscillators utilizing four-layer diodes* M.S. thesis, Oklahoma State University, (1962).

18 Izhikevich, E. M. Which model to use for cortical spiking neurons? *IEEE Trans. Neural Netw.* **15**, 1063-1070 (2004).

19 Izhikevich, E. M. *Dynamical systems in neuroscience: The geometry of excitability and bursting*. (The MIT Press, 2007).

20 Izhikevich, E. M., Desai, N. S., Walcott, E. C. & Hoppensteadt, F. C. Bursts as a unit of neural information: selective communication via resonance. *Trends Neurosci.* **26**, 161-167 (2003).
21 Ando, T. et al. CMOS compatible MIM decoupling capacitor with reliable sub-nm EOT high-k stacks for the 7 nm node and beyond. *2016 IEEE Int. Electron Dev. Meeting* https://doi.org/10.1109/IEDM.2016.7838382 (2016).
22 Kim, S., Du, C., Sheridan, P., Ma, W., Choi, S. & Lu, W. D. Experimental demonstration of a second-order memristor and its ability to biorealistically implement synaptic plasticity. *Nano Lett.* **15**, 2203−2211 (2015).
23 Oh, D.-W., Ko, C., Ramanathan, S. & Cahill, D. G. Thermal conductivity and dynamic heat capacity across the metal-insulator transition in thin film VO2. *Appl. Phys. Lett.* **96**, 151906 (2010).
24 Berglund, C. N. G., H. J. Electronic properties of VO2 near the semiconductor-metal transition. *Phys. Rev.* **185**, 1022-1033 (1969).
25 Eyert, V. The metal-insulator transitions of VO2: A band theoretical approach. *Ann. Phys.* **11**, 650-702 (2002).
26 Pickett, M. D., Medeiros-Ribeiro, G. & Williams, R. S. A scalable neuristor built with Mott memristors. *Nat. Mater.* **12**, 114-117 (2013).
27 Crane, H. D. The neuristor. *IRE Trans. Elect. Comput.* **9**, 370-371 (1960).
28 Borghetti, J. et al. Oscillator circuitry having negative differential resistance. US Patent 8,324,976 B2. (2012).
29 Becker, M. F. et al. Femtosecond laser excitation of the semiconductor-metal phase transition in VO2. *Appl. Phys. Lett.* **65**, 1507-1509 (1994).
30 Cavalleri, A. et al. Femtosecond structural dynamics in VO2 during an ultrafast solid-solid phase transition. *Phys. Rev. Lett.* **87**, 237401 (2001).
31 Yang, Z., Ko, C. & Ramanathan, S. Oxide electronics utilizing ultrafast metal-insulator transitions. *Annu. Rev. Mater. Res.* **41**, 337-367 (2011).
32 Lim, H. et al. Reliability of neuronal information conveyed by unreliable neuristor-based leaky integrate-and-fire neurons: a model study. *Sci. Rep.* **5**, 09776 (2015).
